# Bioinformatics screening of colorectal-cancer causing molecular signatures through gene expression profiles to discover therapeutic targets and candidate agents

**DOI:** 10.1186/s12920-023-01488-w

**Published:** 2023-03-29

**Authors:** Md Abu Horaira, Md. Ariful Islam, Md. Kaderi Kibria, Md. Jahangir Alam, Syed Rashel Kabir, Md. Nurul Haque Mollah

**Affiliations:** 1 Bioinformatics Lab, Department of Statistics, University of Rajshahi, Rajshahi, 6205 Bangladesh; 2 Department of Biochemistry and Molecular Biology, University of Rajshahi, Rajshahi, 6205 Bangladesh

**Keywords:** Colorectal cancer (CRC), Gene expression profiles, Receptor proteins, Drug agents, Integrated bioinformatics analyses

## Abstract

**Background:**

Detection of appropriate receptor proteins and drug agents are equally important in the case of drug discovery and development for any disease. In this study, an attempt was made to explore colorectal cancer (CRC) causing molecular signatures as receptors and drug agents as inhibitors by using integrated statistics and bioinformatics approaches.

**Methods:**

To identify the important genes that are involved in the initiation and progression of CRC, four microarray datasets (GSE9348, GSE110224, GSE23878, and GSE35279) and an RNA_Seq profiles (GSE50760) were downloaded from the Gene Expression Omnibus database. The datasets were analyzed by a statistical r-package of LIMMA to identify common differentially expressed genes (cDEGs). The key genes (KGs) of cDEGs were detected by using the five topological measures in the protein–protein interaction network analysis. Then we performed in-silico validation for CRC-causing KGs by using different web-tools and independent databases. We also disclosed the transcriptional and post-transcriptional regulatory factors of KGs by interaction network analysis of KGs with transcription factors (TFs) and micro-RNAs. Finally, we suggested our proposed KGs-guided computationally more effective candidate drug molecules compared to other published drugs by cross-validation with the state-of-the-art alternatives of top-ranked independent receptor proteins.

**Results:**

We identified 50 common differentially expressed genes (cDEGs) from five gene expression profile datasets, where 31 cDEGs were downregulated, and the rest 19 were up-regulated. Then we identified 11 cDEGs (*CXCL8, CEMIP, MMP7, CA4, ADH1C, GUCA2A, GUCA2B, ZG16, CLCA4, MS4A12* and *CLDN1*) as the KGs. Different pertinent bioinformatic analyses (box plot, survival probability curves, DNA methylation, correlation with immune infiltration levels, diseases-KGs interaction, GO and KEGG pathways) based on independent databases directly or indirectly showed that these KGs are significantly associated with CRC progression. We also detected four TFs proteins (FOXC1, YY1, GATA2 and NFKB*)* and eight microRNAs (hsa-mir-16-5p, hsa-mir-195-5p, hsa-mir-203a-3p, hsa-mir-34a-5p, hsa-mir-107, hsa-mir-27a-3p, hsa-mir-429, and hsa-mir-335-5p) as the key transcriptional and post-transcriptional regulators of KGs. Finally, our proposed 15 molecular signatures including 11 KGs and 4 key TFs-proteins guided 9 small molecules (Cyclosporin A, Manzamine A, Cardidigin, Staurosporine, Benzo[A]Pyrene, Sitosterol, Nocardiopsis Sp, Troglitazone, and Riccardin D) were recommended as the top-ranked candidate therapeutic agents for the treatment against CRC.

**Conclusion:**

The findings of this study recommended that our proposed target proteins and agents might be considered as the potential diagnostic, prognostic and therapeutic signatures for CRC.

**Supplementary Information:**

The online version contains supplementary material available at 10.1186/s12920-023-01488-w.

## Introduction

Colorectal cancer (CRC) is an uncontrolled cell growth in the colon, rectum or appendix. It is the second most commonly diagnosed cancer in females and the third in males. The world health organization (WHO) reported in 2018 that over 1.8 million new cases and nearly 862,000 deaths due to CRC worldwide [1, 2]. With more than 2.2 million new cases and 1.1 million fatalities, the global incidence of CRC is projected to be increased 60% by 2030 [3]. The early stages of CRC symptoms are uncharacteristic and frequently ignored or misdiagnosed. Importantly, CRC is diagnosed at the middle or late stages of the disease. It is characteristically identified at the middle or late stages of the disease. The fecal-based examination, enteroscopy and blood-based examination are commonly considered the early detection methods for CRC [4]. However, several instrument-dependent detection methods are time-consuming, laborious and expensive. The leading treatment options for CRC are surgery, adjuvant chemotherapy (for colon cancer), neoadjuvant radiotherapy (for rectal cancer), and molecular drugs [5, 6]. However, these types of treatments have several drawbacks. According to the previous studies, less than 15% of metastatic CRC is suitable for surgery, the spreading rate of CRC exceeds more than 80% within 3 years after surgery, and the spreading rate exceeds more than 95% within 5 years after surgery [7]. Although there are some advancement in the case of CRC treatments, the 5-year survival time of patients with this disease has not yet increased significantly [6]. Therefore, the identification of new molecular biomarkers is essential for CRC diagnosis, prognosis and new therapies.

However, new drug discovery is a tremendously challenging, time-consuming and expensive task. The main challenges are to explore drug target proteins (receptors) responsible for the diseases and drug agents (small molecules) that can reduce the diseases by the interaction with the target proteins. Genomic biomarkers induced proteins are considered as the key receptors. Transcriptomics analysis is a widely used popular approach to explore genomic biomarkers [8–13]. The repurposing of existing drugs for certain diseases could reduce the time and cost compared to de novo drug development. By this time, several authors have suggested different sets of key genes (KGs) to explore molecular mechanisms and pathogenetic processes of CRC progression [14–45] in which some studies have employed multiple datasets to identify CRC-causing KGs [15–17, 22, 25, 26, 31, 32, 37, 40–43, 46–49]. Few studies also explored their suggested KGs-guided candidate drug molecules for the treatment against CRC [14, 37, 40–42, 50–59]. However, their published data did not display any common KGs as well as common drug molecules (see Additional file 1: Table S1) in all studies. None of those studies investigated the resistance performance of their suggested KGs-guided drug molecules against the CRC-causing independent KGs suggested by others. We found CRC-causing 170 different KGs and associated 64 different drug molecules in those articles. The articles those suggested therapeutics agents applied enrichment approach on Cmap, geneXpharma or DGIdb databases to select the KGs-guided candidate agents for the treatment against CRC [14, 37, 40–42, 50–59]. They did not provide pairwise drug-target binding affinity scores, since enrichment techniques cannot calculate pairwise binding scores. So, it may be difficult to select most potential drug-target pairs from the existing studies for experimental validation by the wet-lab researchers. On the other hand, though the total number of KGs 170 is much smaller than the whole genome size, it may be yet much laborious, time consuming and costly for the experimental validation of more than 170 × 64 = 10,880 drug-target pairs by the wet-lab researchers. Therefore, in this study, our main objectives were to explore (1) more probable CRC-causing KGs from multiple gene expression profile datasets through the verification with different benchmark datasets and independent databases and (2) proposed KGs-guided candidate drug molecules for the treatment of CRC through the verification of their resistance power against the CRC-causing top-ranked independent KGs suggested by others, by molecular docking analysis.

## Materials and methods

**Fig. 1:**
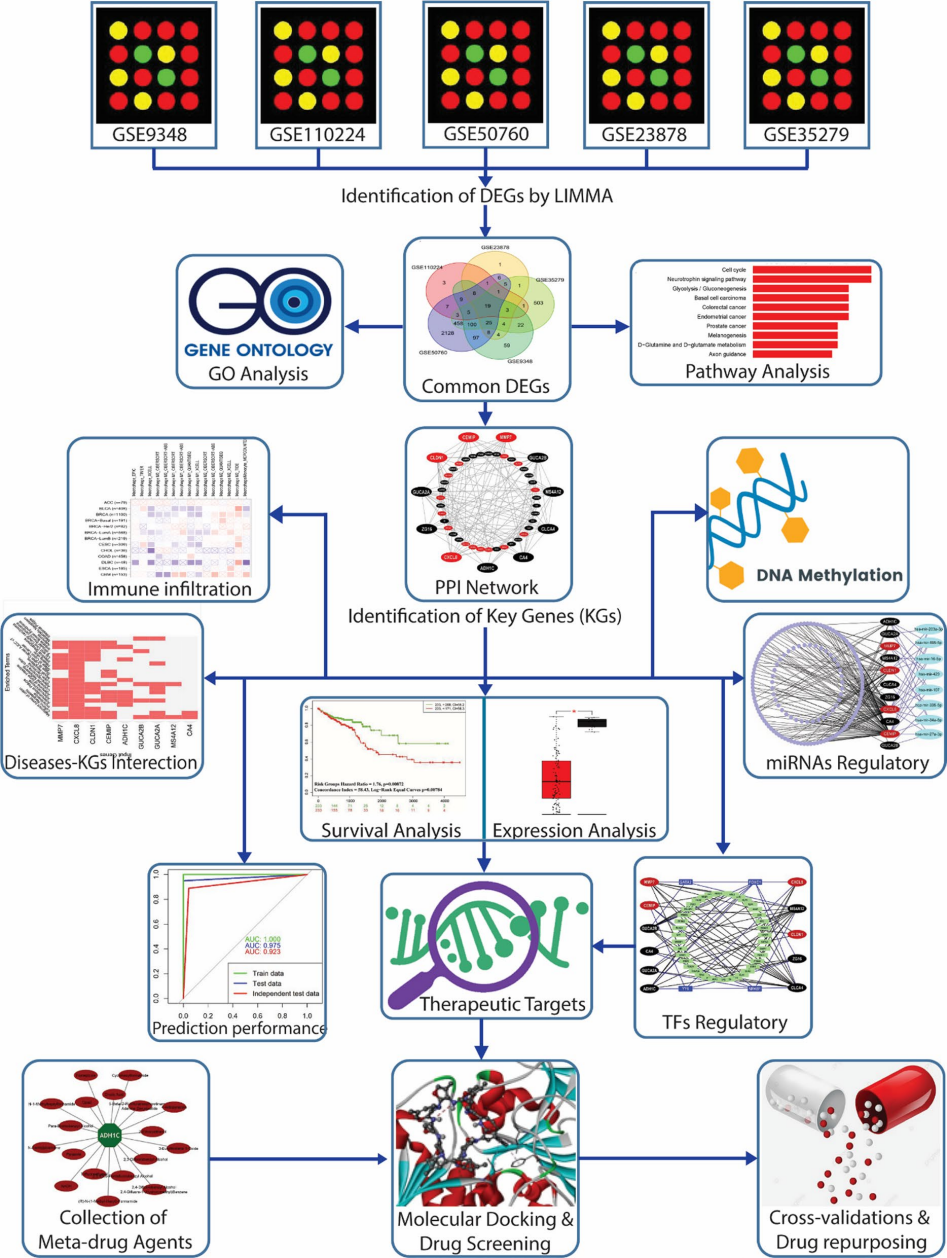
The pipeline of this study

The overview of this study including materials and methods is summarized in Fig. 1.

### Data sources and descriptions

We collected gene expression profiles generated from CRC patients for exploring drug targets and small molecules (drug agents) for exploring candidate drugs by molecular docking simulation as described below.

#### Collection of gene expression profiles for exploring drug-target proteins (receptors)

Four human CRC microarray datasets (GSE9348, GSE110224, GSE23878, and GSE35279) and one RNA-Seq dataset (GSE50760) were downloaded from National Center for Biotechnology Information (NCBI) Gene Expression Omnibus (GEO) database (https://www.ncbi.nlm.nih.gov/geo/). The platform of GSE9348, GSE110224, and GSE23878, were GPL570 [HG-U133_Plus_2] (Affymetrix Human Genome U133 Plus 2.0 Array), GSE35279 was performed by GPL6480 (Agilent-014850 Whole Human Genome Microarray 4 × 44 K G4112F) and GSE50760 was performed by GPL11154 Illumina HiSeq 2000 (Homo sapiens). The summary of this dataset is given in Table 1.

**Table 1:** Details of gene expression profiles that we analyzed

GEO accession	Platform	Year	Country	Normal (n)	Tumor (n)
GSE9348	GPL570	2010	Singapore	12	70
GSE35279	GPL6480	2013	Japan	5	74
GSE23878	GPL570	2010	Saudi Arabia	24	35
GSE110224	GPL570	2018	Greece	17	17
GSE50760	GPL11154	2014	South Korea	18	18

#### Collection of meta-drug agents for exploring candidate drugs

We collected meta-drug agents from the online database DSigDB [60] with respect to the proposed receptors and FDA approved repurposed drugs for the treatment of CRC patients.

#### Collection of independent meta-receptors for cross-validation with the proposed drugs

To select the top-ranked receptor proteins (meta-receptors) associated with CRC, we reviewed 33 recently published articles and selected the top-ranked 8 target proteins as the meta-receptors (see Additional file 1: Table S1).

### Integrated statistics and bioinformatics approaches

To reach the goal of this study, we applied both statistical and bioinformatics approaches, as discussed below in detail.

#### Identification of DEGs by using LIMMA

To identify differentially expressed (DEGs) between tumor and normal conditions, we considered the linear models for microarray (LIMMA) data analysis suggested by Smith [61], which can be written as

1 $$z_{g} = Y\theta_{g} + u_{g}$$

where $z_{g} = \left( {z_{g1} ,z_{g2} , \ldots ,z_{gn} } \right)^{/}$ is the vector of expressions (responses) for gth gene with n = n_1_ + n_2_ samples (g = 1, 2, …, m), ***Y*** is an n × 2 design matrix, $\theta_{g} = \left( {\theta_{g1} ,\theta_{g2} } \right)^{/}$ is 2 × 1 vector (2 < n) of effects for two different groups of n samples, and the error vector $u_{g} \sim N(0, W_{g} \sigma_{g}^{2}$). Here $W_{g}$ is a positive definite weight matrix. We want to test the null hypothesis (H_0_): $\theta_{g1} = \theta_{g2} = > \gamma_{g} = (\theta_{g1} - \theta_{g2} ) = 0$ (that is, *g*th gene is equally expressed gene (EEG) in both case and control groups) against the alternative hypothesis (H_1_): $\theta_{g1} \ne \theta_{g2} = > \gamma_{g} \ne 0$ (that is, the *g*th gene is a DEG between case and control groups). To test H_0_ against H_1_, the moderated t-statistic was formulated by hybridizing the classical and Bayesian approaches in which the posterior variance is substituted into the classical t-statistic in place of the classical sample variance. The moderated t-statistic was defined as

2 $$\tilde{t}_{g} = \frac{{\hat{z}_{g} - z_{g} }}{{\tilde{s}_{g} \sqrt {\delta_{g} } }},$$

which follows t-distribution with $d_{g} + d_{0}$ degrees of freedom under H_0_.

Adjusted *P* values based on the moderated t-statistics and the average of log fold-change (aLog2FC) values of the treatment group with respect to the control group were used to select DEGs as follows

3 $${\text{DEG}}_{{\text{g}}} = \left\{ {\begin{array}{*{20}l} {DEG \left( {{\text{Upregulated}}} \right),} \hfill & {if\quad adj.p.value\left\langle {0.01\,\, and\,\, aLogFC} \right\rangle 1.0} \hfill \\ {DEG \left( {{\text{Downregulated}}} \right),} \hfill & {if\quad adj.p.value < 0.01\,\, and\,\, aLogFC < - 1.0} \hfill \\ \end{array} } \right.$$

where

$${\text{aLog}}2{\text{FC}} = \left\{ {\begin{array}{*{20}l} {\frac{1}{{n_{1} }}\mathop \sum \limits_{i}^{{n_{1} }} \log 2(z_{{g_{i} }}^{T} ) - \frac{1}{{n_{2} }}\mathop \sum \limits_{j}^{{n_{2} }} \log 2(z_{{g_{j} }}^{C} ),} \hfill & { if\quad n_{1} \ne n_{2} } \hfill \\ {\frac{1}{n}\mathop \sum \limits_{i}^{n} \log 2\left( {\frac{{z_{{g_{i} }}^{T} }}{{z_{{g_{j} }}^{C} }}} \right), } \hfill & {if\quad n_{1} = n_{2} = n} \hfill \\ \end{array} } \right.$$

Here $z_{{g_{i} }}^{T}$ and $z_{{g_{j} }}^{C}$ are the expressions for the gth gene with the ith treatment and jth control samples, respectively. We implemented this algorithm using LIMMA r-package to calculate the *P* values [62] and aLogFC values to select the DEGs significantly from four gene expression datasets as introduced previously. We separated upregulated and downregulated DEGs for each of four datasets. Then we selected common upregulated and downregulated DEGs for all of four datasets. Then we combine common upregulated DEGs and common downregulated DEGs to construct the common DEGs (cDEGs) set.

#### Construction of PPI network to identify CRC-causing key genes (KGs)

Protein–protein interaction (PPI) network was constructed to identify common key-genes (KGs). The online STRING-v11 [63] database was used to construct the PPI network of cDEGs. The String database provides critical assessment and integration of protein interactions, including direct (physical) and indirect (functional) associations. To construct a PPI network, the distance ‘D’ between pair of proteins (u,v) is calculated as

4 $$D\left( {u,v} \right) = \frac{{2\left| {N_{u} \cap N_{v} } \right|}}{{|N_{u} \left| { + |N_{v} } \right|}}$$

where *N*_*u*_ is the neighbor set of *u* and *N*_*v*_ is the neighbor set of *v*. Cytoscape plugin cytoHubba was used to rank the nodes of the PPI network, which could be utilized to identify KGs in the network [64, 65]. In the present study, five topological methods, including Degree [66], BottleNeck [67], Betweenness [68], and Stress [69] was utilized to identify KGs.

#### In-silico validation of CRC-causing KGs

An attempt was made to validate the CRC-causing KGs by using different web-tools and independent databases as introduced below.

##### Expression analysis for KGs by GEPIA web-tool with TCGA RNA-seq data

To validate the expression levels of key genes, a gene expression profiling interactive analysis (GEPIA) tool (http://gepia.cancer-pku.cn/) was used to explore the related data in TCGA databases, and to analyse the expression levels of key genes in CRC tissues compared with normal tissues [70].

##### Association of KGs with the immune infiltration levels in different cancers including CRC

Tumor Immune Estimation Resource (TIMER) is an integrative resource for investigating the molecular characterization of tumor-immune interactions across various cancer types (https://cistrome.shinyapps.io/timer/) [71]. TIMER utilizes a deconvolution statistical method to deduce the abundance of six tumor-infiltrating immune cells, including B cells, CD4^+^ T cells, CD8^+^ T cells, macrophages, neutrophils and DCs from The Cancer Genome Atlas (TCGA).

##### DNA methylation of KGs

MethSurv is used to explore methylation biomarkers associated with the survival of various human cancers [72]. MethSurv is freely available at https://biit.cs.ut.ee/methsurv. Through the MethSurv website, we will analyze the DNA methylation analysis of the selected CRC-related genes in the TCGA database.

##### Association of KGs with different disease

The Disease-KGs enrichment analysis was performed using the Enrichr web tool [73] with DisGeNET database [74] to explore other disease risk factors for CRC patients.

##### Prognostic power analysis of KGs

To investigate the prognostic power of KGs, we performed cluster analysis, survival analysis and developed two prediction models using random forest (RF) and AdaBoost classifiers. The survival curve and ROC curve were used to assess the prognosis performance. The online SurvExpress computational tool [75] was used to produce a survival curve. The r-packages ‘gplots’ and ‘ROCR’ were used to produce heatmap and ROC curve, respectively. Exploring drugs by molecular docking simulation.

##### Exploring GO and KEGG pathway terms that are associated with DEGs including KGs

The GO (Gene Ontology) functions [76] and KEGG (Kyoto Encyclopedia of Genes and Genomes) pathway enrichment analysis [77] were performed to explore CRC-causing ontology terms (Biological Process (BP), Cellular Component (CC), and Molecular Function (MF)) and pathways that are associated with cDEGs including KGs. To explore the significantly enriched GO terms and KEGG pathways by cDEGs including KGs, let *S*_*i*_ is the annotated gene-set corresponding to the *i*th type of biological functions or pathways given in the database, and *M*_*i*_ is the number of genes in *S*_*i*_ (*i* = 1, 2,…,*r*); *N* is the total number of annotated genes those construct the entire combine set $S = \mathop \cup \limits_{i = 1}^{r} S_{{\text{i}}} = S_{i} \cup S_{i}^{c}$ such that $N \le \mathop \sum \limits_{i = 1}^{r} M_{i} ;$ where $S_{i}^{c}$ is the complement set of *S*_*i*_. Again, let *n* is the total number of cDEGs of interest and *k*_*i*_ is the number of cDEGs belonging to the annotated gene-set *S*_*i*_. This problem is summarized by the following contingency table (Table 2):

**Table 2:** Contingency table

Annotated gene-sets (given in the GO terms or KEGG pathway databases)	cDEGs (proposed)	CEEGs (proposed)	Marginal total
*i*th GO term/KEGG pathway (*S*_*i*_)	*k*_*i*_	*M*_*i*_ *−* *k*_*i*_	*M*_*i*_
Complement of *S*_*i*_ ($S_{i}^{c}$)	*n* *−* *k*_*i*_	*N* *−* *M*_*i*_ *−* *n* + *k*_*i*_	*N* *−* *M*_*i*_
Marginal total	*n*	*N* *−* *n*	*N* (Grand total)

To find the significantly enriched GO terms and KEGG pathways by our proposed cDEGs, the *P* value was calculated by the Fisher exact test statistic based on the hypergeometric distribution. We used Enrichr online tool to perform Fisher exact test [78].

#### Regulatory network analysis of KGs

To identify key transcription factors (TFs) as the transcriptional regulators of KGs, the TFs-KGs interaction network was constructed using the publicly available database JASPAR [79]. The interaction network was generated using NetworkAnalyst [80]. To identify key microRNAs (miRNAs) as the post-transcriptional regulators of KGs, the KGs-miRNAs interaction network was constructed by using the publicly available online tool TarBase v8.0 (Release 7.0) [81]. The top degree miRNAs were selected from the networks (miRNAs-KGs) and considered as key miRNAs.

#### Molecular docking simulation for exploring candidate drug agents

To explore efficient FDA approved repurposed drugs for the treatment of CRC patients, we employed molecular docking simulation between the target receptor proteins and drug agents. We considered our proposed KGs based hub-proteins and associated TFs proteins as the drug target receptor proteins and meta-drug agents collected from online databases and published articles for docking analysis. The molecular docking simulation requires 3-Dimensional (3D) structures of both receptor proteins and candidate drugs. We downloaded the 3D structure of all targeted receptor proteins from Protein Data Bank (PDB) [82] and SWISS-MODEL [66]. The 3D structures of drug agents were downloaded from the PubChem database [83]. The 3D structure of the target proteins was visualized using Discovery Studio Visualizer 2019 [84], and the water molecules, co-crystal ligands which were bound to the protein were removed. Further, the protein was prepared using Swiss-PdbViewer [85] and AutoDock Vina [86] in PyRx open-source software by adding charges and minimizing the energy of the protein and subsequently converting it to pdbqt format [86, 87]. The exhaustiveness parameter was set to 8. The Discovery Studio Visualizer 2019 was used to analyze the docked complexes for surface complexes, types and distances of non-covalent bonds. Let *A*_*ij*_ denotes the binding affinity between *i*th target protein (*i* = 1, 2, …, *m*) and *j*th drug agent (*j* = 1, 2, …, *n*). Then target proteins are ordered according to the descending order of row sums $\sum\nolimits_{j = 1}^{n} {A_{ij} }$, *j* = 1, 2, …,* m*, and drug agents are ordered according to the descending order of column sums $\sum\nolimits_{i = 1}^{m} {A_{ij} }$, *j* = 1,2, …, *n*, to select the top ranking few drug agents as the candidate drugs. Then we validated the proposed repurposed drugs by molecular docking simulation with the top ordered independent receptor proteins associated with CRC published by others.

## Results

### Identification of cDEGs

**Fig. 2:**
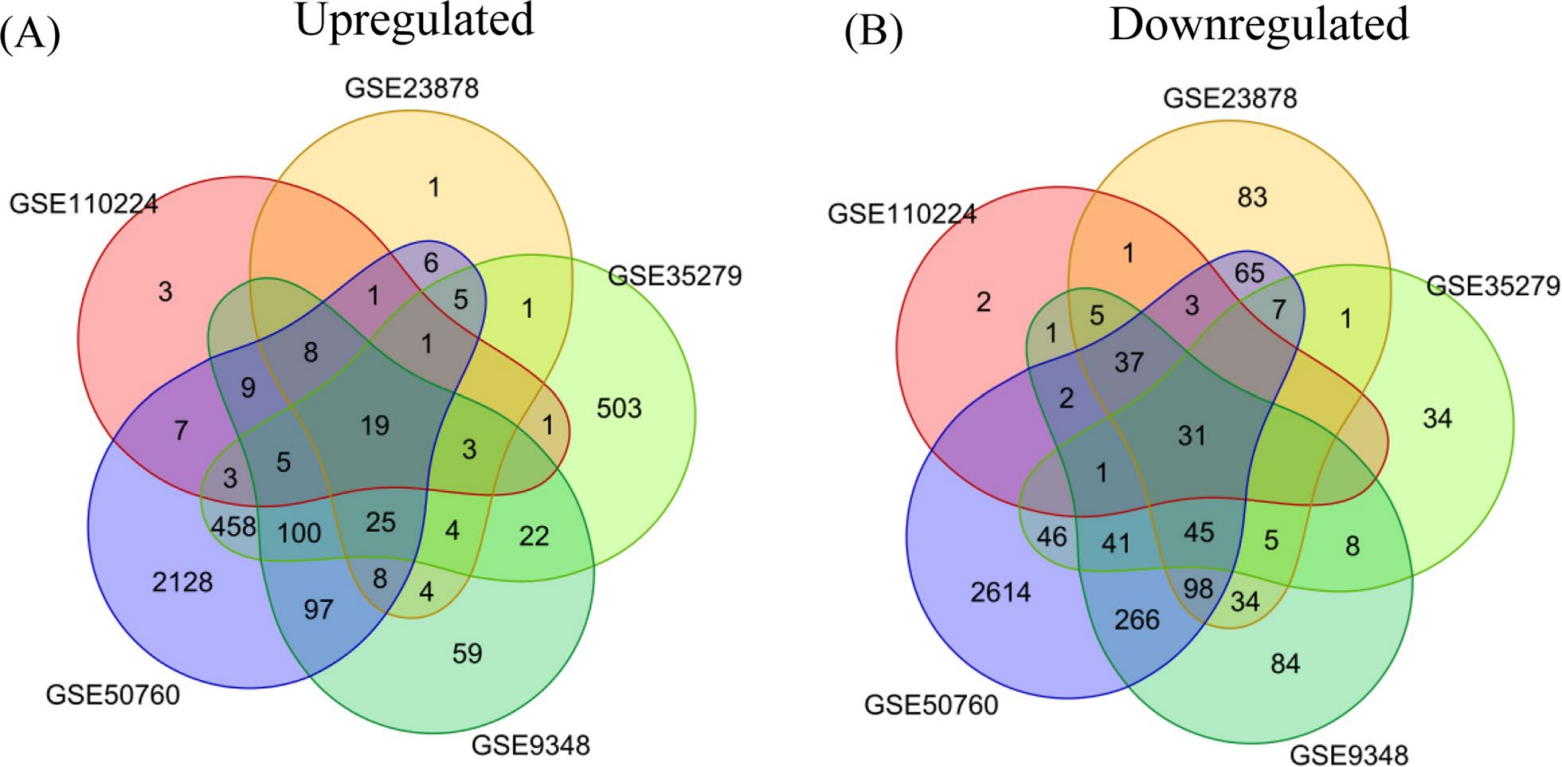
Common DEGs (cDEGs) among the five GEO datasets for **A** up-regulated and **B** downregulated

**Fig. 3:**
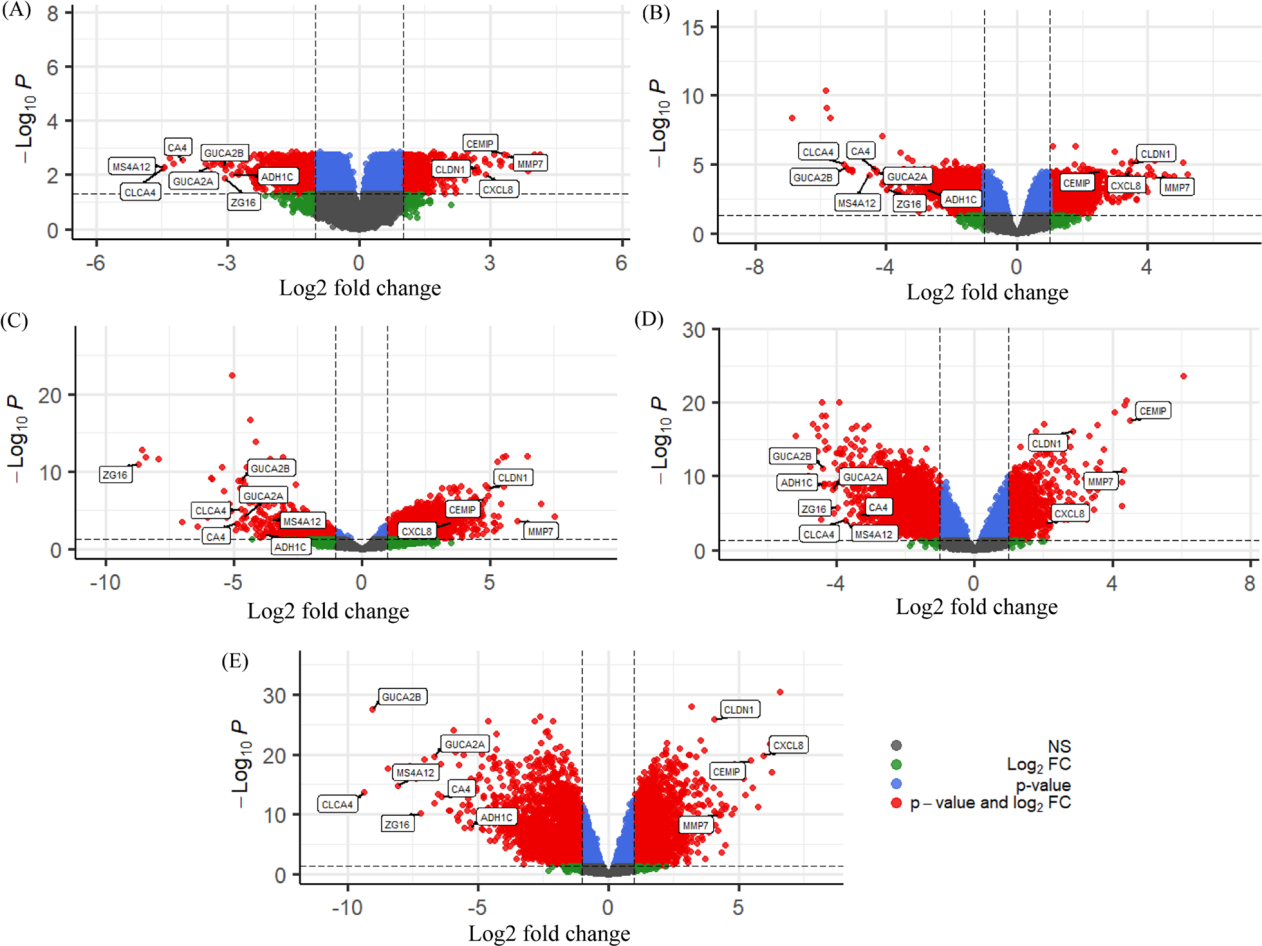
The five GEO datasets volcano plots of **A** GSE110224, **B** GSE50760, **C** GSE35279, **D** GSE23878 and **E** GSE9348. Ass color point are Not Significant (NS) according to Log_2_FC and *P* value threshold, green color is Log_2_FC (Log_2_FC < − 1 and Log_2_FC > 1), blue color is *P* value ≤ 0.05, and red color points are satisfying the Log_2_FC and *P* value threshold

We identified 50 cDEGs, including 19 up-regulated (Fig. 2A) and 31 down-regulated (Fig. 2B) genes in CRC tissue, using adj.*P*.Val < 0.01 and logFC > 1 as the threshold for down-regulated cDEGs, and adj.*P*.Val < 0.01 and logFC < -1 for up-regulated cDEGs. The down and up regulated cDEGs were displayed on the right and left sides respectively in the volcano plot (Fig. 3 and Additional file 2).

### Identification of key genes (KGs) from cDEGs

**Fig. 4:**
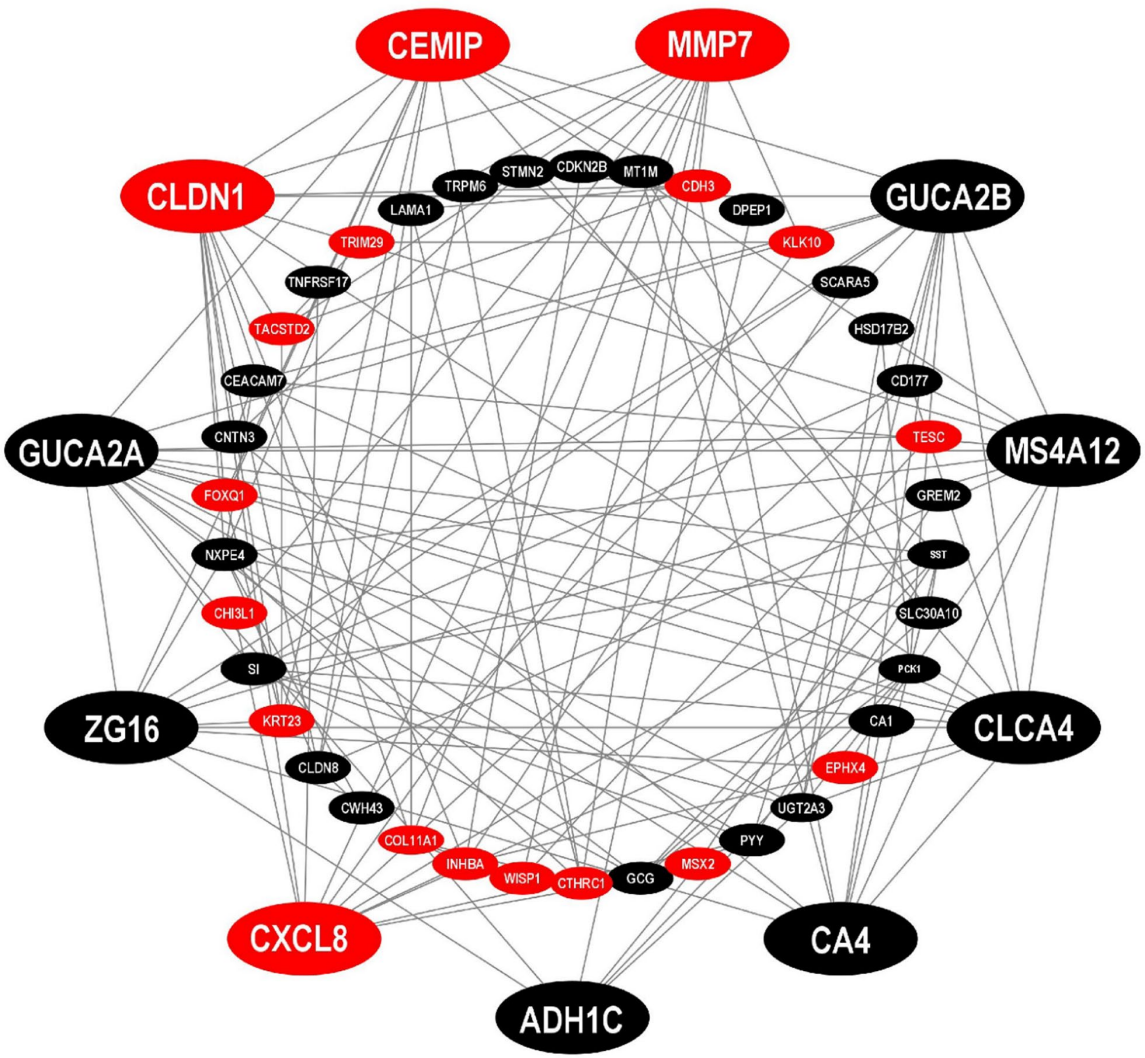
Network of PPIs for common cDEGs that have been identified. Red color nodes and upregulated and black color nodes are downregulated. The outer circle of the image is common key genes (KGs)

The PPI network of cDEGs was constructed using the STRING database, which includes 49 nodes and 175 edges, with an average node degree of 6.73 and *P* value < 1.0e−16. In the PPI network, Red color indicates up-regulated and black color indicates down-regulated cDEGs, big size and octagon shape indicate common key genes (KGs) (Fig. 4). We used four topological measures (Degree, BottleNeck, Betweenness, and Stress) to select top-ranked 11 KGs (Table 3) that are *CXCL8, MMP7, CA4, ADH1C, GUCA2A, GUCA2B, CEMIP, ZG16, CLCA4, MS4A12 and CLDN1,* where 4 KGs (*CXCL8, CEMIP, CLDN1,* and *MMP7*) were up-regulated and the rest 7 KGs were downregulated.

**Table 3:** Selection of KGs by combining the top ranked genes of five topological measurements with the PPI network

Degree (A)	BottleNeck (B)	Betweenness (C)	Stress (D)	Key genes (KGs) (${\varvec{A}} \cup {\varvec{B}} \cup {\varvec{C}} \cup {\varvec{D}})$
*GUCA2A*	*CLDN1*	*CXCL8*	*CLDN1*	*GUCA2A, GUCA2B, CLDN1, CLCA4, MS4A12, MMP7, CEMIP, CXCL8, ADH1C, ZG16, CA4*
*GUCA2B*	*CXCL8*	*CLDN1*	*GUCA2A*	
*CLDN1*	*CLCA4*	*GUCA2A*	*GUCA2B*	
*CLCA4*	*MMP7*	*MMP7*	*CXCL8*	
*MS4A12*	*ZG16*	*CA4*	*CA4*	

### In-silico validation of CRC-causing KGs by using different web-tools and independent databases

#### Expression analysis for KGs by GEPIA web-tool with TCGA RNA-seq data

**Fig. 5:**
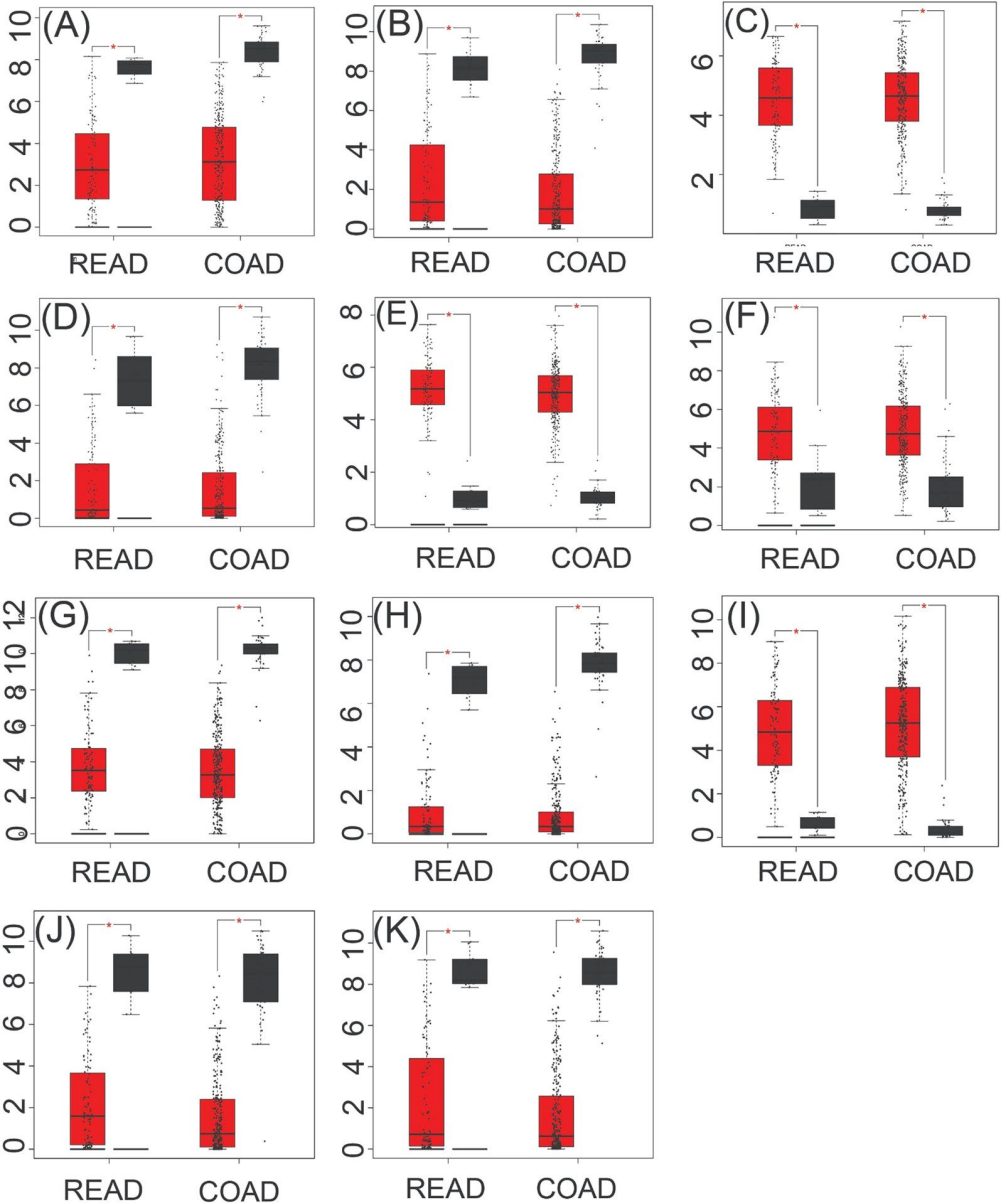
The expression level of hub genes in CRC. **A** ADH1C; **B** CA4; **C** CEMIP; **D** CLCA4; **E** CLDN1; **F** CXCL8; **G** GUCA2A; **H** GUCA2B; **I** MMP7; **J** MS4A12 and **K** ZG16. The red and gray boxes represent cancer and normal tissues, respectively. Colon adenocarcinoma (COAD) and rectum adenocarcinoma (READ)

In the GEPIA database, differences in transcriptional expression of the hub gene between CRC tissues and normal tissues were again verified. Combining with the box plot results, eleven potential KGs further were screened out. Based on the GEPIA database to test the relative expression of KGs mRNA, it was determined that our proposed KGs (*CXCL8, CEMIP, MMP7, CA4, ADH1C, GUCA2A, GUCA2B, ZG16, CLCA4, MS4A12* and *CLDN1*) may be closely related to the occurrence and development of CRC (Fig. 5).

#### Correlation between KGs and immune infiltration levels in different cancers including CRC

We investigated the relationship of different tumors ininfiltrates immune cell types (B cell, CD8 T cell, CD4 T cell, neutrophil, macrophage and dendritic cell (DC)) with the expressions of KGs (Additional file 3). We observed (Additional file 4) that our proposed KGs are significantly associated with different tumor infiltrates immune cells under different databases of COAD (colon adenocarcinoma) and READ (Rectum adenocarcinoma). Compelling evidence has demonstrated that tumor-infiltrating lymphocytes are significantly associated with survival in cancer. Therefore, we investigated whether KGs expression was related to immune infiltration levels in lung cancer by TIMER. Tumor purity is an important factor affecting the analysis of immune infiltration. Interestingly, our results indicated that KGs expression was correlated with poor prognosis and high immune infiltration in CRC. KGs were highly expressed in monocytes (non-classical and classical) and B cells (naïve). In contrast, KGs expression was not significantly correlated with tumor purity or infiltrating levels of CD8^+^ T cells, CD4^+^ T cells or neutrophils in CRC. CA4 expression levels were positively correlated with infiltrating levels of B cells (r = 0.22, *P* = 2.47E−04), CD8^+^ T cells (r = − 0.16, *P* = 7.49E−03), CD4^+^ T cells (r = 0.19, *P* = 1.99E−03), macrophages (r = − 0.18, *P* = 2.19E−03), neutrophils (r = 0.38, *P* = 4.6E−11) and DCs (r = 0.23, *P* = 9.73E−05) in COAD (Additional files 3 and 4). CLCA4 expression levels were also positively correlated with infiltrating levels of B cells (r = 0.32, *P* = 1.92E−03), CD8^+^ T cells (r = − 0.23, *P* = 2.95E−02), CD4^+^ T cells (r = 0.39, *P* = 1.36E−04), macrophages (r = − 0.48, *P* = 8.42E−07), neutrophils (r = 0.5, *P* = 4.50E−07) and DCs (r = 0.33, *P* = 1.10E−03) in READ (Additional file 3 and 4). These findings strongly suggest that KGs plays an important role in immune infiltration in CRC, especially infiltration of Macrophage, T cell CD8^+^, T cell CD4^+^, Neutrophil, Myeloid dendritic cell, and B cell.

#### DNA methylation of KGs

DNA methylation at CpG (CG) sites play the vital role in cancer progression. Therefore, we investigated DNA methylation of KGs (*CXCL8, CEMIP, MMP7, CA4, ADH1C, GUCA2A, GUCA2B, ZG16, CLCA4, MS4A12* and *CLDN1*) at CpG sites by MethSurv web-tool with TCGA database. We observed that seven KGs (*CEMIP, MMP7, CA4, GUCA2B, ZG16, CLCA4, MS4A12*) are significantly methylated at CpG sites (Table 4). The hypermethylation/downregulation gene *CEMIP* has six CpG sites with a *P* value < 0.05, the hypomethylation/upregulation gene *GUCA2B* has four CpG sites with a *P* value of < 0.05, the hypomethylation/upregulation gene *MS4A12* has two CpG sites with a *P* value < 0.05, and the hypomethylation/upregulation gene *MMP7, CLCA4, ZG16* has one CpG site with a *P* value of < 0.05, which is statistically significant (Table 4). We found that the difference in DNA methylation between CG12358698 of *CEMIP*, CG23532119 of *MS4A12*, CG00656728 of GUCA2B, CG24963041 of *MMP7*, CG26310643 of *CLCA4*, CG09229061 of *ZG16,* CG00200645 of CA4 and CG07510230 of *ZNRF2* was most pronounced.

**Table 4:** The significant prognostic value of CpG in three key genes

Gene-CpG	HR	*P* value
*CEMIP*-Body-Open_Sea-CG12358698	2.657	0.001
*CEMIP*-Body-Open_Sea-CG12098156	2.275	0.001
*CEMIP*-Body-Open_Sea-CG04847610	1.899	0.008
*CEMIP*-Body-Open_Sea-CG17820039	3.085	0.027
*CEMIP*-Body-Open_Sea-CG21838329	2.665	0.045
*CEMIP*-5'UTR-Open_Sea-CG09579081	3.836	0.019
*MMP7*-TSS1500-Open_Sea-cg24963041	1.822	0.016
*MS4A12*_5'UTR-Open_Sea-cg09257456	0.196	0.003
*MS4A12_*TSS200-Open_Sea-cg23532119	4.164	0.009
*CLCA4*-Body-Island-cg26310643	0.259	0.018
*ZG16*-TSS1500-Open_Sea-cg09229061	6.022	0.001
*CA4*-Body-Open_Sea-cg00200645	3.173	0.022
*GUCA2B*-TSS1500-Open_Sea-cg00656728	11.395	0.001
*GUCA2B*-TSS200-Open_Sea-cg10179693	3.585	0.009
*GUCA2B*-TSS200-Open_Sea-cg14848143	3.185	0.023
*GUCA2B*-1stExon-Open_Sea-cg19728577	7.457	0.001

#### Association of KGs with different diseases including CRC

**Fig. 6:**
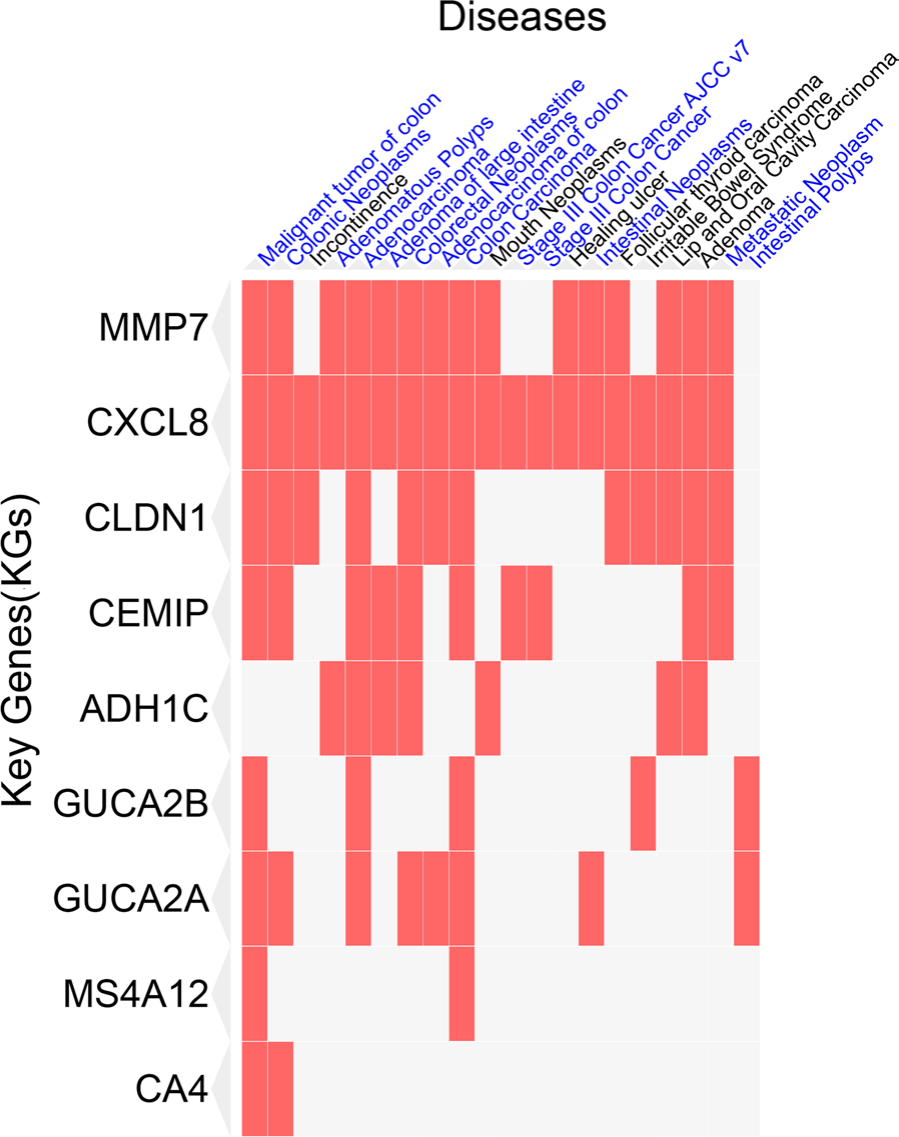
KGs-Diseases interaction, where blue color highlighted risk factors are CRC related

The disease-KGs interaction analysis showed that KGs are significantly associated with different types of colon or rectal cancers including Malignant tumor of colon, Colonic Neoplasms, Adenomatous Polyps, Adenocarcinoma, Adenoma of large intestine, Colorectal Neoplasms, Adenocarcinoma of colon, Colon Carcinoma, Stage III Colon Cancer AJCC v7, Stage III Colon Cancer, Intestinal Neoplasms, Adenoma and Metastatic Neoplasm (Fig. 6 and Table S2 in Additional file 1).

#### Prognostic power analysis

**Fig. 7:**
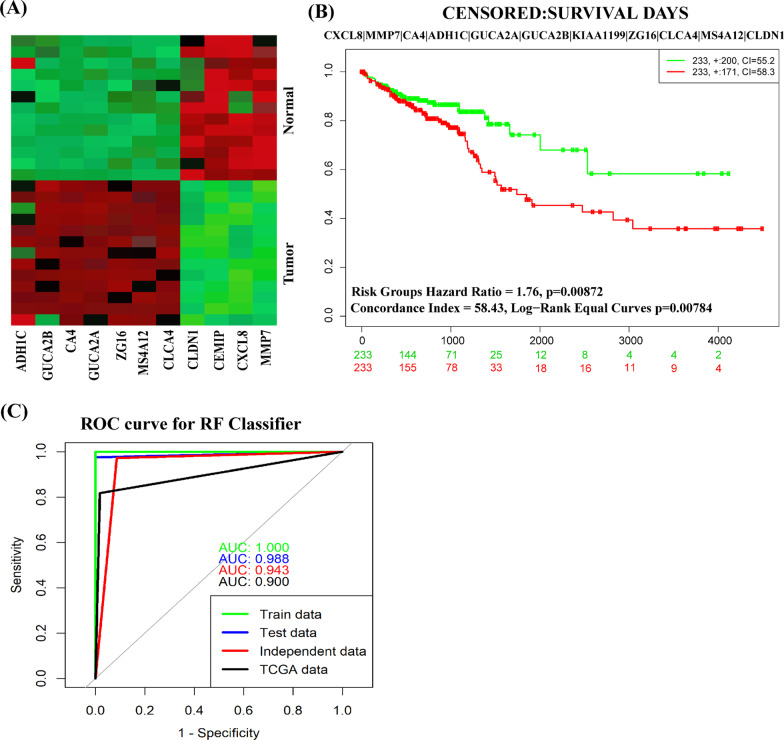
The prognostic powers of KGs were displayed by **A** a Heatmap of hierarchical clustering, **B** multivariate survival curves with KGs, and **C** ROC curves of prediction models with KGs

We considered both supervised and unsupervised learnings, including multivariate survival analysis, to investigate the prognostic power of KGs. Figure 7A shows that KGs can separate case and control samples accurately by the unsupervised hierarchical clustering (HC). The multivariate survival curves, based on the expressions of 11 KGs, separated the low and high-risk groups significantly (Fig. 7B). In the case of supervised learning, at first, we considered the expression profiles of 11 KGs from three datasets (GSE9348, GSE23878 and GSE110224) that contained 60 tumors and 50 control samples in total. Then we partitioned these datasets in to training (70%) and test (30%) sets. Then we trained one popular classifier known as random forest (RF). To test the prediction performance of the model, we also considered the expressions of 11 KGs from another two dataset GSE35279 and TCGA as the independent test set. Figure 7C showed the ROC curves based on the train, test performance, and independent test dataset of RF prediction model. The AUC values (area under the ROC curve) for RF were 1.00 with train data, 0.988 with test data, 0.943 with independent test data and 0.90 with TCGA dataset. Thus, both prediction models based on RF classifiers showed good performance for each of the dependent and independent test datasets of KGs.

#### Exploring CRC-causing GO and KEGG pathway terms that are associated with cDEGs including KGs

The GO functional enrichment analysis of showed that 185 GO-BP terms, 9 GO-CC terms and 38 GO-MF terms are enriched by the cDEGs genes, where KGs were involved with 57 BPs, 6 CCs and 21 MFs. Among the enriched GO functions including KGs, 6 GO-BP terms (GO:0034,31 ~ cell junction maintenance, GO:0098742 ~ cell–cell adhesion via plasma-membrane adhesion molecules, GO:0045216 ~ cell–cell junction organization, GO:0008285 ~ negative regulation of cell population proliferation, GO:0030334 ~ regulation of cell migration, and GO:0048565 ~ digestive tract development), 5 GO-CC terms (GO:0046658 ~ anchored component of plasma membrane, GO:0062023 ~ collagen-containing extracellular matrix, GO:0005923 ~ bicellular tight junction, GO:0043296 ~ apical junction complex, and GO:0005911 ~ cell–cell junction) and 6 GO-MF terms (GO:0005179 ~ hormone activity, GO:0030250 ~ guanylate cyclase activator activity, GO:0048018 ~ receptor ligand activity, GO:0005254 ~ chloride channel activity, GO:0008237 ~ metallopeptidase activity, and GO:0045236 ~ CXCR chemokine receptor binding) were reported by other researchers as the BPs of CRC (see Table 3 and discussion section for more details). The KEGG pathway enrichment analysis of cDEGs showed that 8 pathways are enriched by the KGs. Among them, KGs involving Nitrogen metabolism, Proximal tubule bicarbonate reclamation, Cell adhesion molecules, Pathogenic *Escherichia coli* infection, Human T-cell leukemia virus 1 infection, Amoebiasis, Leukocyte transendothelial migration, and Cytokine-cytokine receptor interaction was also reported by other researchers as the pathways of CRC development (see Table 5 and discussion section for more details as before).

**Table 5:** Top Enriched gene ontology (GO) terms and KEGG pathways by the proposed cDEGs highlighting cKGs

Term	Overlap	*P* value	cKGs
*Biological process*			
Cell junction maintenance (GO:0034331) [88]	2/14	6.14E−04	*CLDN1*
Cell–cell adhesion via plasma-membrane adhesion molecules (GO:0098742) [89]	4/170	0.001067	*CLDN1*
Calcium-independent cell–cell adhesion via plasma membrane cell-adhesion molecules (GO:0016338) [89]	2/20	0.00127	*CLDN1*
Cell–cell junction organization (GO:0045216) [89]	3/82	0.001342	*CLDN1*
Negative regulation of cell population proliferation (GO:0008285) [90]	5/379	0.003239	*CXCL8*
Regulation of cell migration (GO:0030334) [91]	5/408	0.00443	*MMP7;CLDN1*
*Molecular function*			
Hormone activity (GO:0005179) [92]	5/78	1.96E−06	*GUCA2A*
Guanylate cyclase activator activity (GO:0030250) [93]	2/5	6.85E−05	*GUCA2B;GUCA2A*
Receptor ligand activity (GO:0048018) [94]	6/307	1.56E−04	*GUCA2A*
Chloride channel activity (GO:0005254) [95]	2/64	0.012507	*CLCA4*
Metallopeptidase activity (GO:0008237) [96]	2/121	0.040942	*MMP7*
CXCR chemokine receptor binding (GO:0045236) [97]	1/17	0.044125	*CXCL8*
*Cellular component*			
Anchored component of plasma membrane (GO:0046658) [98]	2/46	0.006619	*CA4*
Collagen-containing extracellular matrix (GO:0062023) [99]	4/380	0.018081	*ZG16*
Bicellular tight junction (GO:0005923) [100]	2/78	0.018197	*CLDN1*
Apical junction complex (GO:0043296) [101]	2/98	0.027852	*CLDN1*
Cell–cell junction (GO:0005911) [102]	3/271	0.035073	*CLDN1*
*KEGG*			
Nitrogen metabolism [22]	1/7	9.13E−04	*CA4*
Proximal tubule bicarbonate reclamation [103]	1/23	0.001682	*CA4*
Cell adhesion molecules [104]	3/148	0.007098	*CLDN1*
Pathogenic Escherichia coli infection [105]	3/197	0.015369	*CXCL8;CLDN1*
Amoebiasis [44]	2/102	0.029984	*CXCL8*
Leukocyte transendothelial migration [106]	2/114	0.036749	*CLDN1*
Cytokine-cytokine receptor interaction [107]	3/295	0.043339	*CXCL8*

### Regulatory network analysis of KGs

**Fig. 8:**
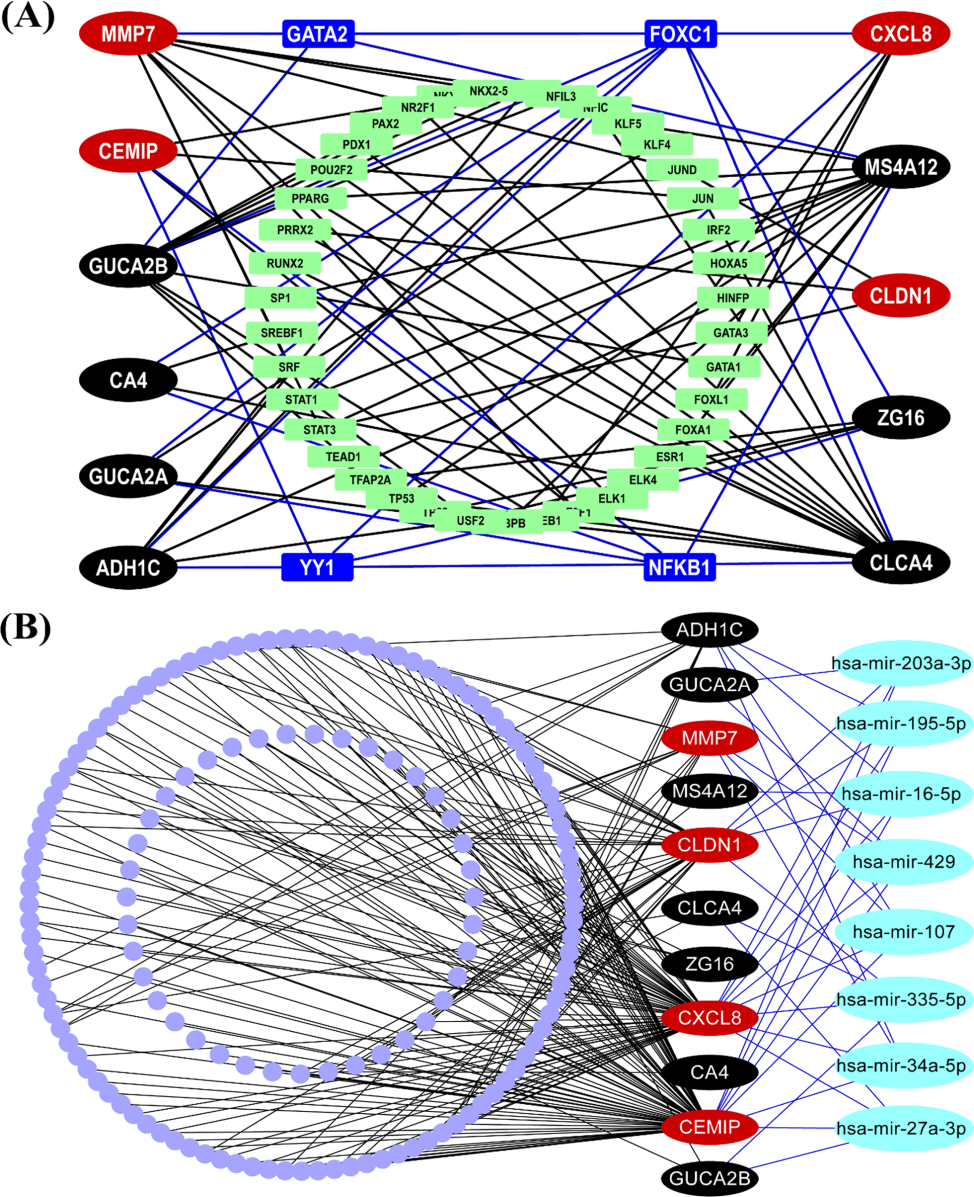
KGs regulatory network analysis results **A** KGs-TFs interaction network to identify key transcriptional regulators of KGs, **B** KGs-miRNAs interaction network to identify key post-transcriptional regulators of KGs. Here red and black color ellipse indicates the KGs in both A and B, green color bigger size rectangle indicates key TFs in **A** and key miRNAs in **B**

We constructed KGs versus transcription factors (KGs-TFs) interaction network to identify top ranking few TFs as the key transcriptional regulators of KGs. We selected the top 4 key TFs (FOXC1, YY1, GATA2 and NFKB1) as the vital transcriptional regulators of KGs with degree ≥ 4, where the green color rectangle indicates top degree key TFs and, red and black color ellipse indicates KGs (Fig. 8A). To identify top ranking few micro-RNA (miRNA) as the key post-transcriptional regulators of KGs, we constructed a KGs-miRNAs interaction network. We selected the top 8 key miRNAs (hsa-mir-16-5p, hsa-mir-195-5p, hsa-mir-203a-3p, hsa-mir-34a-5p, hsa-mir-107, hsa-mir-27a-3p, hsa-mir-429, and hsa-mir-335-5p) as the vital regulators of KGs with degree ≥ 4, where green color rectangle indicates top degree key miRNAs and, red and black color ellipse indicates KGs (Fig. 8B).

### Exploring candidate drug agents by molecular docking simulation

**Fig. 9:**
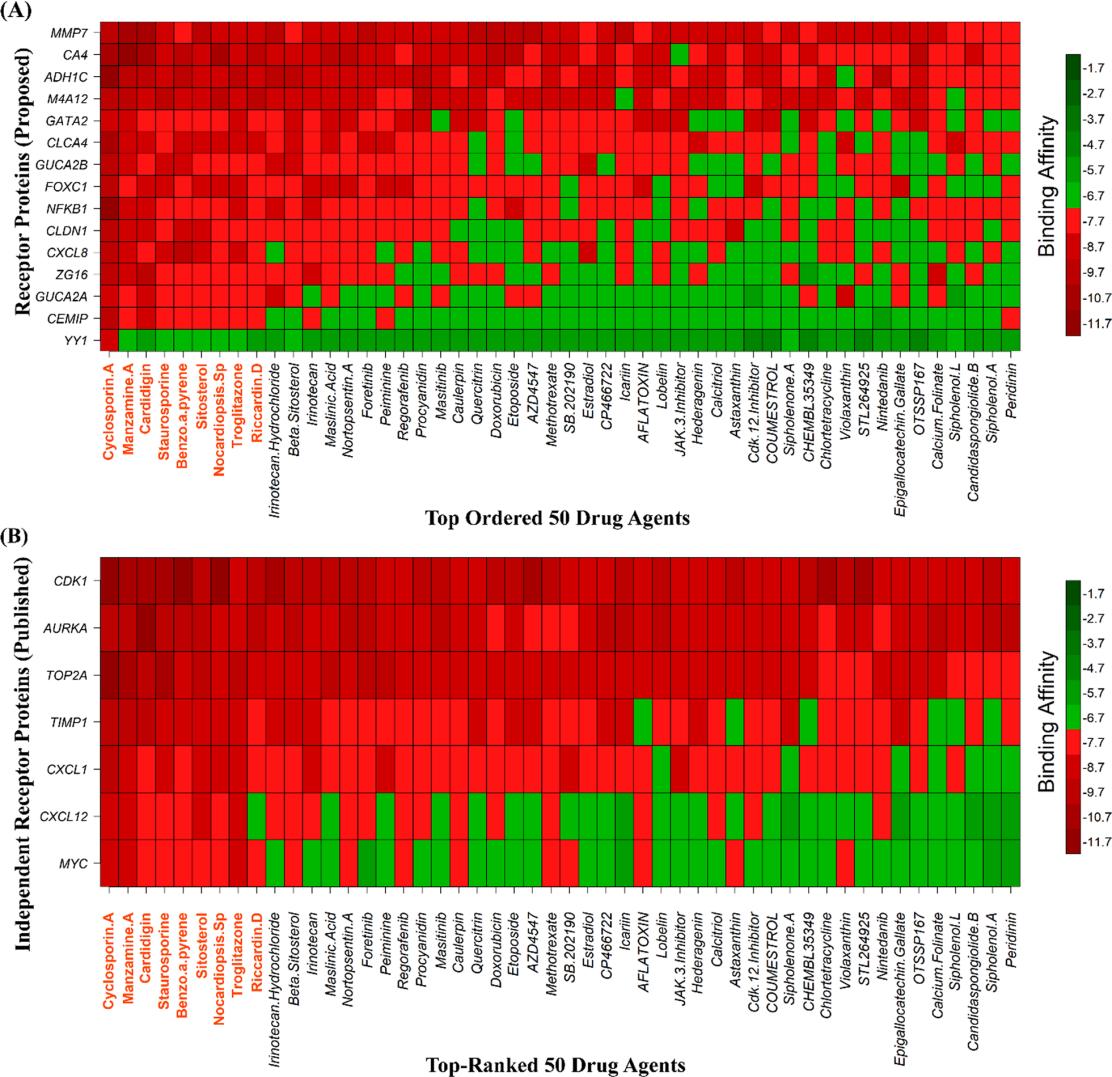
Molecular docking simulation results for exploring candidate drugs against CRC. **A** Image of binding affinity scores of proposed ordered receptor proteins with the top 50 ordered. **B** Image of binding affinity scores of the top-ranked independent receptors published by others with the top 50 ordered

To explore candidate drugs for CRC, we considered 11 KGs based proteins (CXCL8, MMP7, CA4, ADH1C, GUCA2A, GUCA2B, CEMIP, ZG16, CLCA4, MS4A12 and CLDN1) and its regulatory key 4 TFs proteins (FOXC1, YY1, GATA2 and NFKB1) as the m = 15 drug target receptors. The 3-Dimension (3D) structure of CXCL8, MMP7, ZG16, CA4, YY1 and NFKB1 were downloaded from Protein Data Bank (PDB) with the PDB codes 6N2U, 1MMQ, 3APA, 5KU6, 4C5I and 1NFI and the rest of them, such as GUCA2A, GUCA2B, CLDN1, CLCA4, MS4A12, FOXC1 and GATA2 targets were downloaded from SWISS-MODEL using UniProt with IDs Q02747, Q16661, O95832, Q14CN2, Q9NXJ0, Q12948, and P23769 respectively. Then we considered 92 meta-drug molecules from the DSigDB database and 64 meta-drugs from the published articles and the Food and Drug Administration (FDA) as drug agents. The 3D structures of drug agents were downloaded from the PubChem database. Then we performed a molecular docking simulation between our proposed receptors and meta-drug agents. The binding affinity score matrix between the ordered receptors and ordered drug agents were displayed in Fig. 9A. We observed that Cyclosporin A produces highly significant binding affinity scores with all m = 15 target proteins, and their average binding affinity scores across all targets were − 9.46 (kcal/mol). The 2th and 3th top ordered drugs (Manzamine A and Cardidigin) produced highly significant binding affinity scores with 14 target proteins, and their average binding affinity scores across all m = 15 targets were − 8.22 and − 8.19, respectively. The 4th to 10th top ordered drug Staurosporine, Benzo[A] Pyrene, Sitosterol, Nocardiopsis Sp, Troglitazone, K-252a, and Riccardin D produced significant binding affinity scores with 14 target proteins, and the average binding affinity score was − 7.76, − 7.71, − 7.69, − 7.68, − 7.66, − 7.64, and − 7.62 respectively. The other drugs (lead compounds) produced significant binding affinity scores with less than 13 target proteins out of 15, and their average binding affinity scores were negatively smaller than − 7.5. Therefore, we considered the top ordered nine drugs (Cyclosporin A, Manzamine A, Cardidigin, Staurosporine, Benzo[A]Pyrene, Sitosterol, Nocardiopsis Sp, Troglitazone and Riccardin D) as the candidate drugs in our study. We also examined their complete interaction profile, including hydrogen bonds, hydrophobic, halogen/ salt Bridge and electrostatic interactions in Table 6.

**Table 6:** The 3-dimension view of strong binding interactions between targets and drugs is shown in the 4th column

Potential targets	Structure of lead compounds	Binding affinity (kCal/mol)	The 3d view and interactions of complex	Interacting amino acids		
				Hydrogen bond	Hydrophobic interactions	Electrostatic
MMP7	Cardidigin	− 10.4		LEU181, THR189, ASN179, GLU219	HIS218, PHE103, PHE185	–
CA4	Manzamine A	− 10.8		HIS4, TYR11, HIS4	HIS4, HIS4	–
ADH1C	Cyclosporin A	− 11.7		ALA317	LEU116, ILE318, PHE93	–

Key interactions amino acids and their binding types with potential targets were shown in the last column

#### Performance investigation of proposed drugs by cross-validation with the top-ranked independent receptors

To investigate the resistance performance of our proposed 9 candidate drugs against the state-of-the-art alternative receptors for CRC by molecular docking, we considered the top-ranked 8 independent receptors (MYC, CDK1, CXCL1, CXCL8, CXCL12, TIMP1, AURKA, and TOP2A) published by others in different 36 articles for CRC (see Additional file 1: Table S1), where the receptor CXCL8 was common with our proposed receptor. The 3D structure of MYC, CDK1, CXCL12, TIMP1, AURKA, and TOP2A was downloaded from the PDB database with the PDB codes 6G6K, 6GU2, 6SHR, 2J0T, 6VPM, AND 1ZXM, respectively and for another one CXCL1, downloaded from SWISS-MODEL using UniProt with ID P09341. The we performed molecular docking analysis of 7 independent receptors with all of 160 drug agents. Figure 9B showed that our proposed 9 candidate drugs are also detected as the independent receptor-guided top-ranked 9 drugs. Therefore, we can strongly recommend that the proposed drugs might be more effective candidates than the other drugs for the treatment against CRC.

#### Connectivity map (CMap) analysis to discover the mechanism of action of drug agents

**Fig. 10:**
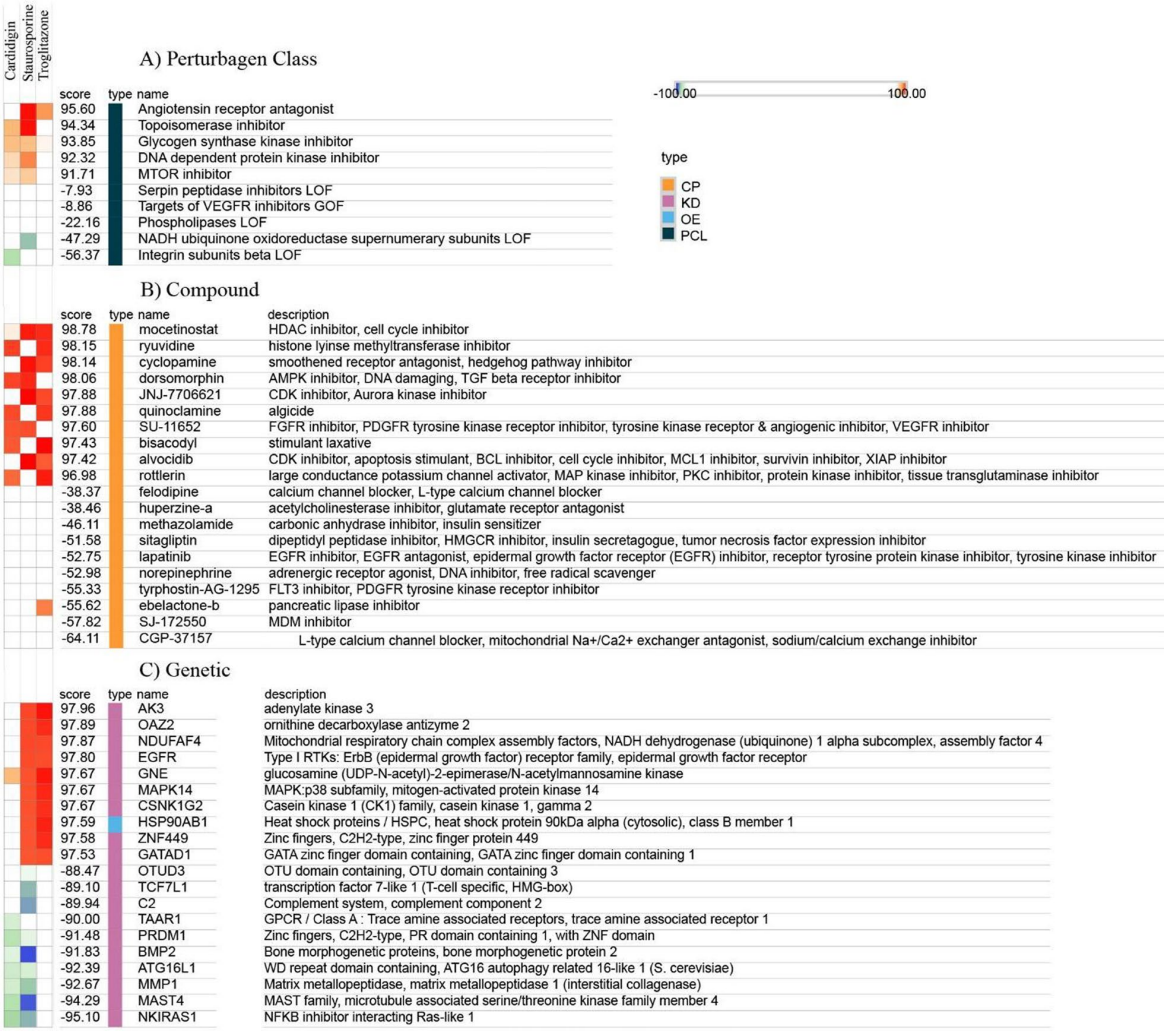
The Connectivity Map for three smalls molecules of Troglitazone, Cardidigin and Staurosporine

In an effort to elucidate its mechanism of action, we defined a signature for Troglitazone, Cardidigin and Staurosporine. High connectivity scores were found for multiple instances of five heat shock protein inhibitors: Angiotensin receptor antagonist, Topoisomerase inhibitor, Glycogen synthase kinase inhibitor, DNA dependent protein kinase inhibitor, and MTOR inhibitor. Despite the differences in the cells used to generate the query signature and reference profiles, the three highest-scoring compounds in the Con nectivity Map were Troglitazone, Cardidigin (Digitoxin use this name to use in Cmap) and Staurosporine (Fig. 10A). More important, the Connectivity Map also revealed strong connectivity with ten structurally distinct compounds, mocetinostat, ryuvidine, cyclopamine, dorsomorphin, JNJ-7706621, quinoclamine, SU-11652, bisacodyl, alvocidib, and rottlerin respective inhibitor are show in Fig. 10B. Cyclopamine and alvocidib compounds are not connected with Troglitazone.

*AK3, OAZ2, NDUFAF4, EGFR, GNE, MAPK14, CSNK1G2, HSP90AB1, ZNF449, and GATAD1* genes depicts high positive connectivity with each of the drugs Troglitazone, Cardidigin and Staurosporine and their median connectivity score belongs to 97.96–97.53 (out of ± 100) which display in the Fig. 10C with corresponding enriched pathways of the connected gene. Moreover, the drug staurosporine was positively connected with 6 other genes namely *OTUD3, TCF7L1, C2, TAAR1, PRDM1*, and *BMP2* with corresponding enriched pathways.

## Discussion

**Fig. 11:**
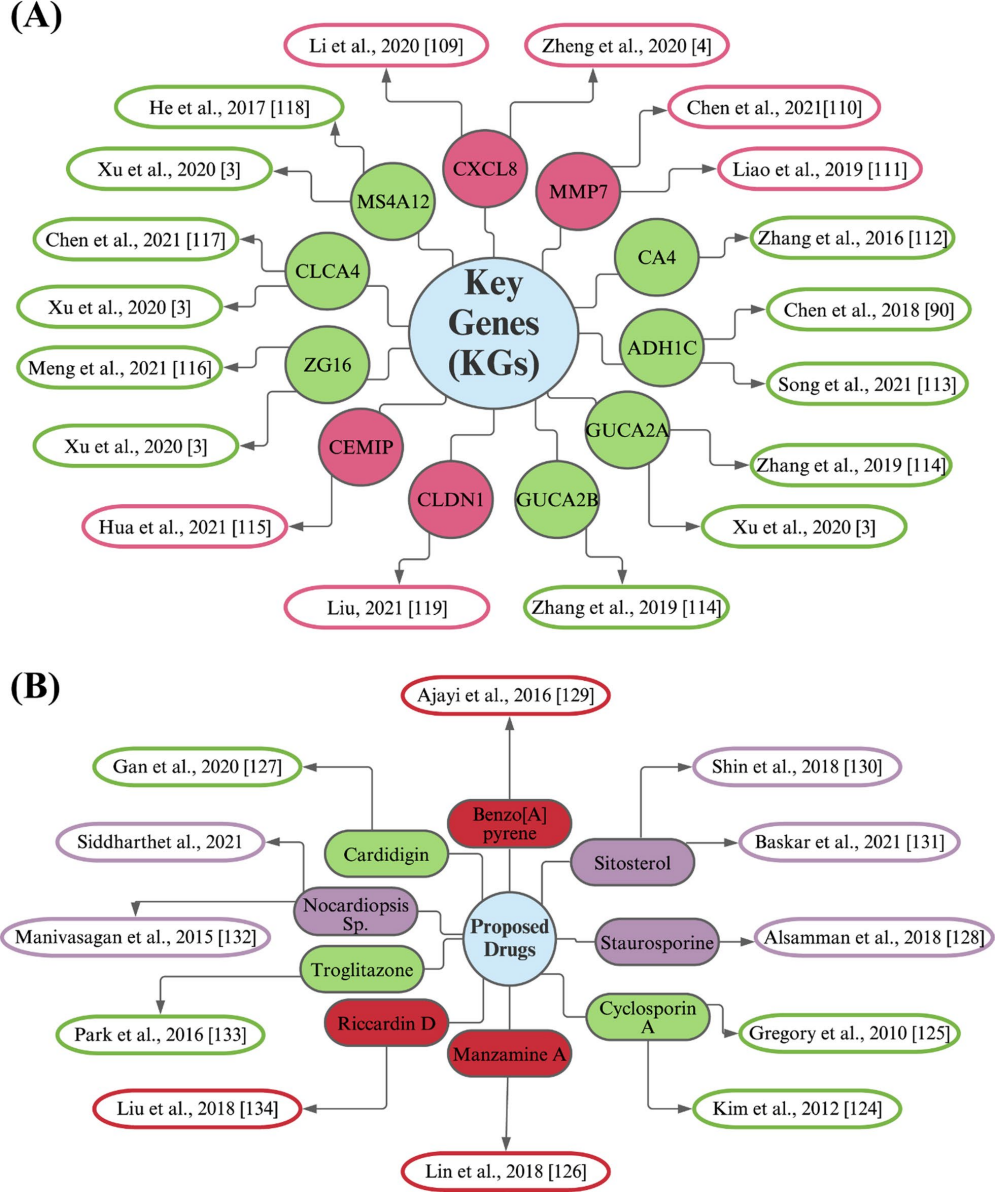
Validation of the proposed KGs (receptors) and candidate drugs in favor of CRC by the literature review. **A** Validation of the proposed KGs: circles with green color indicate downregulated KGs, and pink color indicates up-regulated KGs, and each connected network with a circle indicates the reference in which the cKG is associated with CRC, **B** validation of the proposed candidate drugs: circles with green color indicate FDA approved and investigational drugs, purple color indicate investigational drugs and red color indicate unapproved drugs and each connected network with a circle indicates the references in which our suggested drugs might be effective against CRC treatment

The molecular mechanism of colorectal cancer (CRC) is not yet completely clear to the researchers. So potential molecular signatures are required to disclose molecular mechanisms of CRC and its therapeutic agents. The integrated statistics and bioinformatics analyses are now widely using to explore potential molecular signatures of malignant tumors [108]. Transcriptomics analysis is a popular way of identifying DEGs between normal and tumor tissue samples [109]. Therefore, in this study, we considered the integrated bioinformatics analyses for exploring common genomic biomarkers from five transcriptomics profiles (GSE9348, GSE35279, GSE23878, GSE110224 and GSE50760) for diagnosis, prognosis, and therapies of CRC. At first, we identified 11 KGs (*CXCL8, MMP7, CA4, ADH1C, GUCA2A, GUCA2B, CEMIP, ZG16, CLCA4, MS4A12*, and *CLDN1*) by PPI analysis of 50 common DEGs (cDEGs). Some literature reviews also agreed with our results that these KGs are associated with CRC [14–45, 54] (see Fig. 11A). For example, Li et al. [110] reported that the gene *CXCL8* plays a vital role in CRC progression by mediating the differentiation, proliferation, and apoptosis within a regulatory network. So, they suggested this gene as a drug target for CRC also [110]. Chen and Ke [111] detected the gene *MMP7* as a potential biomarker of CRC by bioinformatics analysis. Another study found that it regulates cancer progression and mediate the differentiation, proliferation, invasion and metastasis of various cancer cell types by different mechanisms [112]. A study reported that *CA4* is a newly identified tumor suppressor gene in CRC by targeting the WTAP–WT1–TBL1 axis through the inhibition of the *Wnt* signaling pathway [113]. The gene *ADH1C* might lead the increasing production of proinflammatory mediators by decreasing its expressions in the ulcerative colitis colon through the activation of the STAT1/NF-κB signaling pathway [114].

The abnormally expressed peptide hormones GUCA2A and GUCA2B play as paracrine endogenous ligands for the guanylate cyclase-C (GUCY2C) receptor and help for the development of tumors in CRC by the association comparatively in lower levels with the disrupted intestinal homeostasis [115]. CEMIP is an adaptor protein of the O-GlcNAc transferase that can be reprogramming the glutamine metabolism through the reciprocal regulation of β-catenin and thereby promotes CRC metastasis. So, CRC metastasis can be prevented by the combinational inhibition of CEMIP and glutamine metabolism that would be a useful therapeutic strategy [116]. ZG16 can modulate the immune response in CRC by blocking the PD-L1 expression and the strong correlation designate ZG16 as a biomarker for the stratification of patent of immunotherapy [117]. Cancer-associated fibroblasts exosomes decreased the sensitivity of CRC towards the radiation and over-expressed miR-590-3p that promote CLCA4-dependent PI3K/Akt signaling pathway as well as cancer cell survival [118]. MS4A12 gene belonging to the MS4A family encodes a protein found in the apical membrane of colonocytes that plays an important role in the differentiation, proliferation, and cell cycle regulation and is believed to be a risk classification marker for early-stage colon cancer [119]. Upregulation of CLDN1 expression was observed in patients with colorectal cancer, which could be a possible biomarker for colorectal cancer treatment [120].

Moreover, different pertinent bioinformatic analyses based on independent databases significantly supported the relationship of KGs with the CRC progression as discussed below. The expression analysis with box-plots by GEPIA web-tool with TCGA RNA-seq data showed that KGs significantly separated CRC groups from the control groups (Fig. 5). We investigated the relationship of tumor infiltrates immune cells with the KGs and observed that KGs are significantly associated with different tumor infiltrates immune cells under different databases of CRC (Additional files 3 and 4). We investigated the DNA methylation of KGs at CpG sites by MethSurv web-tool with TCGA database and bserved that seven KGs (*CEMIP, MMP7, CA4, GUCA2B, ZG16, CLCA4, MS4A12*) are significantly methylated at CpG sites (Table 5) that may play the vital role in CRC progression. To investigate the prognostic power of KGs, we performed multivariate survival analysis and developed a prediction model through RF classifiers in Fig. 7C. Our developed prediction models showed good performance with both training and test datasets generated from the main data collected from NCBI with accession numbers GSE9348, GSE23878, and GSE110224. The AUC values were 1.000 for the training dataset and 0.988 for the test dataset for RF model. To investigate their performance unbiasedly, we also considered independent test datasets from other NCBI sources with accession numbers GSE35279, respectively. We observed that predictor show good performance with the independent test data and TCGA dataset. The values of AUC were 0.943 and 0.90 for independent test data and TCGA dataset based on RF model. These results indicate the good prediction performance for the identified KGs, so we suggested the prognostic model for the classifier (RF). The GO functional and KEGG pathway enrichment analyses of cDEGs significantly revealed some GO terms of BPs, MFs and CCs, and KEGG pathways by involving KGs that are highly linked with CRC patients (see Table 2). Our literature review also supported their link with CRC. As for examples with the enriched BPs, six GO terms *cell junction maintenance* [88], *cell–cell adhesion* [89], *calcium-independent cell–cell adhesion* [89], and *cell–cell junction organization* [89] these is associated with one KG (*CLDN1*). GO terms *negative regulation of cell population proliferation* [90] (associated with CXCL8), *regulation of cell migration* [91] (associated with MMP7 and CLDN1) and (associated with CA4) were reported as important BPs for CRC progression. Among the enriched MFs, two GO terms *hormone activity* [92] and *receptor ligand activity* [94] were associated with GUCA2A. The *guanylate cyclase activator activity* [93] were associated with GUCA2A, GUCA2B. The MFs terms *chloride channel activity* [95] was associated with CLCA4. The *metallopeptidase activity* [96] was associated with MMP7. Among the enriched CCs, *anchored component of plasma membrane* [98] was associated with CA4. *collagen-containing extracellular matrix* [99] was associated with ZG16. Four CCs term *bicellular tight junction* [100] *apical junction complex* [101] and *cell–cell junction* [102] were associated with CLCA1. Among the enriched KEGG pathways, two KEGG terms *Nitrogen metabolism* [22] and *Proximal tubule bicarbonate reclamation* [103] were associated with CA4. Three pathways *Cell adhesion molecules* [104], *Pathogenic Escherichia coli infection* [105] and *Leukocyte transendothelial migration* [106] were associated with CLDN1. Two pathways P*athogenic Escherichia coli infection* [105], *Amoebiasis* [44], and *Cytokine-cytokine receptor interaction* [107] were associated with CXCL8.

The KGs-TFs interaction network analysis indicated that 4 TFs proteins (FOXC1, YY1, GATA2 and NFKB1) are the key transcriptional regulatory factors of KGs (see Fig. 4A). Among them, FOXC1 (a regulator of *CA4, ADH1C, GUCA2A, GUCA2B, ZG16, and CLCA4*) is connected with lymphatic vessel formation, arterial cell specification, and cardiovascular development [121]. The expression of TF-protein YY1 (a regulator of *CXCL8, ADH1C, CEMIP, ZG16, and CLCA4*) contributes to tumor growth differs in different cancers [122]. The TF-protein GATA2 (a regulator of *GUCA2B, MMP7, CXCL8 and MS4A12*) is connected with Hematopoietic and immune defects [123]. The TF-protein NFKB1 (a regulator of *GUCA2A, CA4, CEMIP and MS4A12*) is a suppressor of inflammation, ageing and cancer [124]. We also constructed the proteins-disease interaction network to detect other diseases connected with the proposed target proteins. Total 9 target proteins out of 11 were associated with other 547 diseases that can be considered as the risk factors of CRC. Especially, two diseases, "Malignant tumor of colon" and "Colonic Neoplasms", were mostly related to our target proteins.

To explore our proposed KGs-guided new and repurposable candidate drugs for the treatment against CRC, we considered the proposed KGs based 11 key proteins (*CXCL8, MMP7, CA4, ADH1C, GUCA2A, GUCA2B, CEMIP, ZG16, CLCA4, MS4A12 and CLDN1*) and their regulatory 4 TFs proteins (FOXC1, YY1, GATA2 and NFKB1) as the drug target receptors and performed their docking simulation with 167 drug molecules collected from the DSigDB database and published articles (Fig. 9A). Then we selected top-ranked 10 drugs (Cyclosporin A, Manzamine A, Cardidigin, Staurosporine, Benzo[A]Pyrene, Sitosterol, Nocardiopsis Sp, Troglitazone, K-252a and Riccardin D) as the most probable repurposable candidate drugs for CRC patients based on their strong binding affinity scores (kCal/mol) with all the target proteins (Fig. 9A, B). Then we investigated the resistance performance of both the proposed and already published candidate drugs against the state-of-the-art alternatives of already published top-ranked 7 independent receptors for CRC and observed that our proposed candidate drugs are more effective compared to the already published drugs against the independent receptors also (Fig. 11B). We also tried to validate our proposed drugs in favor of CRC by the literature review (Fig. 11B).

Among the identified candidate drugs Cyclosporin A, a calcineurin inhibitor, traditionally used for its immunosuppressive effects, inhibits the activity of the noncanonical Wnt/Ca^++^/NFAT signaling pathway [125, 126]. It has been reported that Manzamine A exhibits an antiproliferative effect on human colorectal carcinoma cells and displays broad effects on gene expression to downregulate fundamental maintenances of cell survival and induce apoptotic cell death and EMT inactivation [127]. This study demonstrates the efficacy of Cardidigin (digitoxin) against cervical cancer in vivo and elucidates its molecular mechanisms, including DSBs, cell cycle arrest and mitochondrial apoptosis. These results will contribute to the development of Cardidigin as a chemotherapeutic agent in the treatment of cervical cancer [128]. Staurosporine alleviates cisplatin chemoresistance in human cancer (colon) cell models by suppressing the induction of SQSTM1/p62 [129]. Ajayi et al. [130] showed that Benzo[A] Pyrene induces oxidative stress, pro-inflammatory cytokines, expression of nuclear factor-kappa B, and deregulation of Wnt/β-catenin signaling in colons of exposed mice. Sitosterol (Beta-sitosterol) suppresses tumor growth without toxicity in AGS xenograft mouse models and induces apoptosis in human gastric adenocarcinoma cells [131]. Sitosterol prevents lipid peroxidation and improves antioxidant status and histoarchitecture in rats with 1,2-dimethylhydrazine-induced colon cancer [132]. The marine actinobacterium Nocardiopsis sp. MBRC-48 is an excellent microbial resource for the biosynthesis of gold nanoparticles with various biomedical applications such as antimicrobial, antioxidant, and anticancer activities [133]. The anti-proliferative and apoptotic activities of PDT in combination with the PPARγ ligand troglitazone and provide a strong rationale for testing the therapeutic potential of combination treatment in colon cancer [134]. Liu et al. showed that Riccardin D might inhibit cell proliferation and induce apoptosis in HT-29 cells, which may be associated with the blocking of the NF-κB signaling pathway [135]. Among the proposed nine candidate drugs, Cyclosporin A, Cardidigin, and Troglitazone are approved by the FDA for a different disease, the three other drugs (Staurosporine, Sitosterol and Nocardiopsis Sp.) are still investigational, and the rest of the three drugs (Manzamine A, Benzo[A]Pyrene, and Riccardin D) are not yet approved. The approved drugs for different diseases and unapproved drugs should be further assessed at the molecular level by the wet-lab experiments prior to clinical investigation in the treatment of CRC.

## Conclusion

The main purpose of this study was to identify key genomic biomarkers from multiple gene expression profiles for diagnosis, prognosis and therapies of CRC by using integrated bioinformatics and statistical approaches. We identified 11 common key genes (KGs) from multiple transcriptomics datasets, where 4 KGs (*CXCL8, CEMIP,* *MMP7,and CLDN1*) were up-regulated and the rest 7 KGs (*CA4, ADH1C, GUCA2A, GUCA2B, ZG16, CLCA4, and MS4A12)* were downregulated. Different pertinent bioinformatic analyses including box plots of KGs-expressions with CRC and control groups, multivariate survival probability curves based on KGs-expressions, DNA methylation of KGs, correlation of KGs with immune infiltration levels in CRC, (different diseases)-KGs interaction, CRC-causing GO and KEGG pathways based on independent databases significantly supported the relationship of KGs with the CRC progression. Their association was also supported by several other independent studies directly or indirectly that we mentioned in the discussion section. We detected four TFs proteins (FOXC1, YY1, GATA2 and NFKB*)* and eight microRNAs (hsa-mir-16-5p, hsa-mir-195-5p, hsa-mir-203a-3p, hsa-mir-34a-5p, hsa-mir-107, hsa-mir-27a-3p, hsa-mir-429, and hsa-mir-335-5p) as the key transcriptional and post-transcriptional regulators that may play a vital role in the regulation of KGs. Then we considered the proposed 11 key proteins and their regulatory 4 TFs-proteins as the drug target receptors to explore effective drug agents for CRC by molecular docking simulation with the 156 meta-drug agents. We detected nine small molecules (Cyclosporin A, Manzamine A, Cardidigin, Staurosporine, Benzo[A]Pyrene, Sitosterol, Nocardiopsis Sp, Troglitazone, and Riccardin D) as the top-ranked candidate drugs for the treatment against CRC. Then we investigated the resistance performance of the proposed drugs against the state-of-the-art already published top-ranked 11 independent receptors for CRC and observed that our proposed repurposable candidate drugs are more effective compared to the already published drugs against the independent receptors also. Therefore, the proposed candidate drugs might be played a vital role in the treatment of CRC.

## Supplementary Information

**Additional file 1. Table S1.** Different lists of hub-genes [HubGs] for colorectal cancer [CRC] published in different articles. **Table S2.** Association of cKGs with other disease risks.

**Additional file 2.** The details of common differentially expressed genes (cDEGs) for the five GEO datasets (GSE110224, GSE50760, GSE35279, and GSE23878) including Gene.symbol, ID, adj.P.Val, P.Value, t, B, logFC, and Gene.title.

**Additional file 3.** Correlation plots illustrating the relationship between gene expression and immune infiltration levels across multiple types of cancers, using the bioinformatics tool TIMER 2.0.

**Additional file 4.** Correlation between key genes (KGs) and immune infiltration levels in colorectal cancers (CRC).

## Data Availability

The necessary R-codes and the datasets analyzed in this study is available at the web link: https://github.com/Horaira29/crc.git. (We collected these datasets from the National Center of Biotechnology Information (NCBI) Gene Expression Omnibus (GEO) database with the accession numbers GSE9348, GSE110224, GSE23878, GSE35279 and GSE50760 using the web links. https://www.ncbi.nlm.nih.gov/geo/query/acc.cgi?acc=GSE9348, https://www.ncbi.nlm.nih.gov/geo/query/acc.cgi?acc=GSE110224, https://www.ncbi.nlm.nih.gov/geo/query/acc.cgi?acc=GSE23878, https://www.ncbi.nlm.nih.gov/geo/query/acc.cgi?acc=GSE35279, and https://www.ncbi.nlm.nih.gov/geo/query/acc.cgi?acc=GSE50760 respectively. We collected meta-drug agents from the online database DSigDB [56] with respect to the proposed receptors and FDA approved repurposed drugs for the treatment of NSCLC patients https://www.cancer.gov/about-cancer/treatment/drugs/colorectal).
