# Supplementary material for: Bioinformatics screening of colorectal-cancer causing molecular signatures through gene expression profiles to discover therapeutic targets and candidate agents
Source: BMC Med Genomics. 2023 Mar 29;16:64. doi: 10.1186/s12920-023-01488-w (PMC10053149; doi:10.1186/s12920-023-01488-w)
Supplement: Supplementary file 1 — Additional file 1. Table S1. Different lists of hub-genes [HubGs] for colorectal cancer [CRC] published in different articles. Table S2. Association of cKGs with other disease risks. [file 12920_2023_1488_MOESM1_ESM.docx]

**Bioinformatics screening of colorectal-cancer causing molecular signatures through gene expression profiles to discover therapeutic targets and candidate agents**

**[Supplementary File]**

| **Table S1: Different lists of hub-genes [HubGs] for colorectal cancer [CRC] published in different articles** | | | | | |  |
| --- | --- | --- | --- | --- | --- | --- |
| **Reference** | **Lists of HubGs** | | | | **Suggested drugs** |  |
| Dong et al., 2019[1] | *MYC, KLK6* | | | | Nadroparin, benzamidine |  |
| Rahman et al., 2020[2] | *JUN, MYC, FOS, EGR1, LEF1, CDC42, CTGF, ADAM10, CYR61, FOXA1, UBE2I* | | | |  |  |
| Xu et al., 2020[3] | *SST, PYY, CXCL1, CXCL8, CXCL3,*  *ZG16, AQP8, CLCA4, MS4A12, GUCA2A* | | | |  |  |
| Zheng et al., 2020[4] | *COL1A2, THBS2, TIMP1, CXCL8* | | | | Ellipticine, Ursodeoxycholic acid, Methylprednisolone, Vorinostat, Piperlongumine, Heliotrine, DL-thiorphan, Novobiocin, Doxylamine, Asiaticoside, Methocarbamol |  |
| Zhang et al., 2020[6] | *CSF2, CXCL8, IL6R, CXCL10* | | | | cetuximab, oxaliplatin, bevacizumab, temozolomide, and interferon alfa-2b |  |
| Rahman et al., 2019[7] | *ADNP, CCND1, CD44, CDK4, CEBPB, CENPA, CENPH, CENPN, MYC, RFC2* | | | | Gefitinib, Hydrocortisone, Irinotecan, Letrozole, Lidocaine, Methotrexate, Sirolimus, Tamoxifen |  |
| J. Chen et al., 2019[8] | *AURKA, CCNB1, CCNF, EXO1* | | | | DL-thiorphan, repaglinide, MS–275, quinostatin, 1,4-chrysenequinone, triflusal, trazodone, piperidolate, latamoxef, ronidazole, pargyline, gliclazide, tyloxapol, acepromazine, methylergometrine |  |
| Yu et al., 2019[9] | *TOP2A, MAD2L1, CDC6, CHEK1* | | | |  |  |
| Yang et al., 2020[10] | *CXCL1, CXCL8, IL1B, PTGS2* | | | |  |  |
| Liang, Li and Zhao, 2016[11] | *GNG2, AGT, SAA1, ADCY5, LPAR1, NMU, IL8, CXCL12, GNAI1, and CCR2* | | | |  |  |
| Dai et al., 2020[12] | *TIMP1, SLC4A4, AKR1B10 and ABCE1* | | | |  |  |
| Guo et al., 2017[13] | *CDK1, CCNB1, CENPE, KIF20A, CXCL12, DLGAP5, CCNA2, ITGA2, MAD2L1, NMU* | | | |  |  |
| Ding, Duan and Luo, 2020[14] | *CDK1, CCNA2, TOP2A, PLK1, MAD2L1, AURKA, BUB1B, UBE2C, TPX2, RRM2, KIF11, NCAPG, MELK, NUSAP1, MCM4, RFC4, PTTG1, CHEK1, CEP55, DTL* | | | |  |  |
| Yuan et al., 2020[15] | *HCLS1, EVI2B, and CD48* | | | |  |  |
| Zhao et al., 2019[16] | *MYC, CXCR1, TOP2A, CXCL12, SST, TIMP1, SPP1, PPBP, CDK1, THBS1, CXCL1, PYY, LPAR1, BMP2 and MMP3* | | | |  |  |
| Tang et al., 2020[17] | *SPARC, COL1A2, MMP9, COL11A1, COL3A1, CXCL12 and THBS2* | | | |  |  |
| Hozhabri et al., 2021[18] | *MYC, CXCL1, CD44, MMP1, and CXCL12* | | | |  |  |
| Berg et al., 2017[19] | *MYC and ERBB2* | | | |  |  |
| Qi et al., 2019[20] | *CDK1, FBL, CIRH1A, KIF2C, TOP2A, KNG1, LPAR1, GNG7, CCL5, and GNAI1* | | | |  |  |
| Z. Chen et al., 2019[21] | *TOP2A, PAICS, CDK1, CKS2, CKAP2, CEP55, VEGFA, PHLPP2, RRM2, NEK2* | | | |  |  |
| Mastrogamvraki and Zaravinos, 2020[22] | *MYC, PML, CDKs, CSNK2A1, and MAPK* | | | |  |  |
| Liu et al., 2020[23] | *AXIN2, CXCL1, ITLN1, CPT2, CLDN23, TIMP1 and LZTS3* | | | |  |  |
| Pirim, 2020[24] | *AURKA, CDK1, MYC, CDH1, CCNB1, CDC20, UBE2C, PLK1, KIF11, and CCNA2* | | | |  |  |
| Cui et al., 2017[25] | *DEPTOR, AURKA, CCND1, BCAS1, NEDD9 and MAP2K2* | | | |  |  |
| Zhu et al., 2019[26] | *E2F2, SKP2, MYC, CDKN1A and CDKN2B* | | | |  |  |
| Peng et al., 2018[27] | *HADHB, NDUFS3, TAF1, MYC, HNFF4A, and MAX* | | | |  |  |
| Liu et al., 2021[28] | *TIMP1, SPARCL1, MYL9, TPM2, and CNN1* | | | |  |  |
| Asghari et al., 2018[29] | *CDK1, UBC, ESR1 and ATXN1* | | | |  |  |
| Lin et al., 2020[30] | *VEGFA, CASP3, MYC, CYP1Y1, and NFKB1* | | | | Quercetin, Wogonin, Luteolin, Eburicoic acid, Kaempferol, 5,7,4′-Trihydroxy-8-methoxyflavone, Quercetin der., Formononetin, Jaranol, Dehydrotumulosic acid, Pachymic acid, Carthamidin, Pinocembrin, Baicalein, Spinasterol |  |
| Kasap et al., 2016[31] | *NEK6, AURKA, AURKB, and PAK1* | | | |  |  |
| Hameed et al., 2021[32] | *CXCL12, CXCL8, AGT, GNB1, GNG4, and CXCL1* | | | |  |  |
| Yang et al., 2021[33] | *CXCL1, HCAR3, CXCL6, CXCL8, CXCL2, CXCL5, PPY, SST, INSL5, and NPY1R* | | | |  |  |
| Zhang et al., 2018[33] | *MCM2, RNASEH2A, and TOP2A* | | | |  |  |
| Yun et al., 2022[34] | *TIMP1* | | | | formaldehyde, paclitaxel/eribulin, erlotinib/dimethyl sulfoxide, glucocorticoid/dexamethasone, antagonist, trichostatin A, rosiglitazone, inhibitor, retinoic acid, and cisplatin. |  |
| Wang et al., 2022[35] | *IL1RN and PRRX1* | | | |  |  |
| Sharma et al., 2022, [36] | *MT2A, CCNB1, DLGAP5, CCNA2, CXCL2, and RACGAP1* | | | |  |  |
| Common in 11 papers: *MYC* | | | | | |  |
| Common in 7 papers: *CDK1, CXCL1* | | | | | |  |
| Common in 6 papers: *CXCL8, CXCL12, TOP2A, TIMP1* | | | | | |  |
| Common in 5 papers: *AURKA* | | | | | |  |
| Common in 4 papers: *CCNB1, CCNA2* | | | | | |  |
| **Table S2: Association of cKGs with different diseases** | | | | | | |
| Term | | Overlap | P-value | Genes | | |
| Malignant tumor of colon | | 8/2001 | 1.24E-06 | *CEMIP, CXCL8, MMP7, CA4, GUCA2B, MS4A12, CLDN1, GUCA2A* | | |
| Colonic Neoplasms | | 6/778 | 1.33E-06 | *CEMIP, CXCL8, MMP7, CA4, CLDN1, GUCA2A* | | |
| Incontinence | | 2/7 | 5.77E-06 | *CXCL8, CLDN1* | | |
| Adenomatous Polyps | | 3/69 | 6.36E-06 | *CXCL8, MMP7, ADH1C* | | |
| Adenocarcinoma | | 7/1712 | 8.06E-06 | *CEMIP, CXCL8, MMP7, ADH1C, GUCA2B, CLDN1, GUCA2A* | | |
| Adenoma of large intestine | | 4/258 | 8.31E-06 | *CEMIP, CXCL8, MMP7, ADH1C* | | |
| Colorectal Neoplasms | | 6/1073 | 8.61E-06 | *CEMIP, CXCL8, MMP7, ADH1C, CLDN1, GUCA2A* | | |
| Adenocarcinoma of colon | | 4/267 | 9.52E-06 | *CXCL8, MMP7, CLDN1, GUCA2A* | | |
| Colon Carcinoma | | 7/2091 | 3.05E-05 | *CEMIP, CXCL8, MMP7, GUCA2B, MS4A12, CLDN1, GUCA2A* | | |
| Mouth Neoplasms | | 3/132 | 4.46E-05 | *CXCL8, MMP7, ADH1C* | | |
| Stage III Colon Cancer AJCC v7 | | 2/23 | 6.91E-05 | *CEMIP, CXCL8* | | |
| Healing ulcer | | 2/24 | 7.54E-05 | *CXCL8, MMP7* | | |
| Stage III Colon Cancer | | 2/24 | 7.54E-05 | *CEMIP, CXCL8* | | |
| Intestinal Neoplasms | | 3/158 | 7.62E-05 | *CXCL8, MMP7, GUCA2A* | | |
| Lip and Oral Cavity Carcinoma | | 4/473 | 8.93E-05 | *CXCL8, MMP7, ADH1C, CLDN1* | | |
| Follicular thyroid carcinoma | | 3/167 | 8.98E-05 | *CXCL8, MMP7, CLDN1* | | |
| Irritable Bowel Syndrome | | 3/167 | 8.98E-05 | *CXCL8, GUCA2B, CLDN1* | | |
| Adenoma | | 5/1000 | 1.11E-04 | *CEMIP, CXCL8, MMP7, ADH1C, CLDN1* | | |
| Metastatic Neoplasm | | 4/544 | 1.53E-04 | *CEMIP, CXCL8, MMP7, CLDN1* | | |

**References:**

1. Dong S, Ding Z, Zhang H, Chen Q. Identification of Prognostic Biomarkers and Drugs Targeting Them in Colon Adenocarcinoma: A Bioinformatic Analysis. Integr Cancer Ther. 2019;18.

2. Rahman F, Mahmud P, Karim R, Hossain T, Islam F. Determination of novel biomarkers and pathways shared by colorectal cancer and endometrial cancer via comprehensive bioinformatics analysis. Informatics Med Unlocked. 2020;20.

3. Xu H, Ma Y, Zhang J, Gu J, Jing X, Lu S, et al. Identification and Verification of Core Genes in Colorectal Cancer. Biomed Res Int. 2020;2020.

4. Zheng Z, Xie J, Xiong L, Gao M, Qin L, Dai C, et al. Identification of candidate biomarkers and therapeutic drugs of colorectal cancer by integrated bioinformatics analysis. Med Oncol. 2020;37:1–11.

5. Zhang J, Zhang H, Li F, Song Z, Li Y, Zhao T. Identification of intestinal flora-related key genes and therapeutic drugs in colorectal cancer. BMC Med Genomics. 2020;13.

6. Rahman MR, Islam T, Gov E, Turanli B, Gulfidan G, Shahjaman M, et al. Identification of prognostic biomarker signatures and candidate drugs in colorectal cancer: Insights from systems biology analysis. Med. 2019;55.

7. Chen J, Wang Z, Shen X, Cui X, Guo Y. Identification of novel biomarkers and small molecule drugs in human colorectal cancer by microarray and bioinformatics analysis. Mol Genet Genomic Med. 2019;7.

8. Yu C, Chen F, Jiang J, Zhang H, Zhou M. Screening key genes and signaling pathways in colorectal cancer by integrated bioinformatics analysis. Mol Med Rep. 2019;20:1259–69.

9. Yang J, Gao S, Qiu M, Kan S. Integrated Analysis of Gene Expression and Metabolite Data Reveals Candidate Molecular Markers in Colorectal Carcinoma. Cancer Biother Radiopharm. 2020. https://doi.org/10.1089/cbr.2020.3980.

10. Liang B, Li C, Zhao J. Identification of key pathways and genes in colorectal cancer using bioinformatics analysis. Med Oncol. 2016;33.

11. Dai GP, Wang LP, Wen YQ, Ren XQ, Zuo SG. Identification of key genes for predicting colorectal cancer prognosis by integrated bioinformatics analysis. Oncol Lett. 2020;19:388–98.

12. Guo Y, Bao Y, Ma M, Yang W. Identification of key candidate genes and pathways in colorectal cancer by integrated bioinformatical analysis. Int J Mol Sci. 2017;18.

13. Ding X, Duan H, Luo H. Identification of Core Gene Expression Signature and Key Pathways in Colorectal Cancer. Front Genet. 2020;11.

14. Yuan Y, Chen J, Wang J, Xu M, Zhang Y, Sun P, et al. Identification Hub Genes in Colorectal Cancer by Integrating Weighted Gene Co-Expression Network Analysis and Clinical Validation in vivo and vitro. Front Oncol. 2020;10.

15. Zhao Z, Fan X, Yang L, Song J, Fang S, Tu J, et al. The identification of a common different gene expression signature in patients with colorectal cancer. Math Biosci Eng. 2019;16:2942–58.

16. Tang L, Lei YY, Liu YJ, Tang B, Yang SM. The expression of seven key genes can predict distant metastasis of colorectal cancer to the liver or lung. J Dig Dis. 2020;21:639–49.

17. Hozhabri H, Lashkari A, Razavi SM, Mohammadian A. Integration of gene expression data identifies key genes and pathways in colorectal cancer. Med Oncol. 2021;38.

18. Berg KCG, Eide PW, Eilertsen IA, Johannessen B, Bruun J, Danielsen SA, et al. Multi-omics of 34 colorectal cancer cell lines - a resource for biomedical studies. Mol Cancer. 2017;16.

19. Qi Y, Qi H, Liu Z, He P, Li B. Bioinformatics Analysis of Key Genes and Pathways in Colorectal Cancer. J Comput Biol. 2019;26:364–75.

20. Chen Z, Lin Y, Gao J, Lin S, Zheng Y, Liu Y, et al. Identification of key candidate genes for colorectal cancer by bioinformatics analysis. Oncol Lett. 2019;18:6583–93.

21. Mastrogamvraki N, Zaravinos A. Signatures of co-deregulated genes and their transcriptional regulators in colorectal cancer. npj Syst Biol Appl. 2020;6.

22. Liu X, Bing Z, Wu J, Zhang J, Zhou W, Ni M, et al. Integrative gene expression profiling analysis to investigate potential prognostic biomarkers for colorectal cancer. Med Sci Monit. 2020;26.

23. Pirim D. Integrative analyses of molecular pathways and key candidate biomarkers associated with colorectal cancer. Cancer Biomarkers. 2020;27:555–68.

24. Cui X, Shen K, Xie Z, Liu T, Zhang H. Identification of key genes in colorectal cancer using random walk with restart. Mol Med Rep. 2017;15:867–72.

25. Zhu H, Ji Y, Li W, Wu M. Identification of key pathways and genes in colorectal cancer to predict the prognosis based on mRNA interaction network. Oncol Lett. 2019;18:3778–86.

26. Peng WF, Bai F, Shao K, Shen LS, Li HH, Huang S. The key genes underlying pathophysiology association between the type 2-diabetic and colorectal cancer. J Cell Physiol. 2018;233:8551–7.

27. Liu S, Zeng F, Fan G, Dong Q. Identification of Hub Genes and Construction of a Transcriptional Regulatory Network Associated With Tumor Recurrence in Colorectal Cancer by Weighted Gene Co-expression Network Analysis. Front Genet. 2021;12.

28. Asghari M, Abazari MF, Bokharaei H, Aleagha MN, Poortahmasebi V, Askari H, et al. Key genes and regulatory networks involved in the initiation, progression and invasion of colorectal cancer. Futur Sci OA. 2018;4.

29. Lin T, Liang C, Peng W, Qiu Y, Peng L. Mechanisms of core Chinese herbs against colorectal cancer: A study based on data mining and network pharmacology. Evidence-based Complement Altern Med. 2020;2020.

30. Kasap E, Gerceker E, Boyacıoglu SÖ, Yuceyar H, Yıldırm H, Ayhan S, et al. The potential role of the NEK6, AURKA, AURKB, and PAK1 genes in adenomatous colorectal polyps and colorectal adenocarcinoma. Tumor Biol. 2016;37:3071–80.

31. Hameed Y, Usman M, Liang S, Ejaz S. Novel diagnostic and prognostic biomarkers of colorectal cancer: Capable to overcome the heterogeneity-specific barrier and valid for global applications. PLoS One. 2021;16 9 September.

32. Yang X, Wei W, Tan S, Guo L, Qiao S, Yao B, et al. Identification and verification of HCAR3 and INSL5 as new potential therapeutic targets of colorectal cancer. World J Surg Oncol. 2021;19.

33. Zhang L, Yang Y, Cheng L, Cheng Y, Zhou HH, Tan ZR. Identification of Common Genes Refers to Colorectal Carcinogenesis with Paired Cancer and Noncancer Samples. Dis Markers. 2018;2018.

34. Leng X, Yang J, Liu T, Zhao C, Cao Z, Li C, et al. A bioinformatics framework to identify the biomarkers and potential drugs for the treatment of colorectal cancer. Front Genet. 2022;13.

35. Wang Q, Huang X, Zhou S, Ding Y, Wang H, Jiang W, et al. IL1RN and PRRX1 as a Prognostic Biomarker Correlated with Immune Infiltrates in Colorectal Cancer: Evidence from Bioinformatic Analysis. Int J Genomics. 2022;2022.

36. Sharma A, Yadav D, Rao P, Sinha S, Goswami D, Rawal RM, et al. Identification of potential therapeutic targets associated with diagnosis and prognosis of colorectal cancer patients based on integrated bioinformatics analysis. Comput Biol Med. 2022;146.
