# Supplementary material for: Bioinformatics screening of colorectal-cancer causing molecular signatures through gene expression profiles to discover therapeutic targets and candidate agents
Source: BMC Med Genomics. 2023 Mar 29;16:64. doi: 10.1186/s12920-023-01488-w (PMC10053149; doi:10.1186/s12920-023-01488-w)
Supplement: Supplementary file 3 — Additional file 3. Correlation plots illustrating the relationship between gene expression and immune infiltration levels across multiple types of cancers, using the bioinformatics tool TIMER 2.0. [file 12920_2023_1488_MOESM3_ESM.pdf]

Relationship of ADH1C expression with immune infiltration level in diverse cancer types (TIMER 2.0).

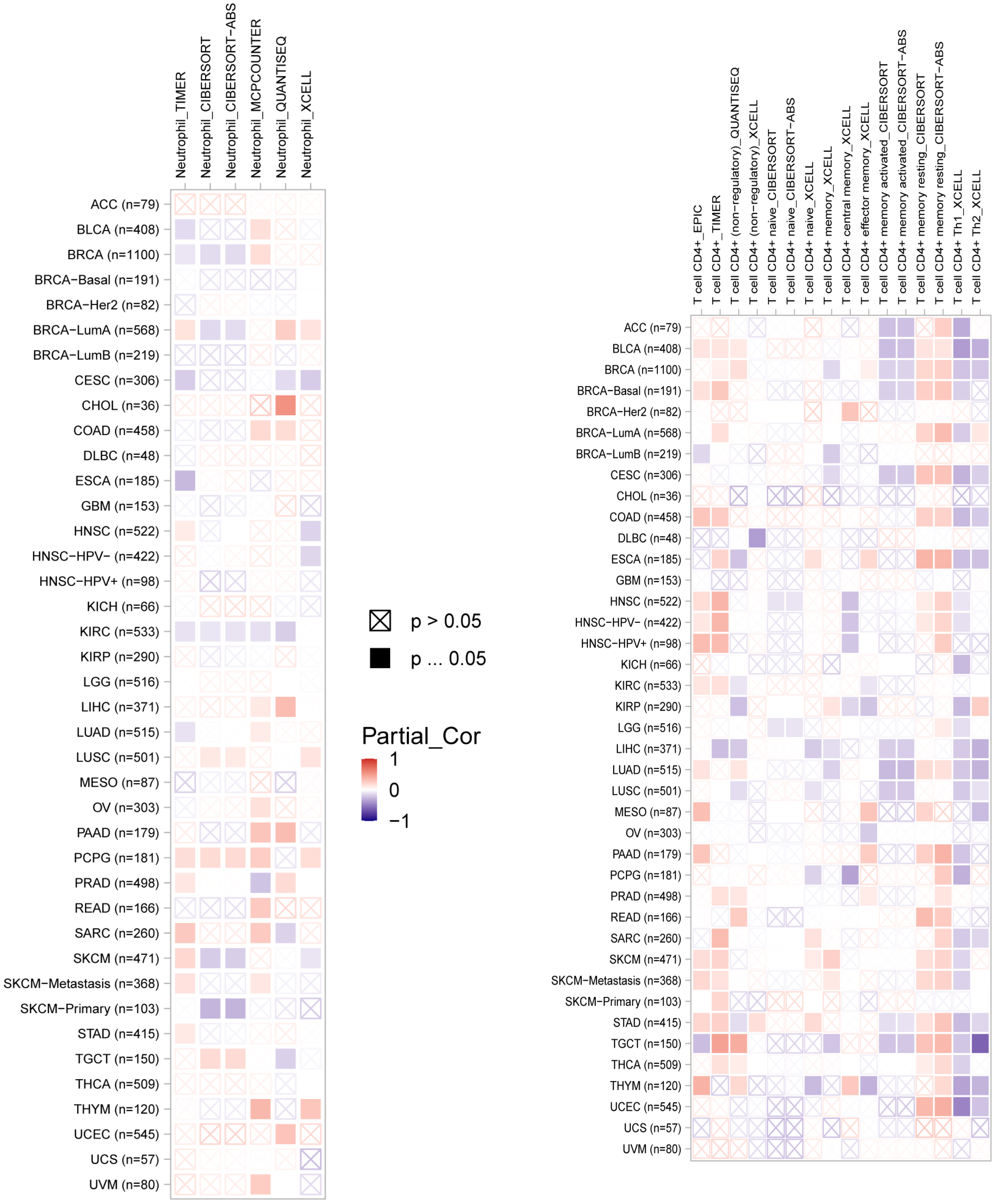

Neutrophil immune infiltration level.

T cell CD4+ immune infiltration level.

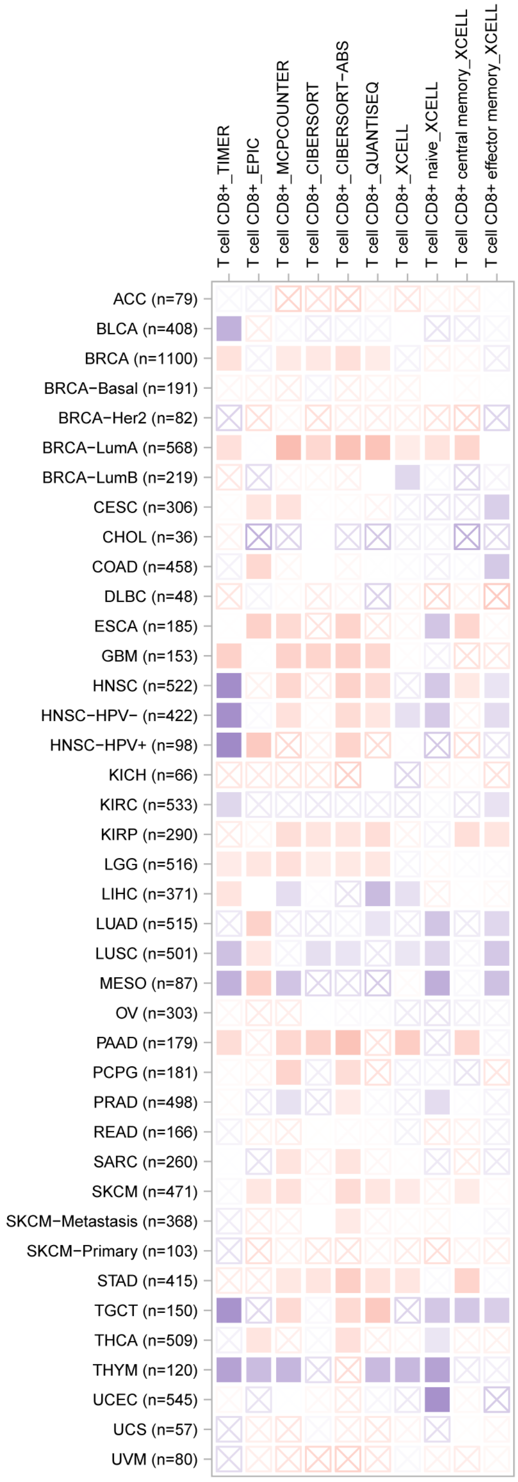

T cell CD8+ immune infiltration level.

Relationship of CA4 expression with immune infiltration level in diverse cancer types (TIMER 2.0).

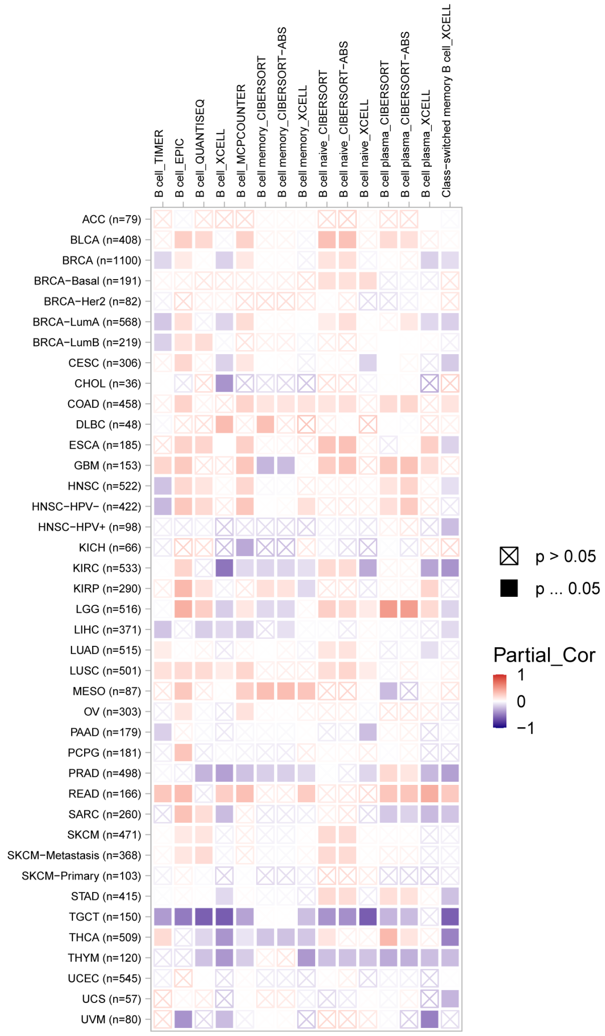

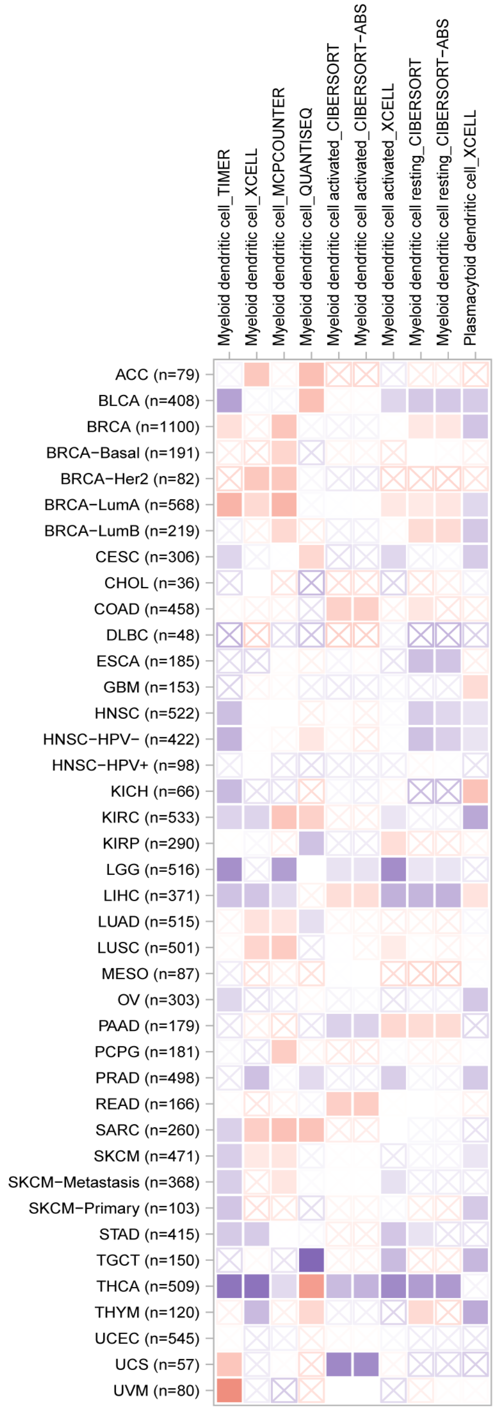

B cell immune infiltration level

DC cell immune infiltration level

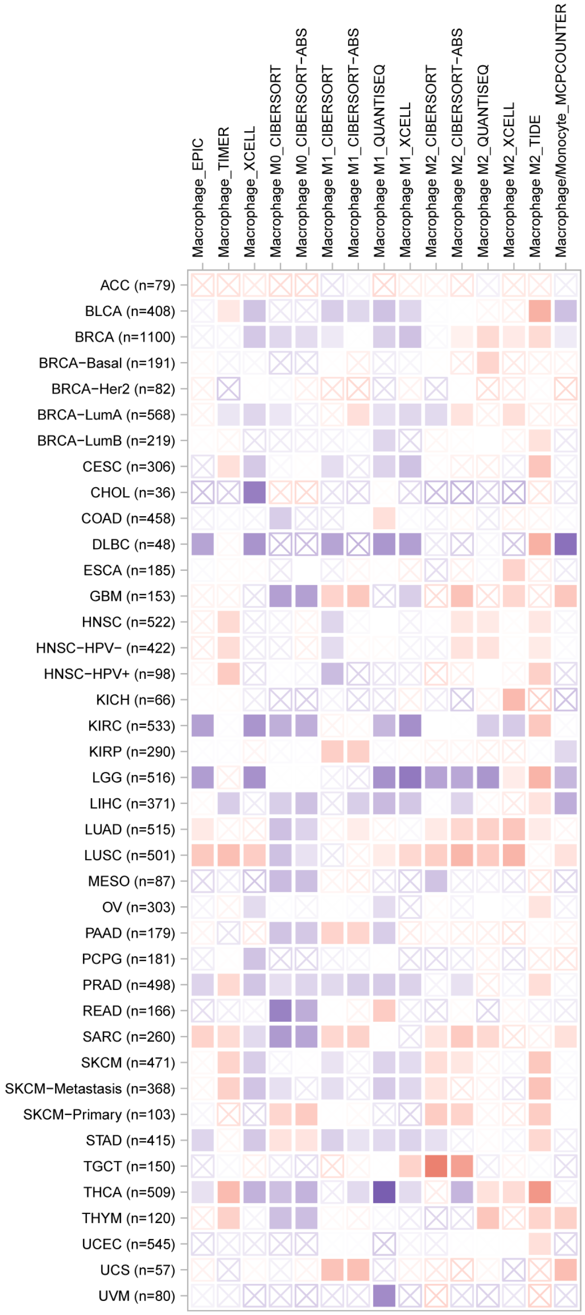

Macrophage immune infiltration level

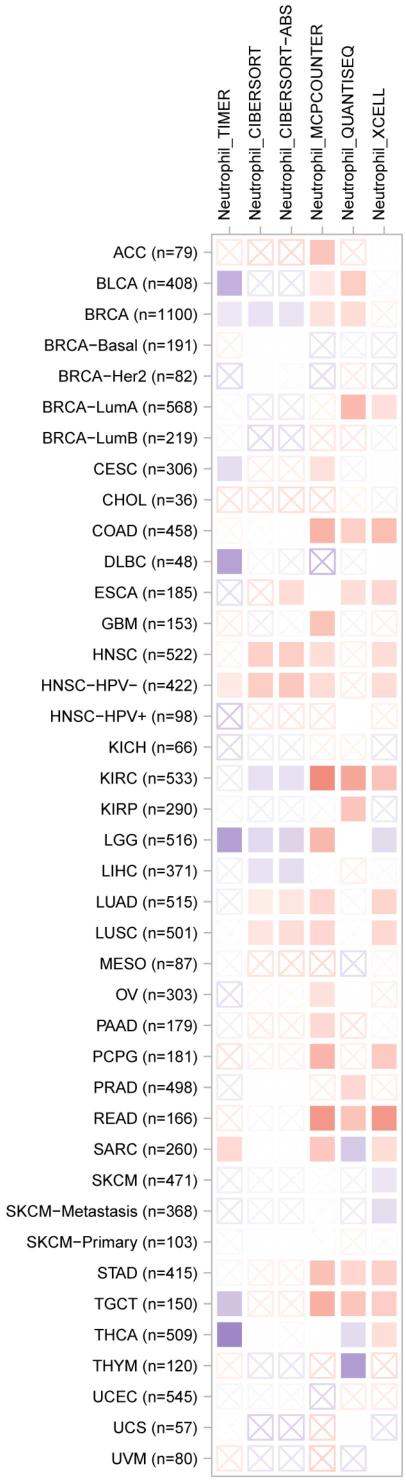

Neutrophil immune infiltration level

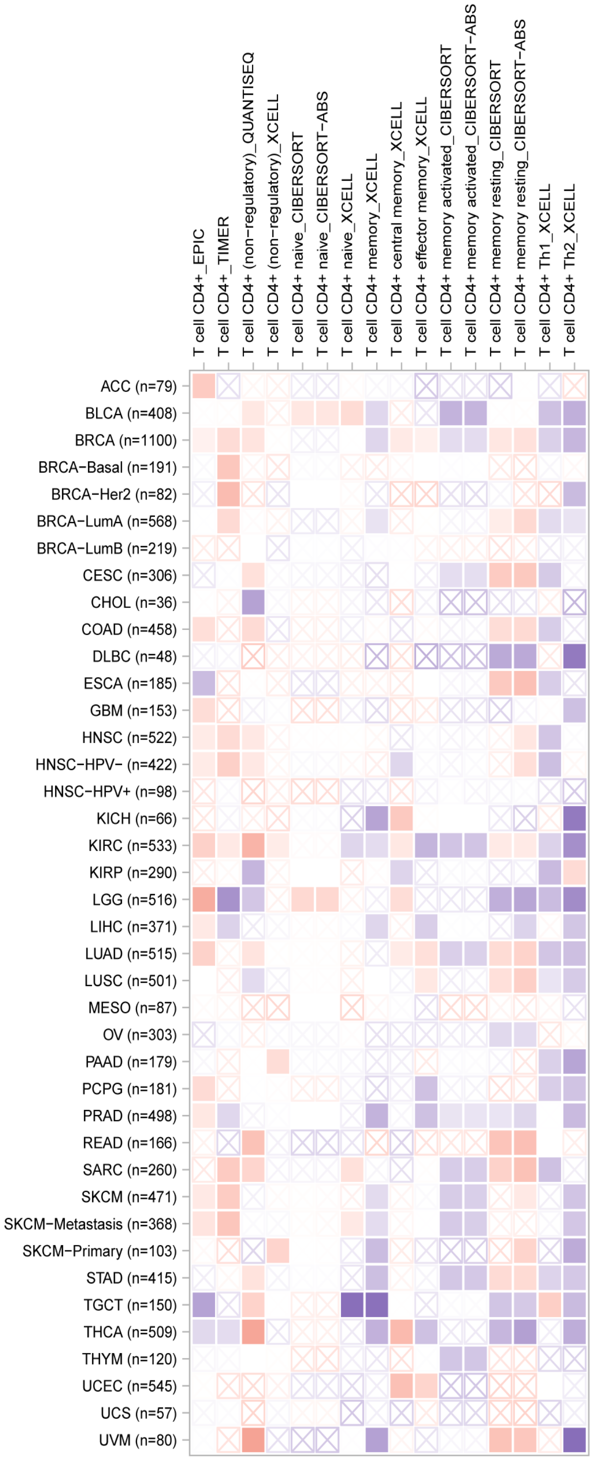

T cell CD4+ immune infiltration level

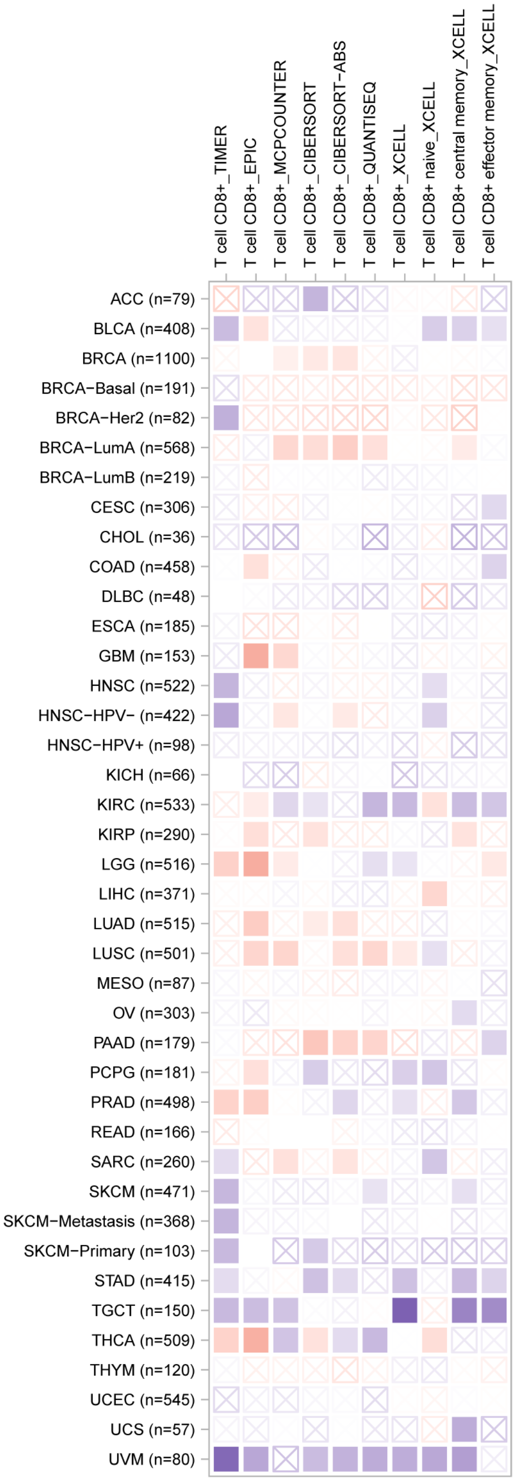

T cell CD8+ immune infiltration level

Relationship of CLCA4 expression with immune infiltration level in diverse cancer types (TIMER 2.0).

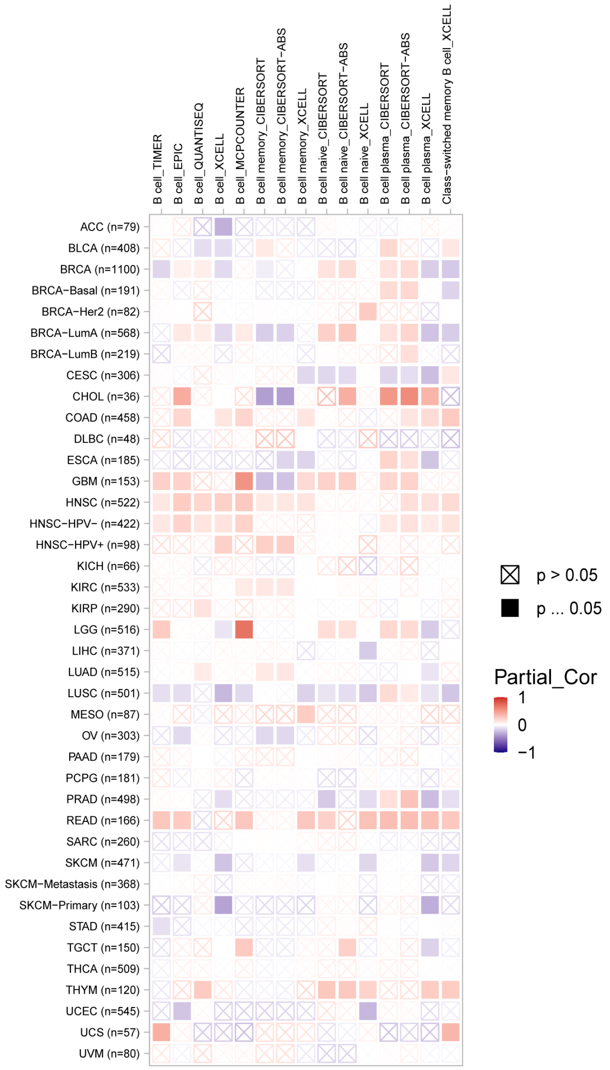

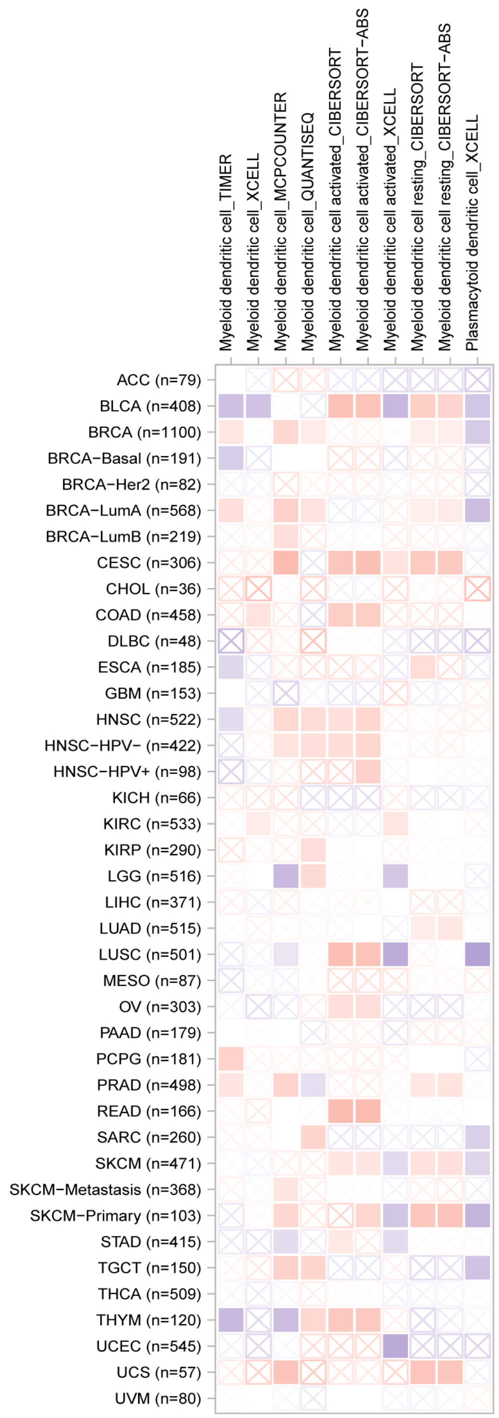

B cell immune infiltration level

DC cell immune infiltration level

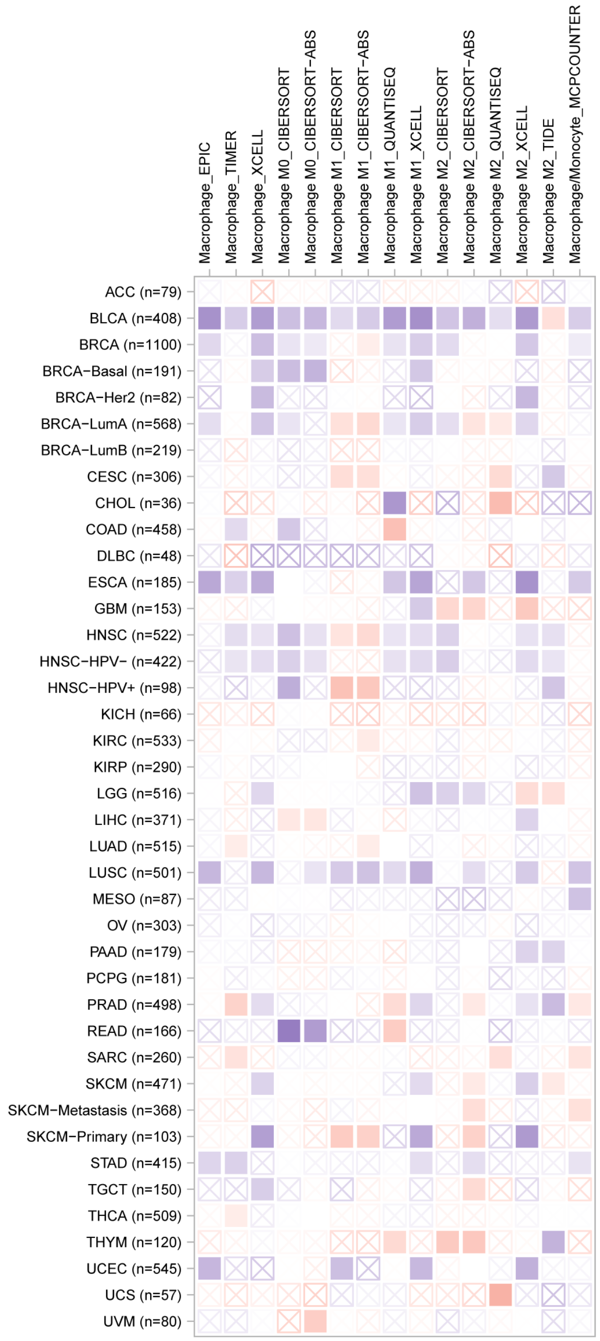

Macrophage immune infiltration level

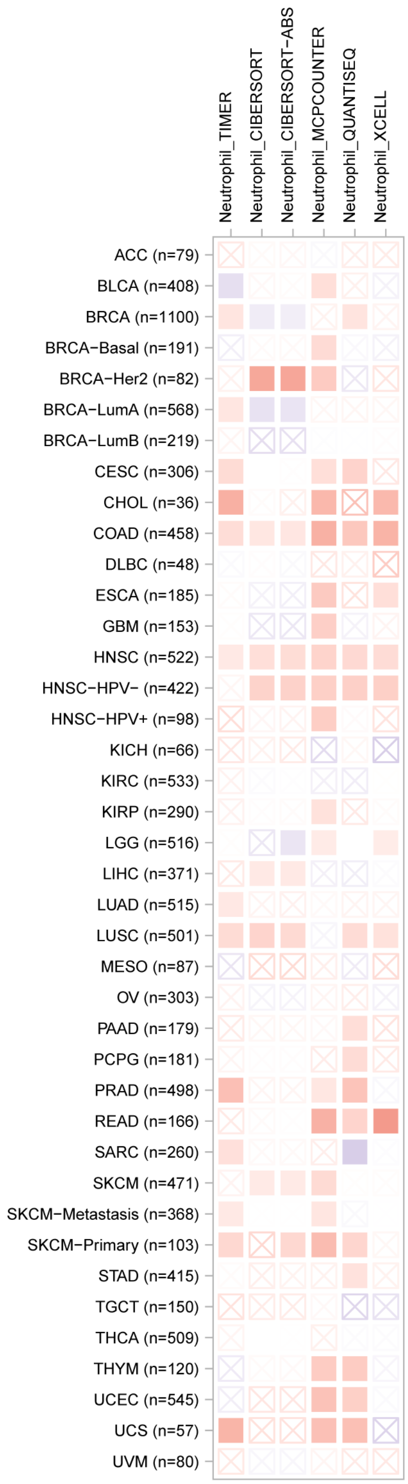

Neutrophil immune infiltration level

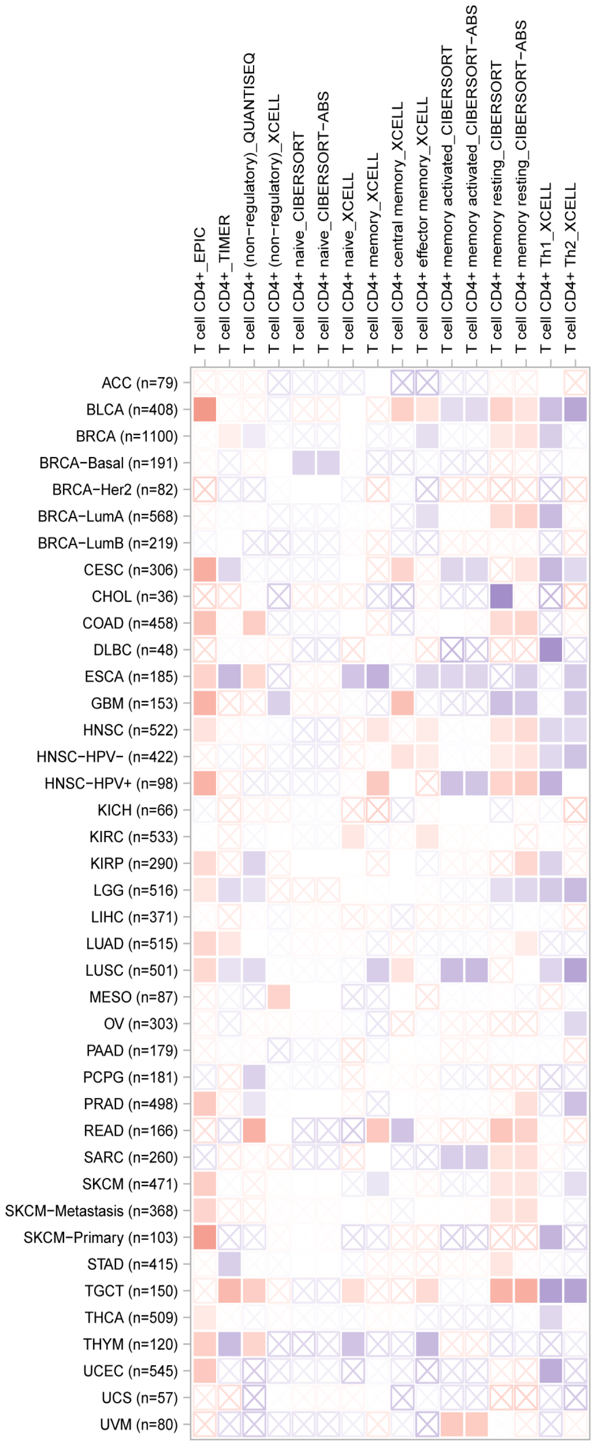

T cell CD4+ immune infiltration level

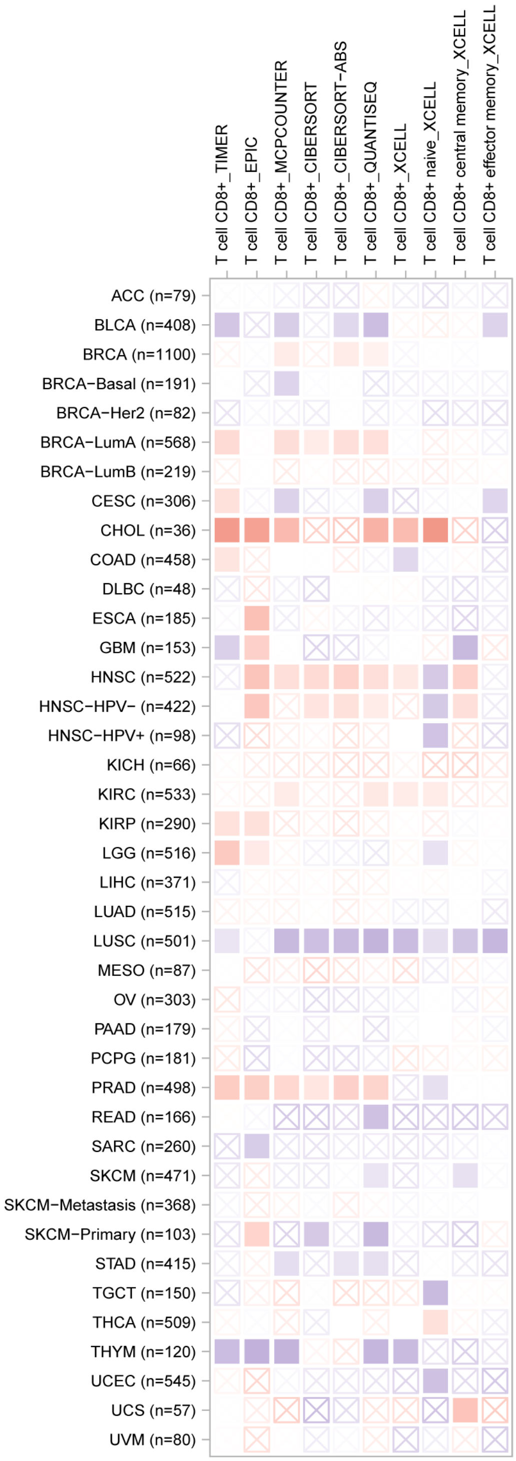

T cell CD8+ immune infiltration level

Relationship of CLDN1 expression with immune infiltration level in diverse cancer types (TIMER 2.0).

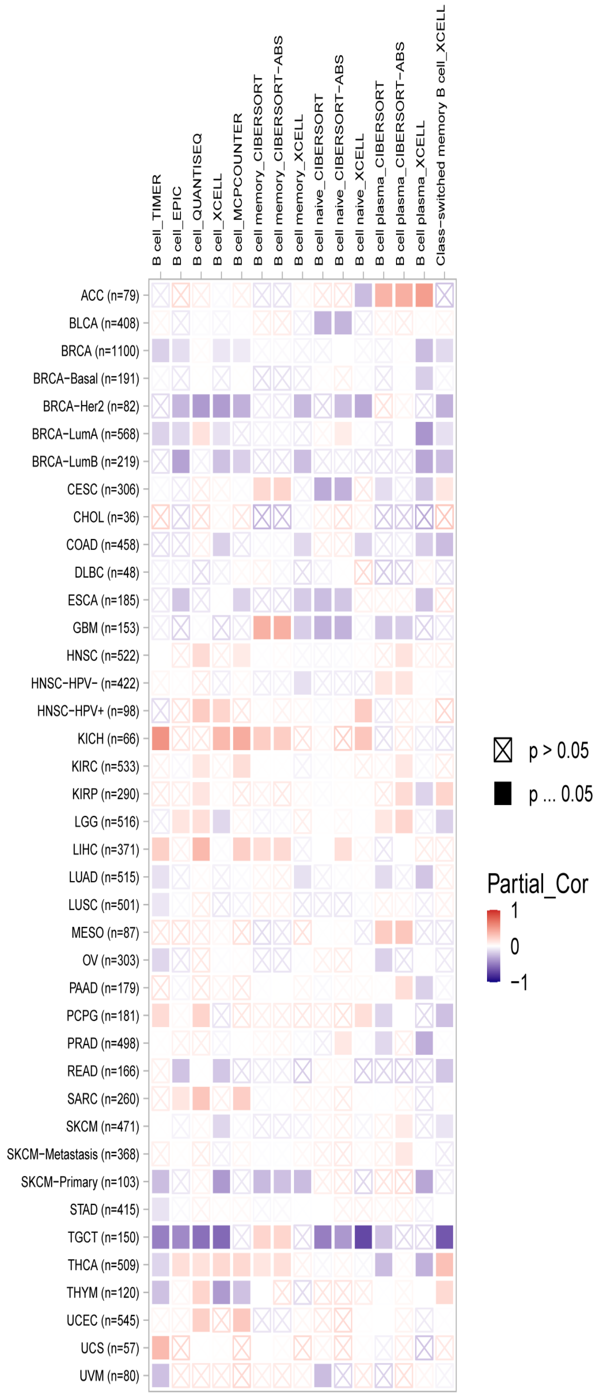

B cell immune infiltration level

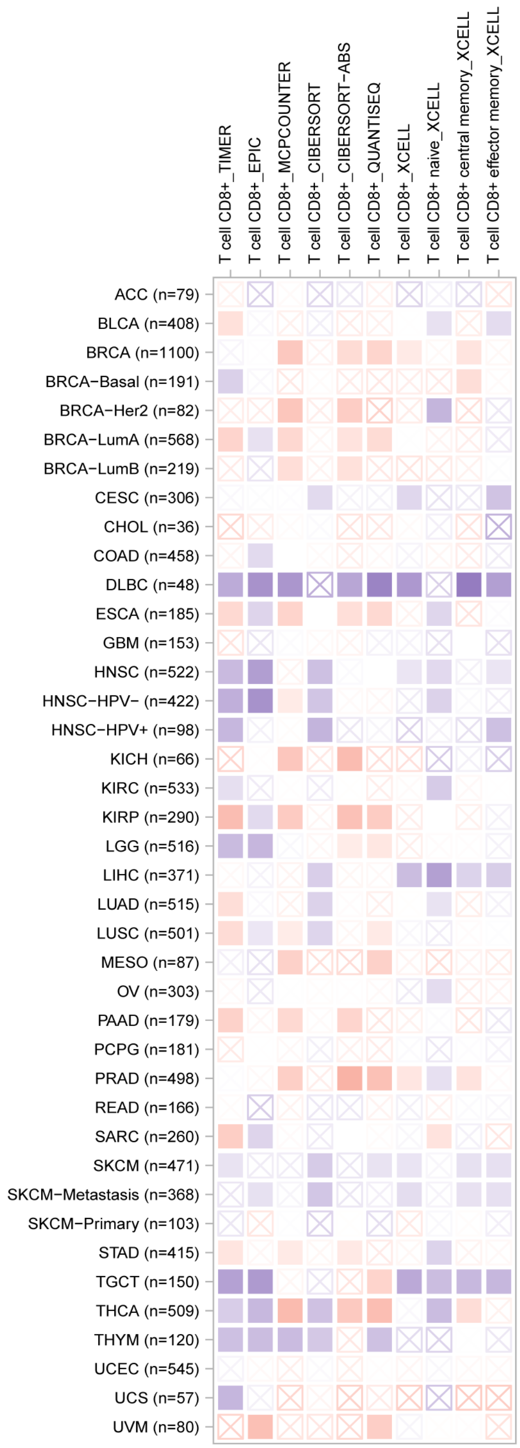

DC cell immune infiltration level

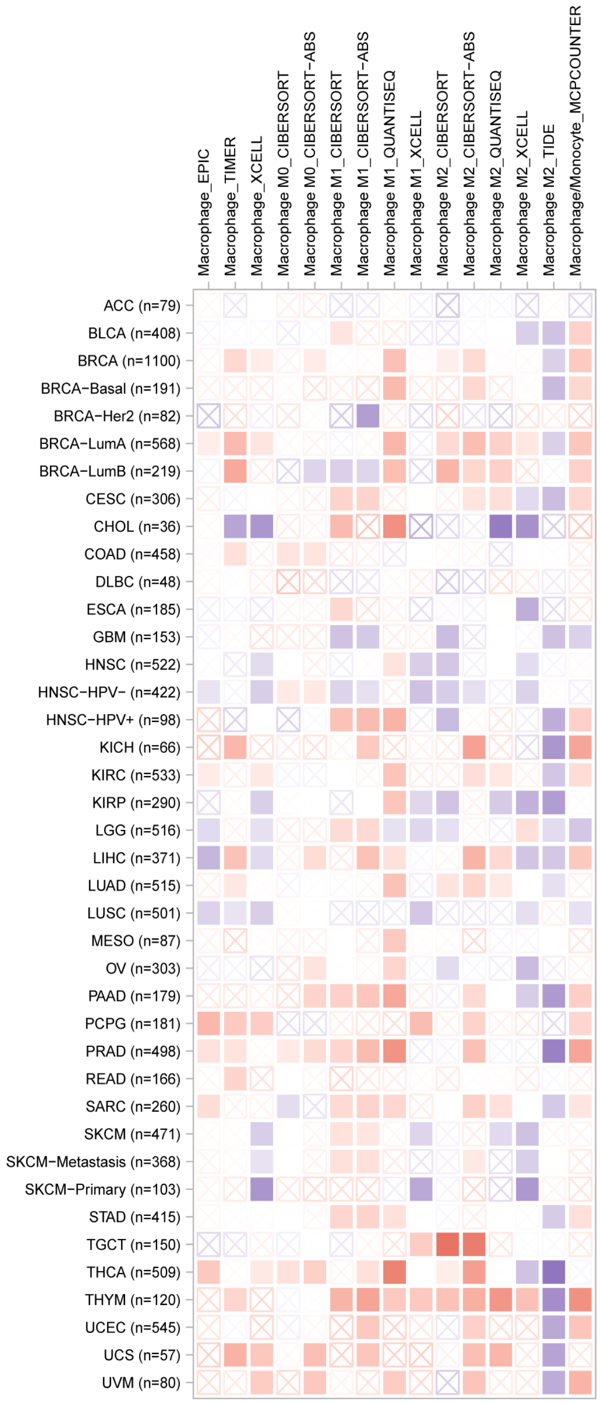

Macrophage immune infiltration level

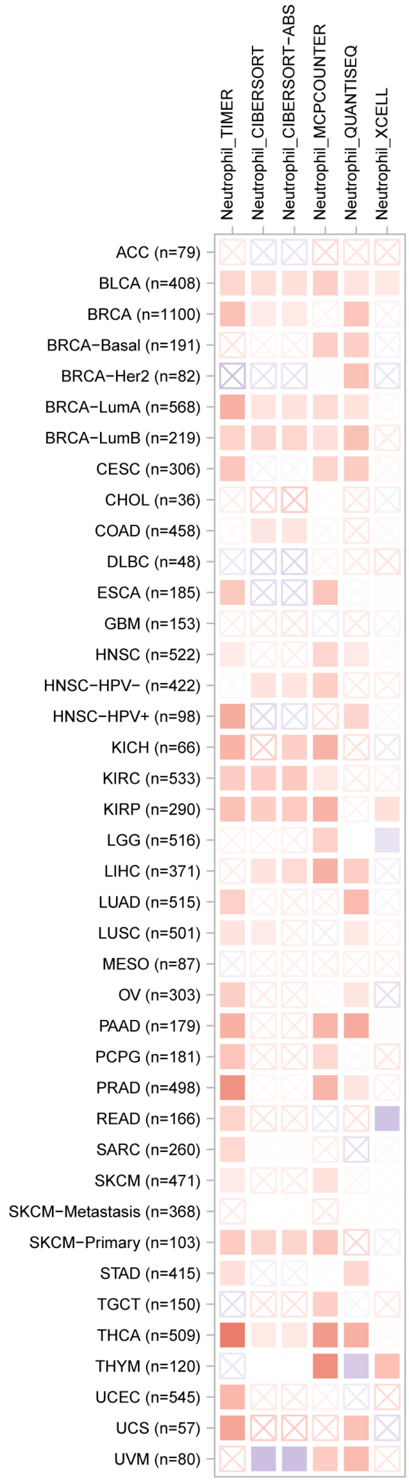

Neutrophil immune infiltration level

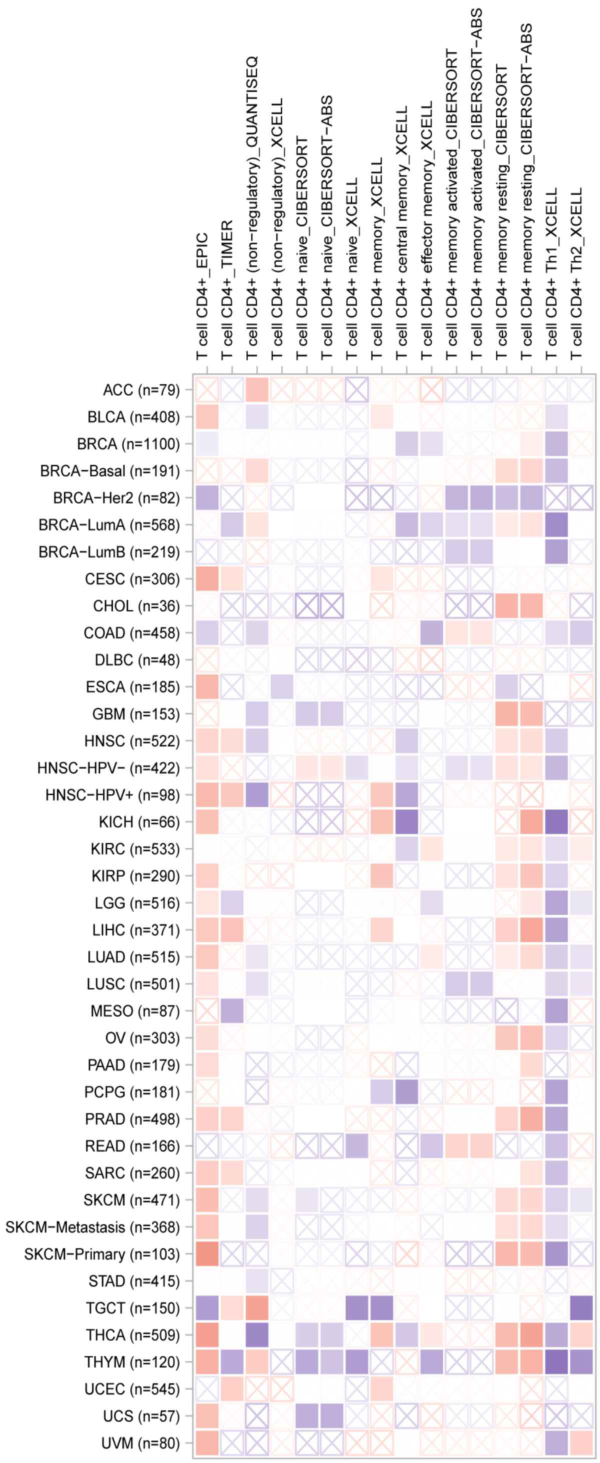

T cell CD4+ immune infiltration level

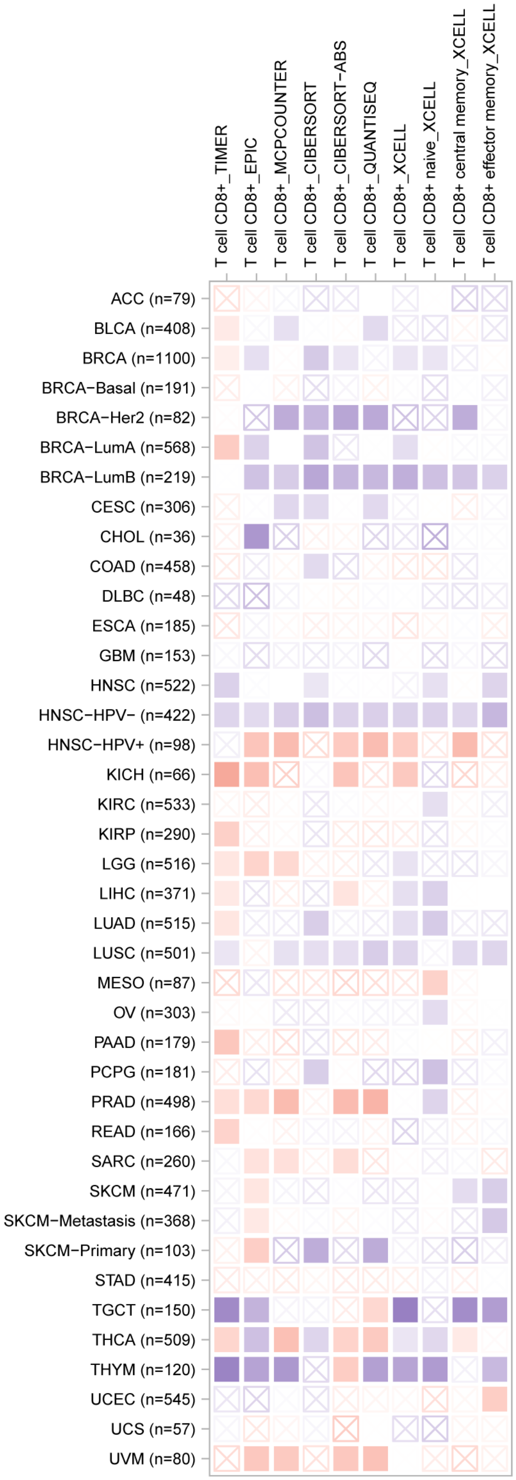

T cell CD8+ immune infiltration level

Relationship of GUCA2A expression with immune infiltration level in diverse cancer types (TIMER 2.0).

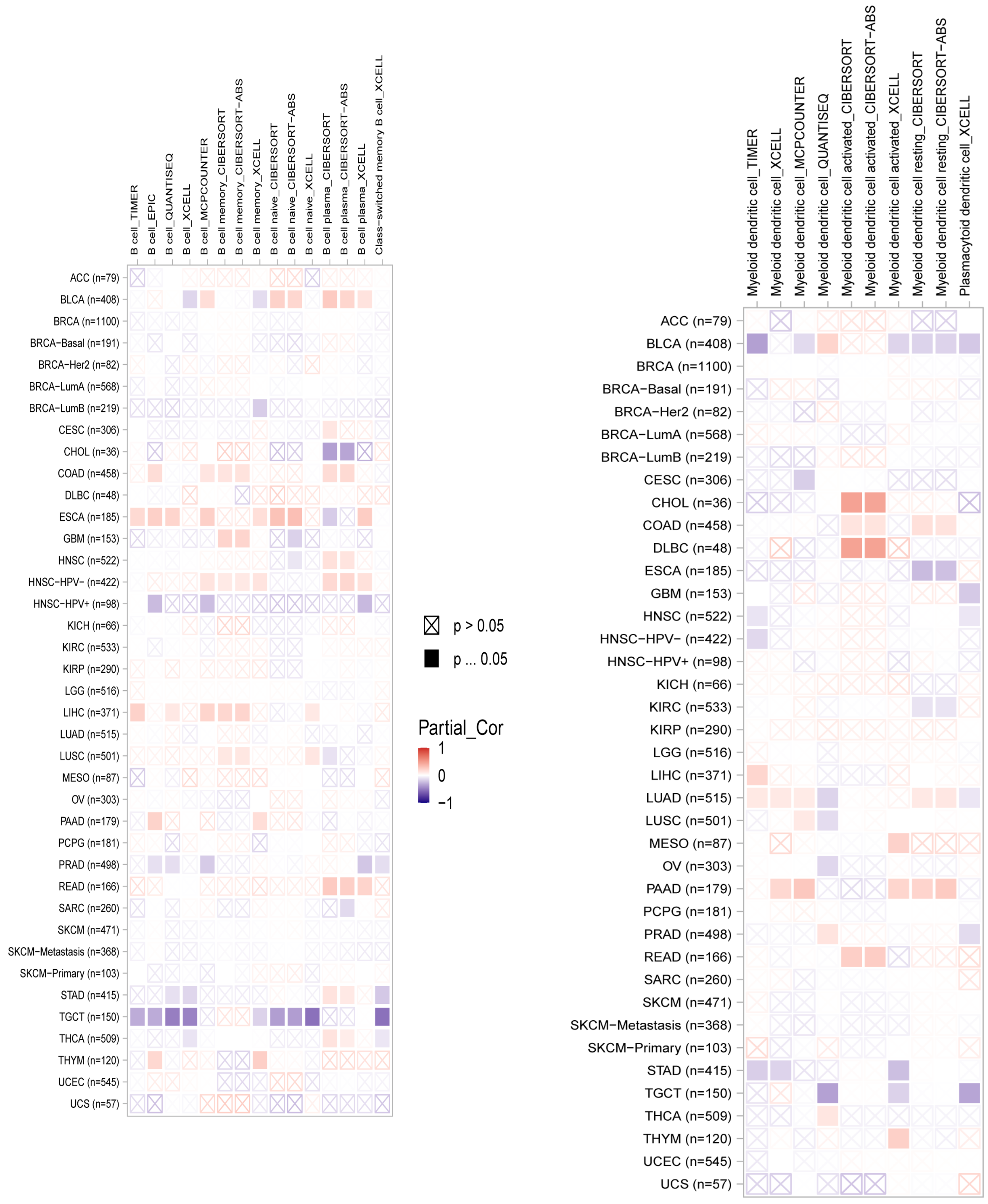

B cell immune infiltration level

DC cell immune infiltration level

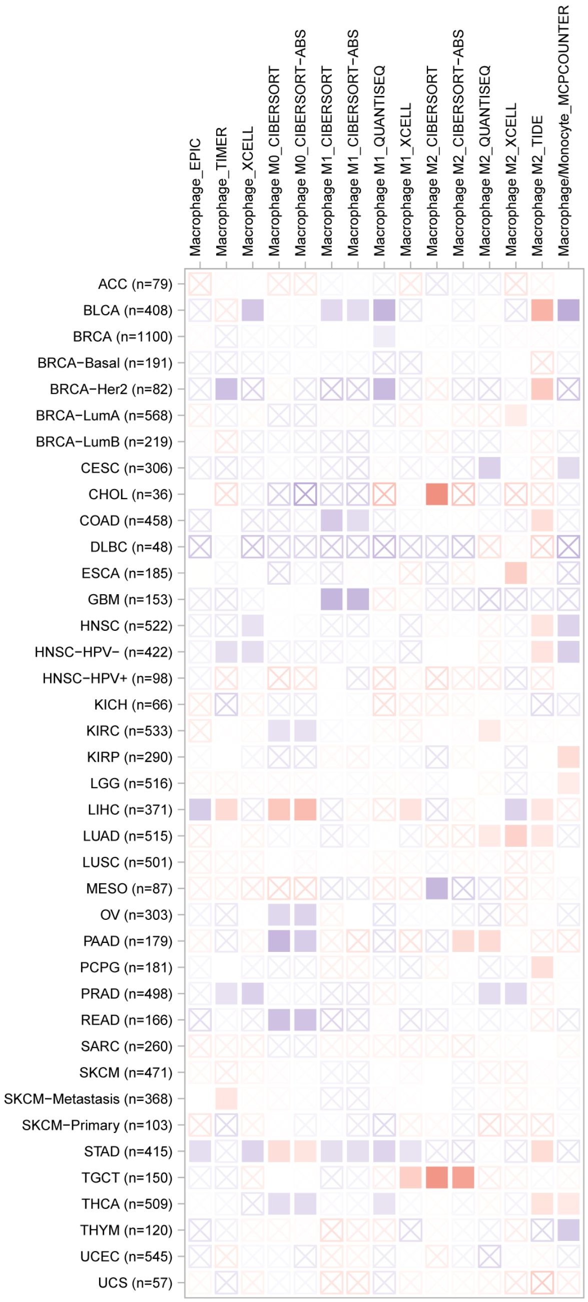

Macrophage immune infiltration level

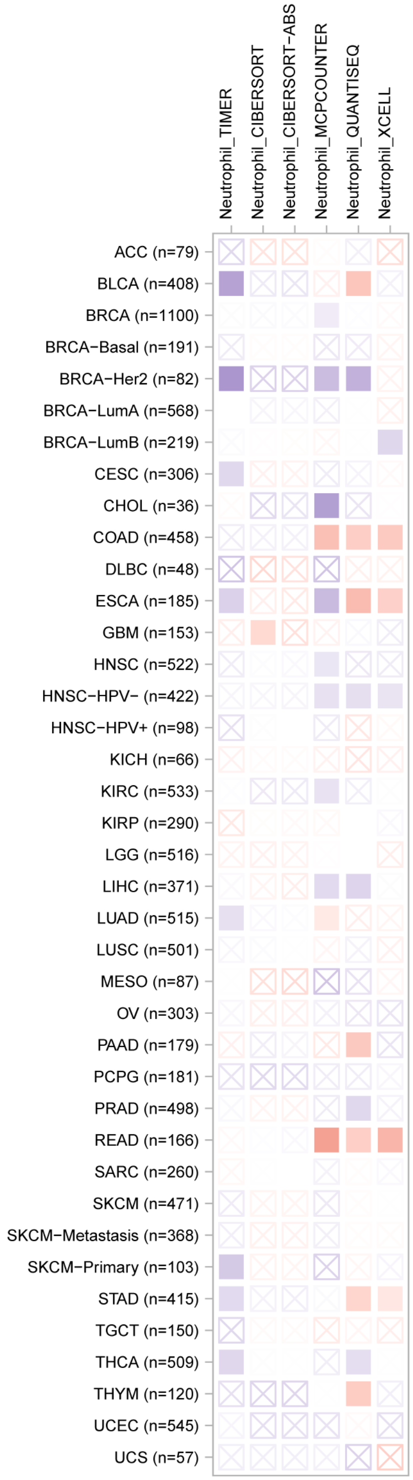

Neutrophil immune infiltration level

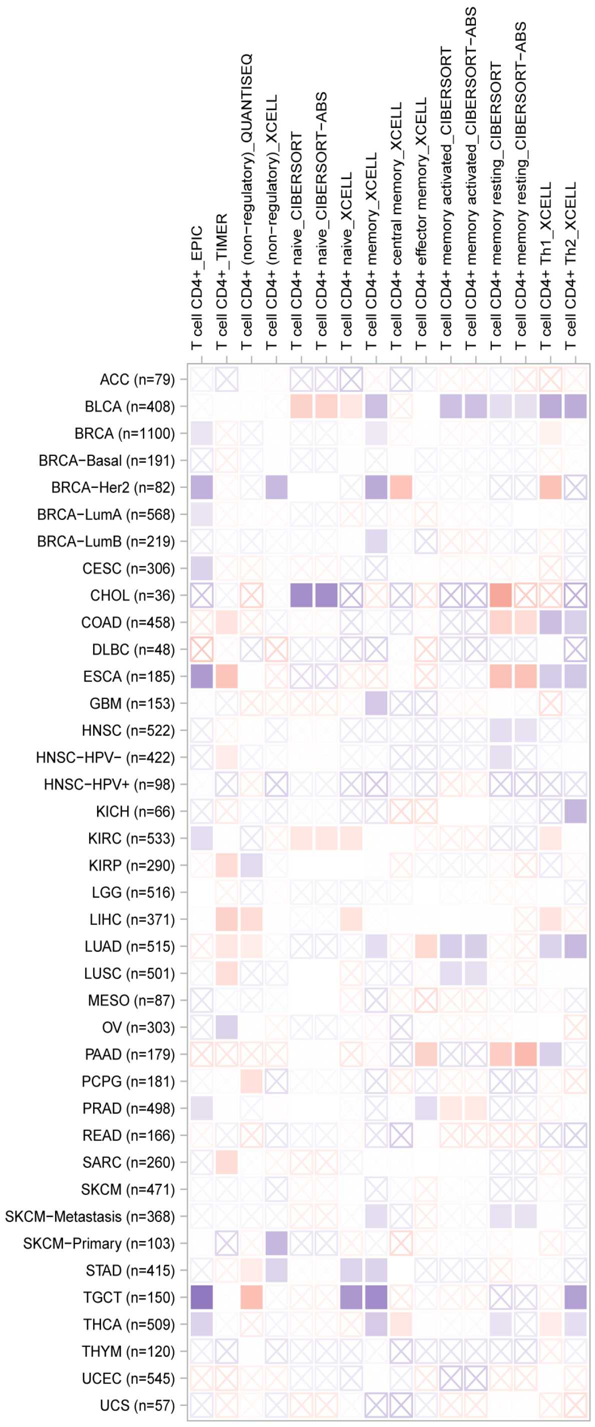

T cell CD4+ immune infiltration level

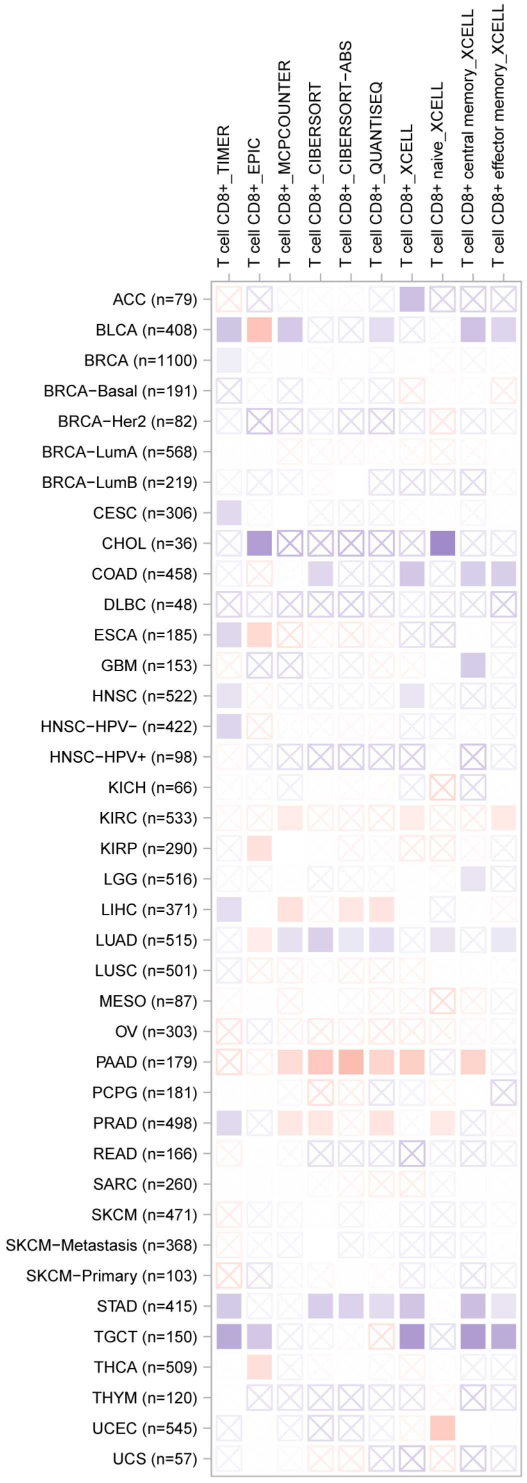

T cell CD8+ immune infiltration level

Relationship of GUCA2B expression with immune infiltration level in diverse cancer types (TIMER 2.0).

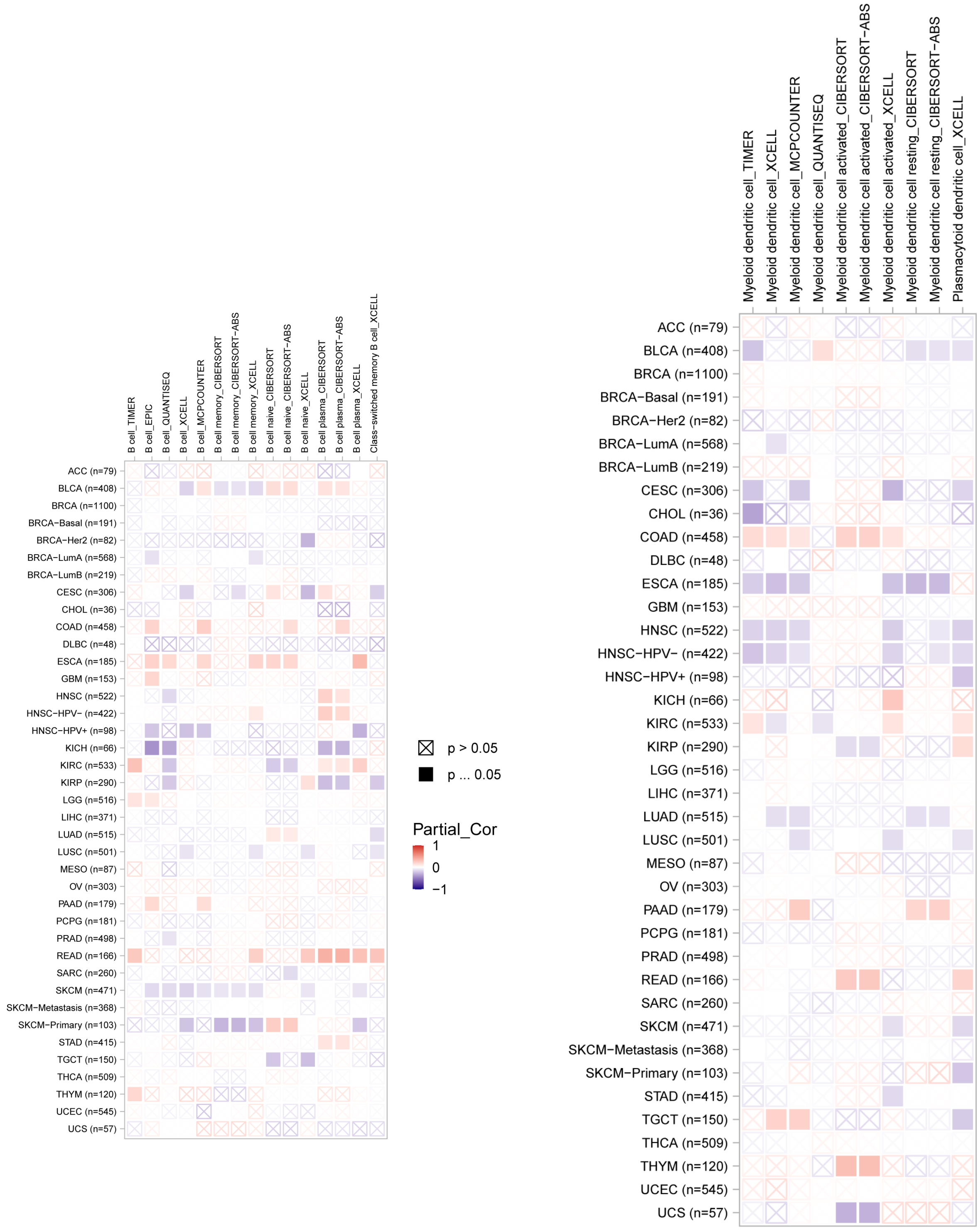

B cell immune infiltration level

DC cell immune infiltration level

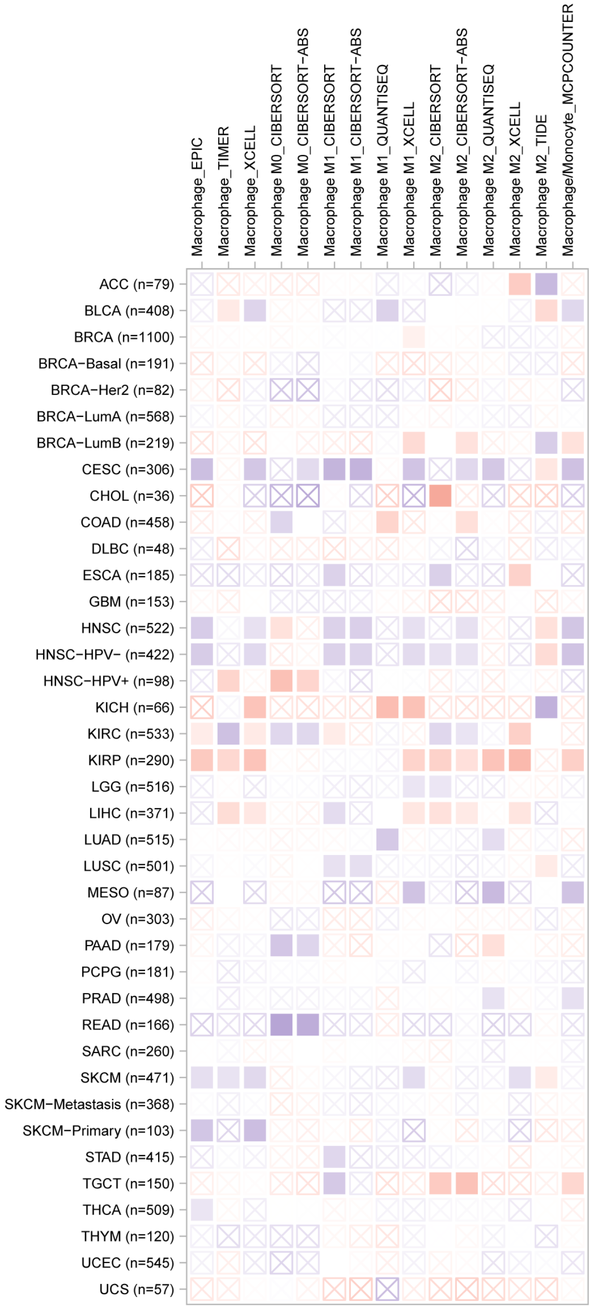

Macrophage immune infiltration level

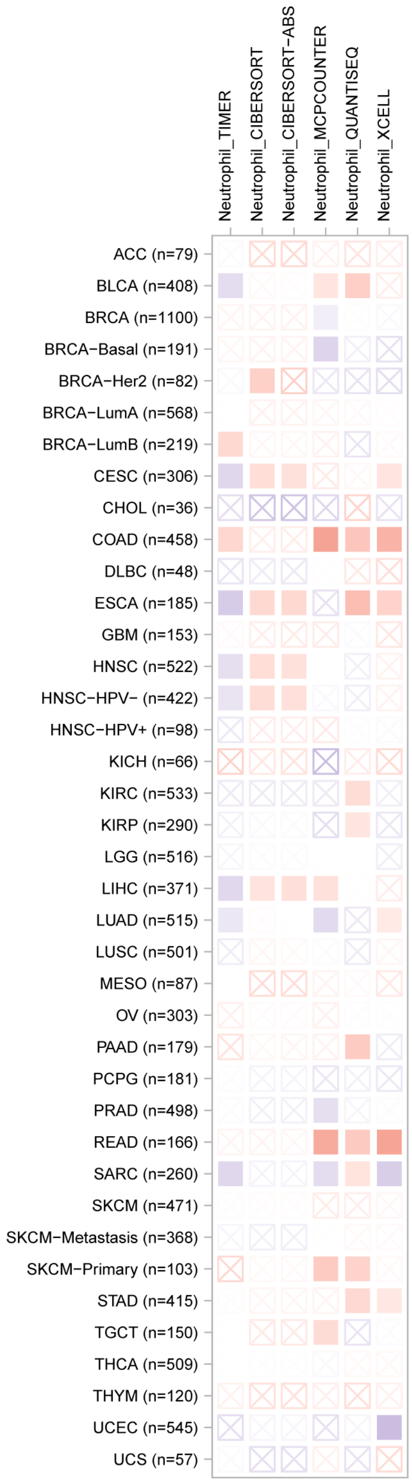

Neutrophil immune infiltration level

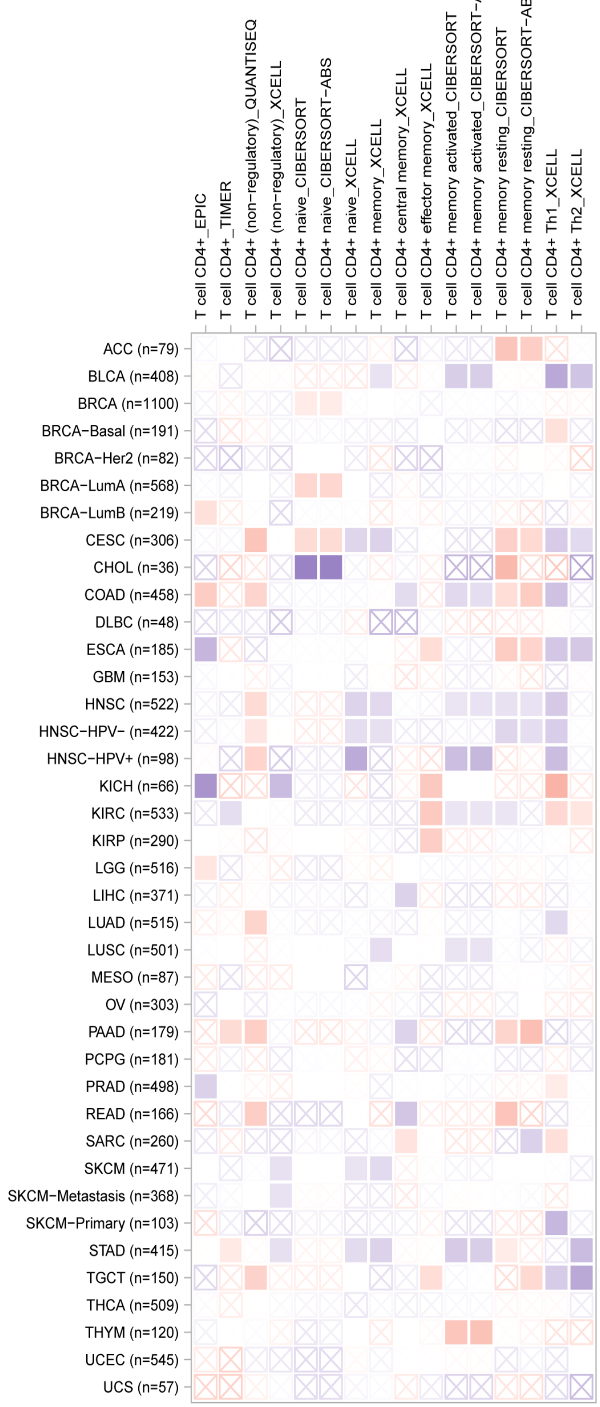

T cell CD4+ immune infiltration level

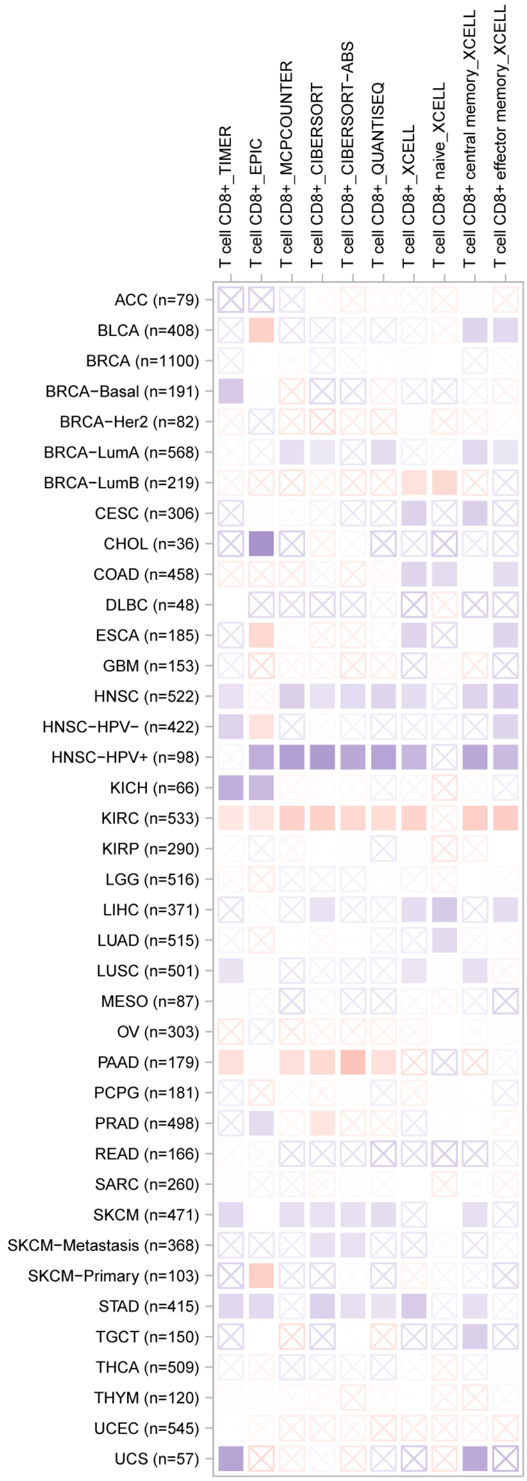

T cell CD8+ immune infiltration level

Relationship of CXCL8 expression with immune infiltration level in diverse cancer types (TIMER 2.0).

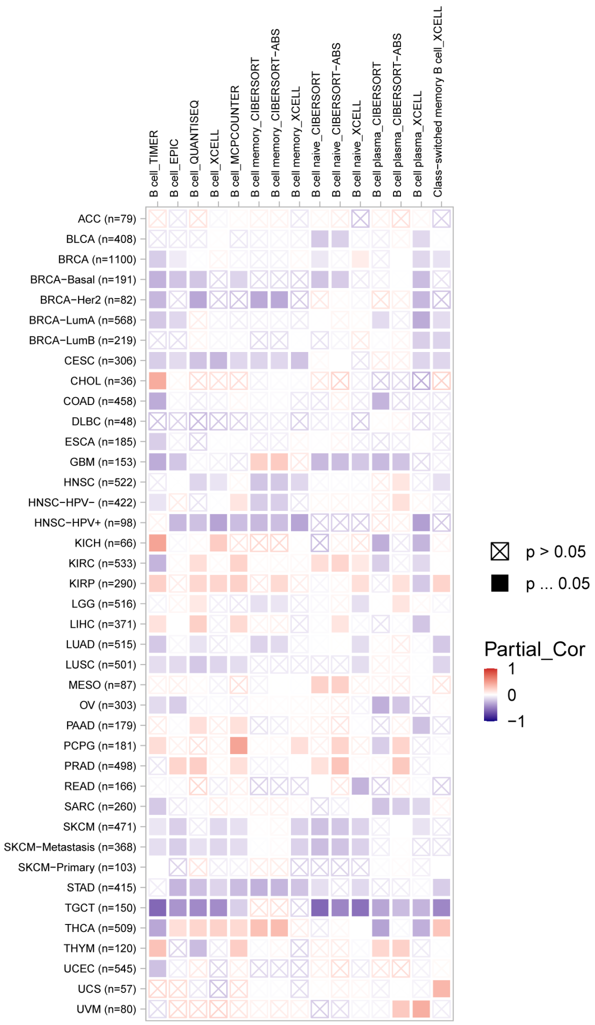

B cell immune infiltration level

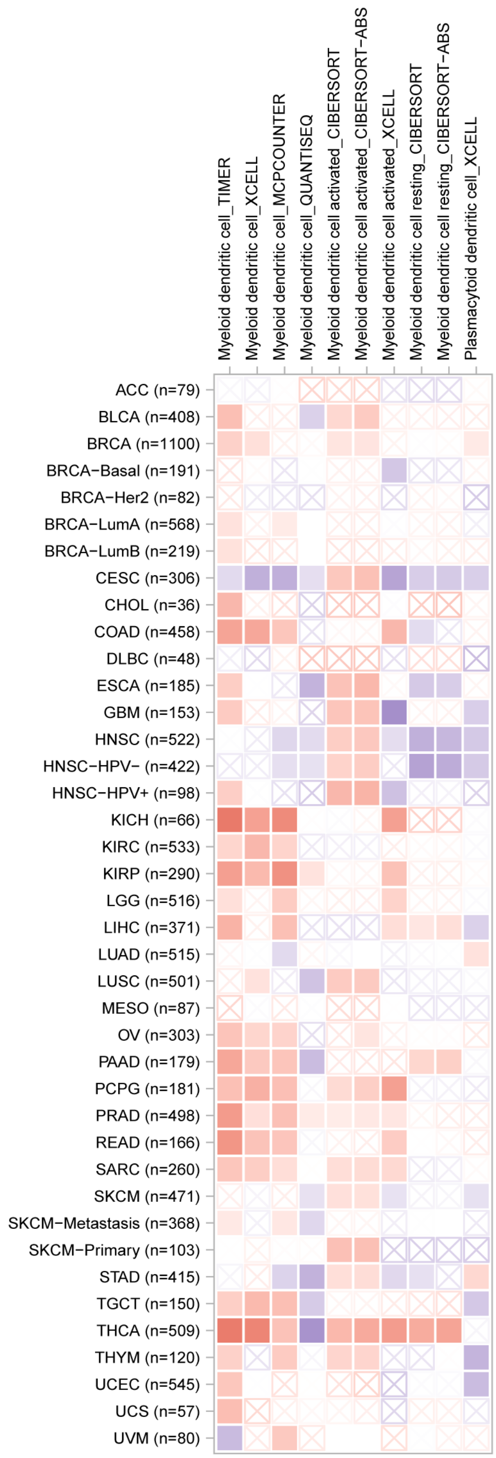

DC cell immune infiltration level

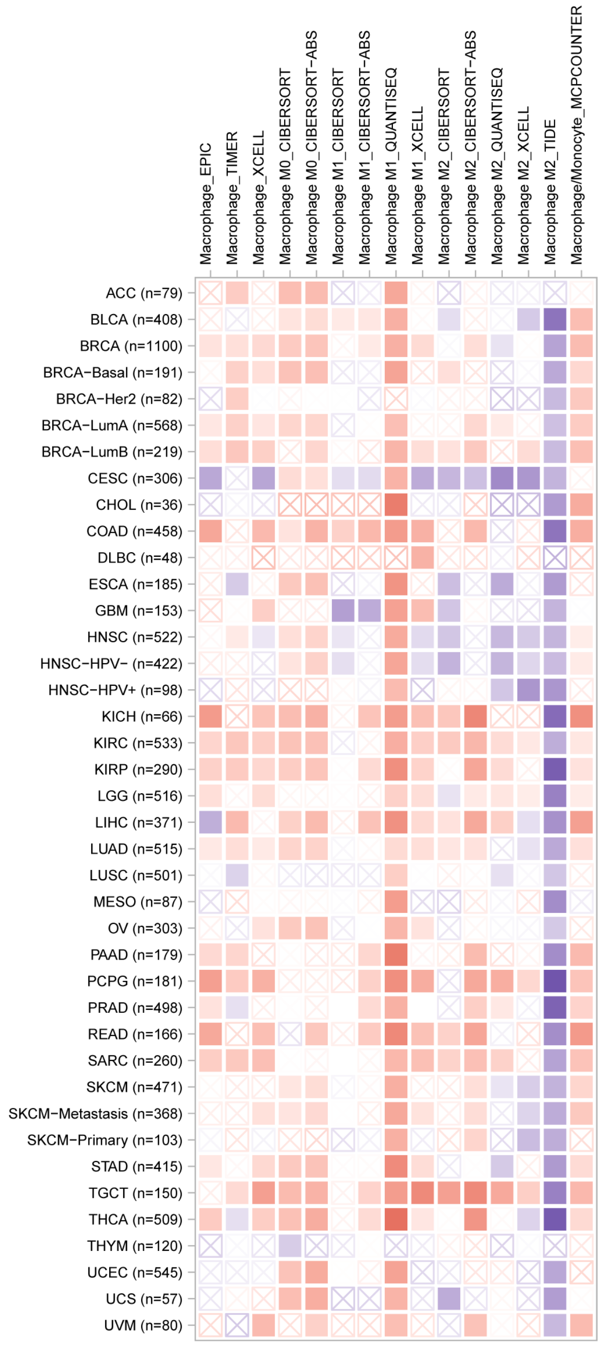

Macrophage immune infiltration level

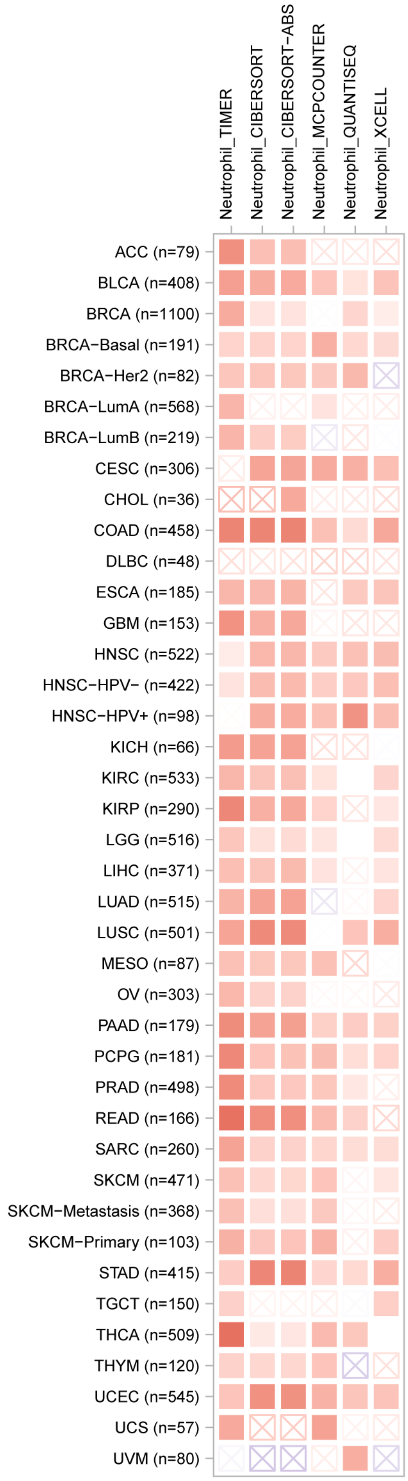

Neutrophil immune infiltration level

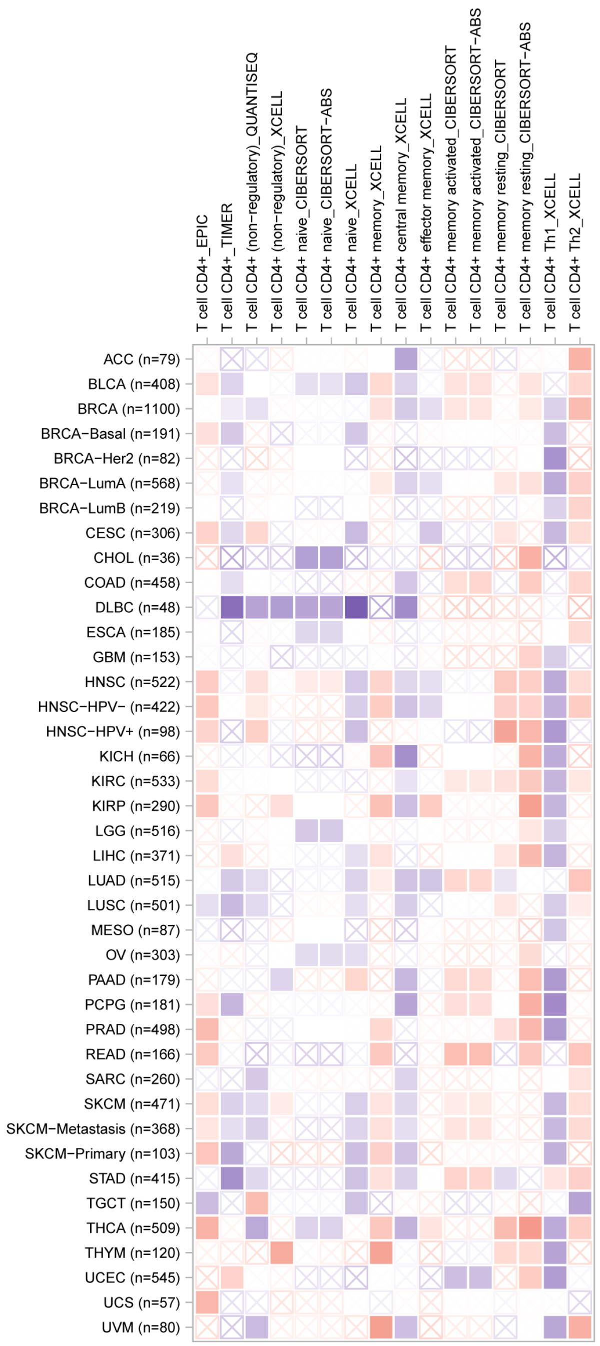

T cell CD4+ immune infiltration level

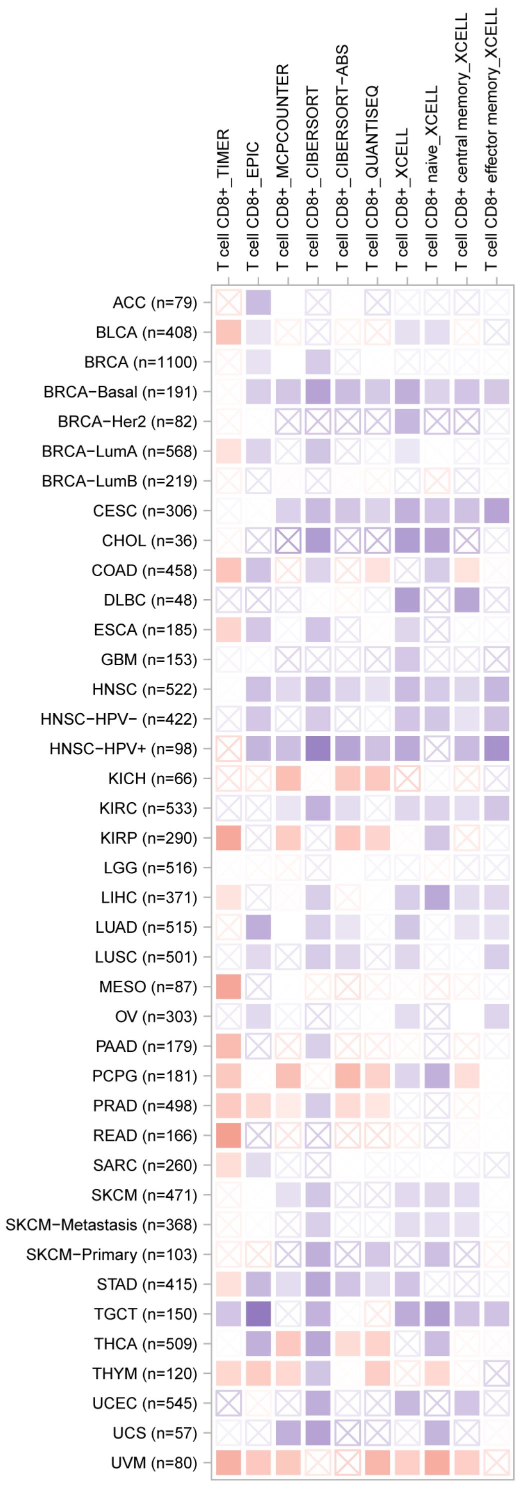

T cell CD8+ immune infiltration level

Relationship of CEMIP expression with immune infiltration level in diverse cancer types (TIMER 2.0).

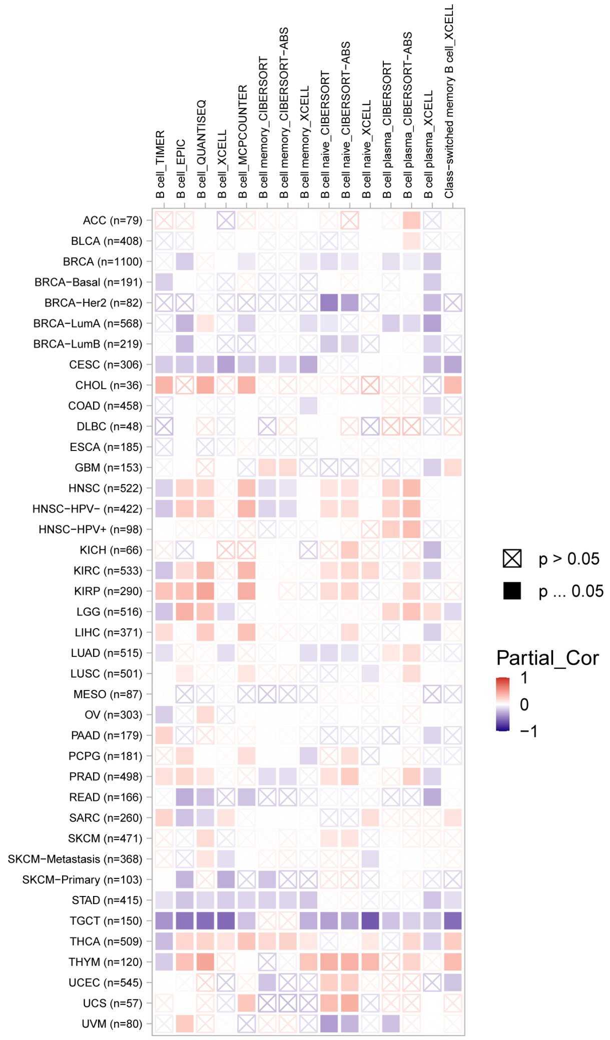

B cell immune infiltration level

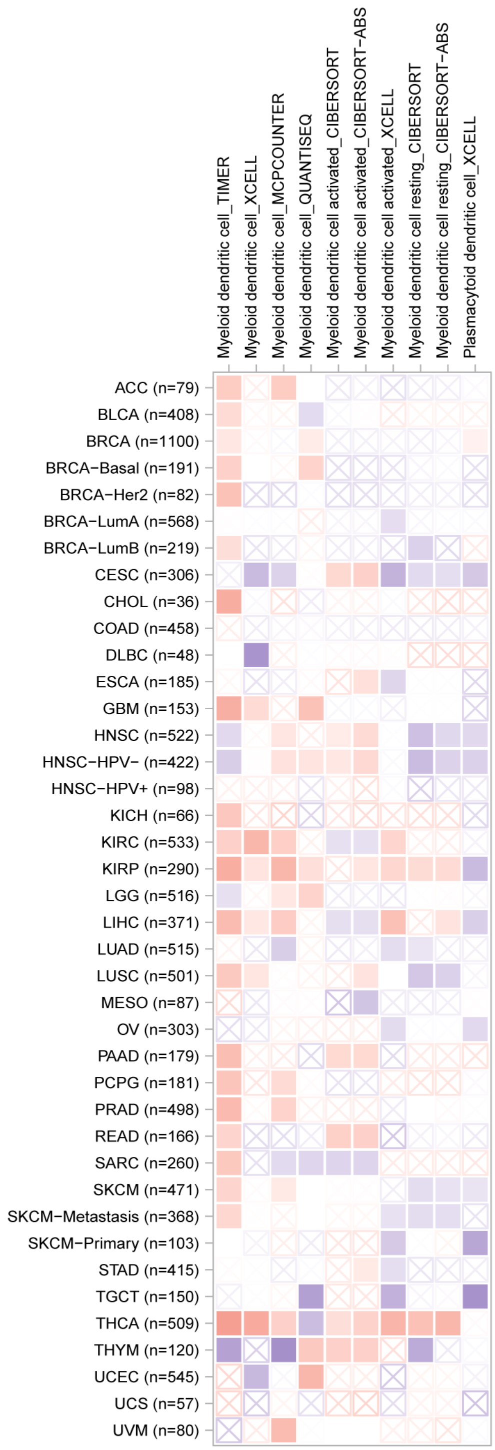

DC cell immune infiltration level

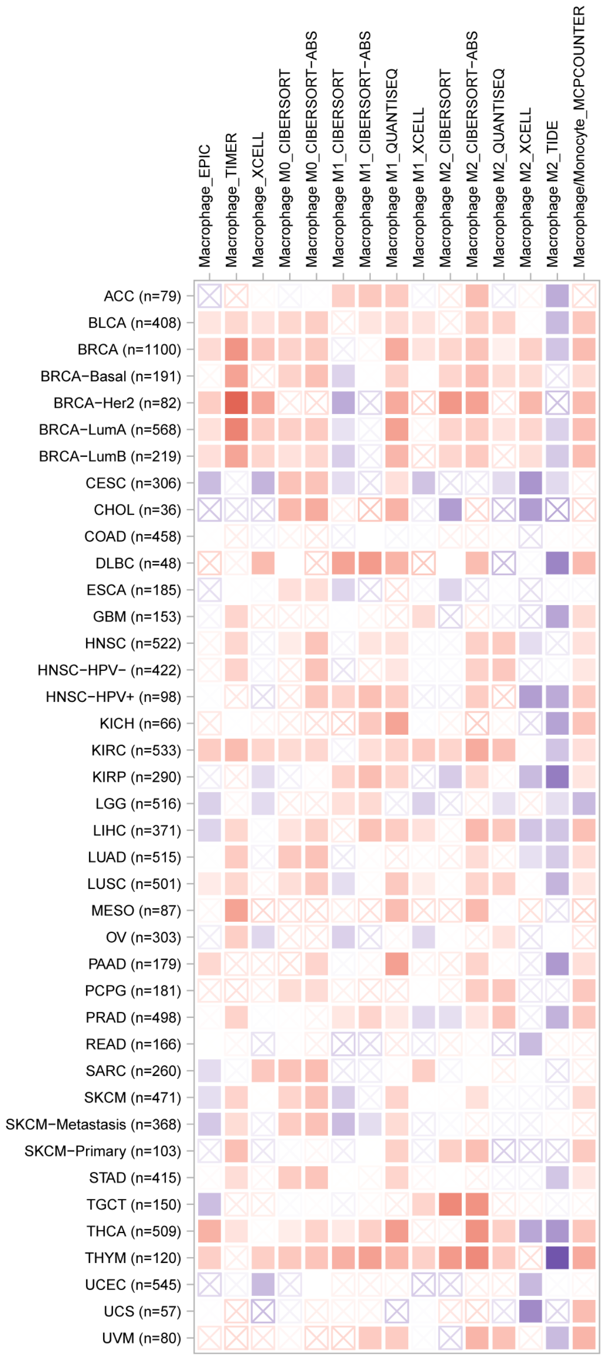

Macrophage immune infiltration level

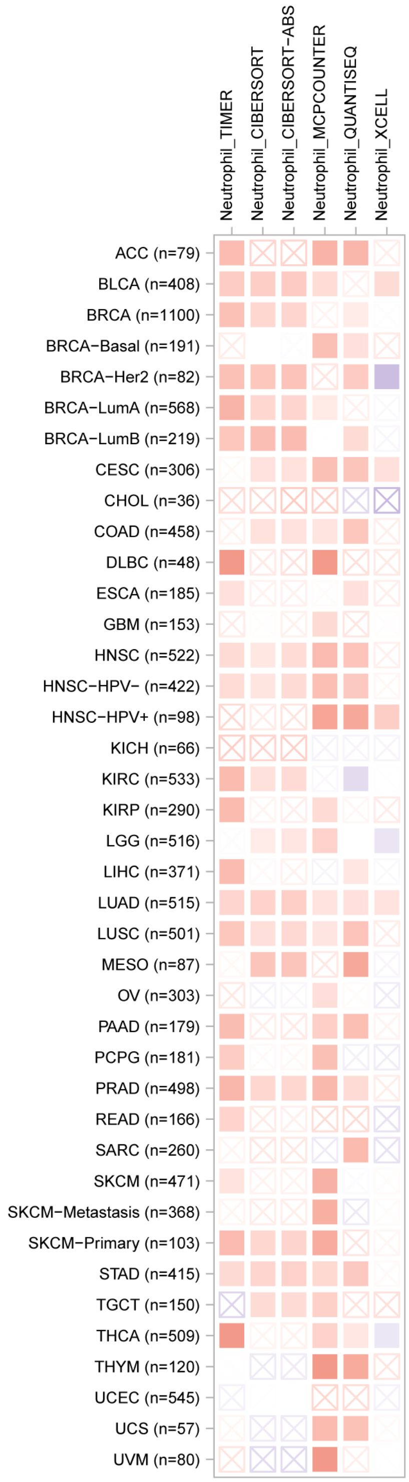

Neutrophil immune infiltration level

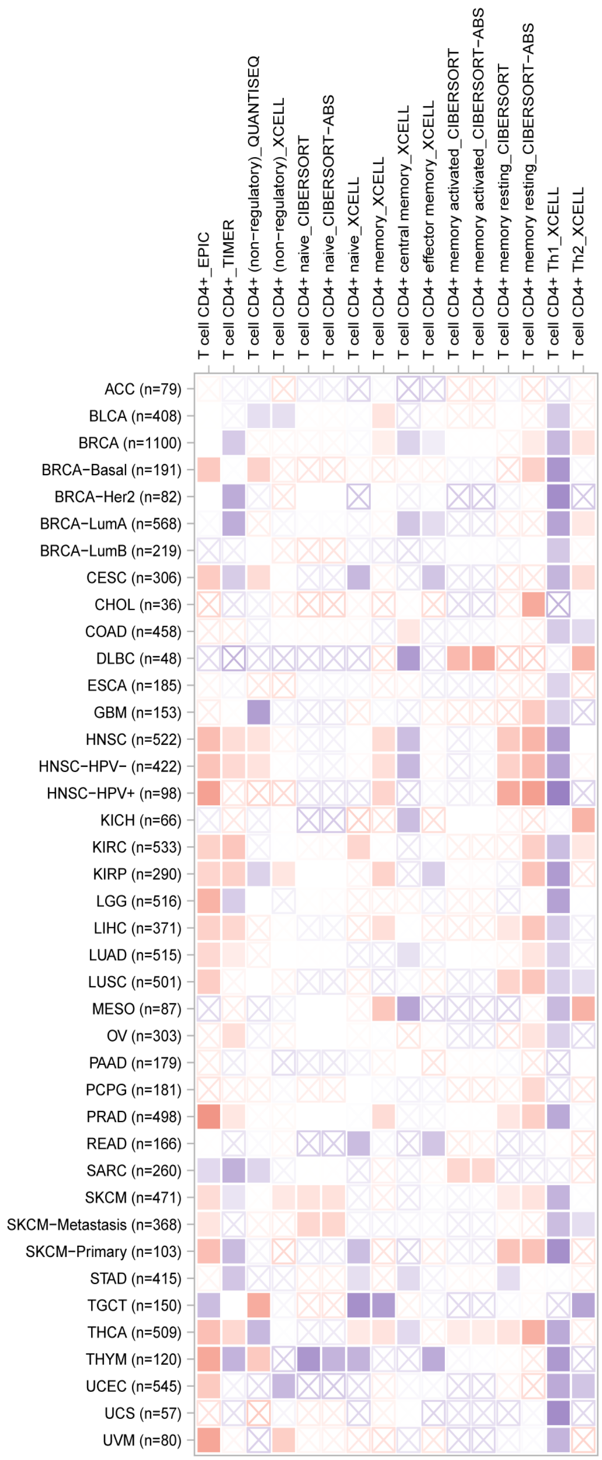

T cell CD4+ immune infiltration level

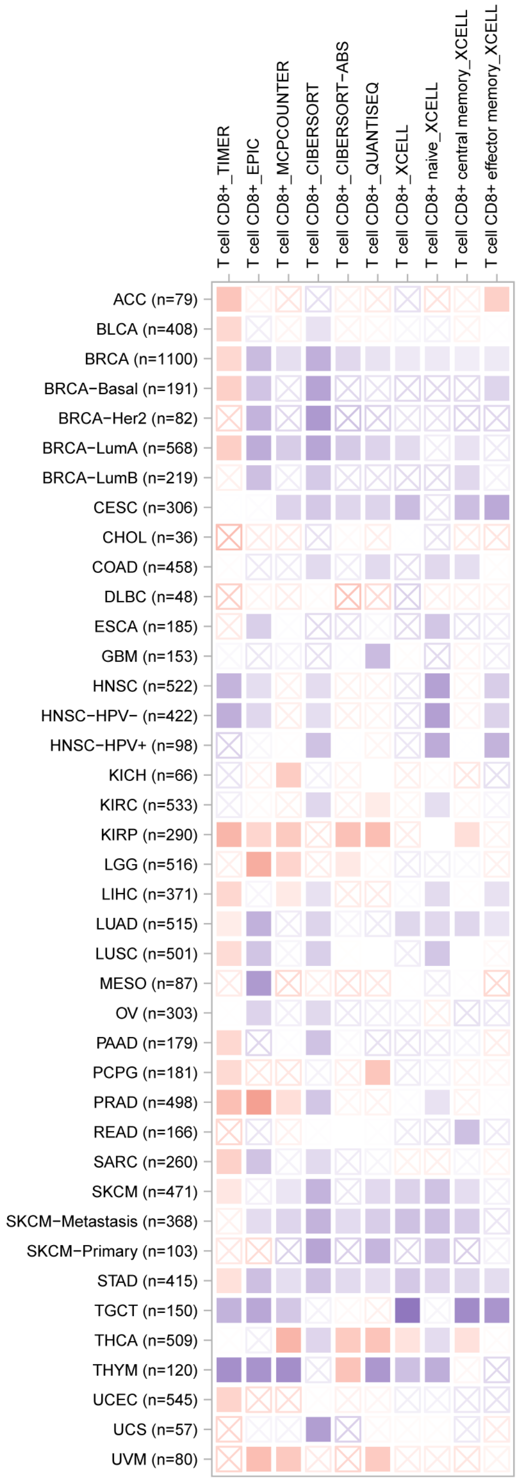

T cell CD8+ immune infiltration level

Relationship of MMP7 expression with immune infiltration level in diverse cancer types (TIMER 2.0).

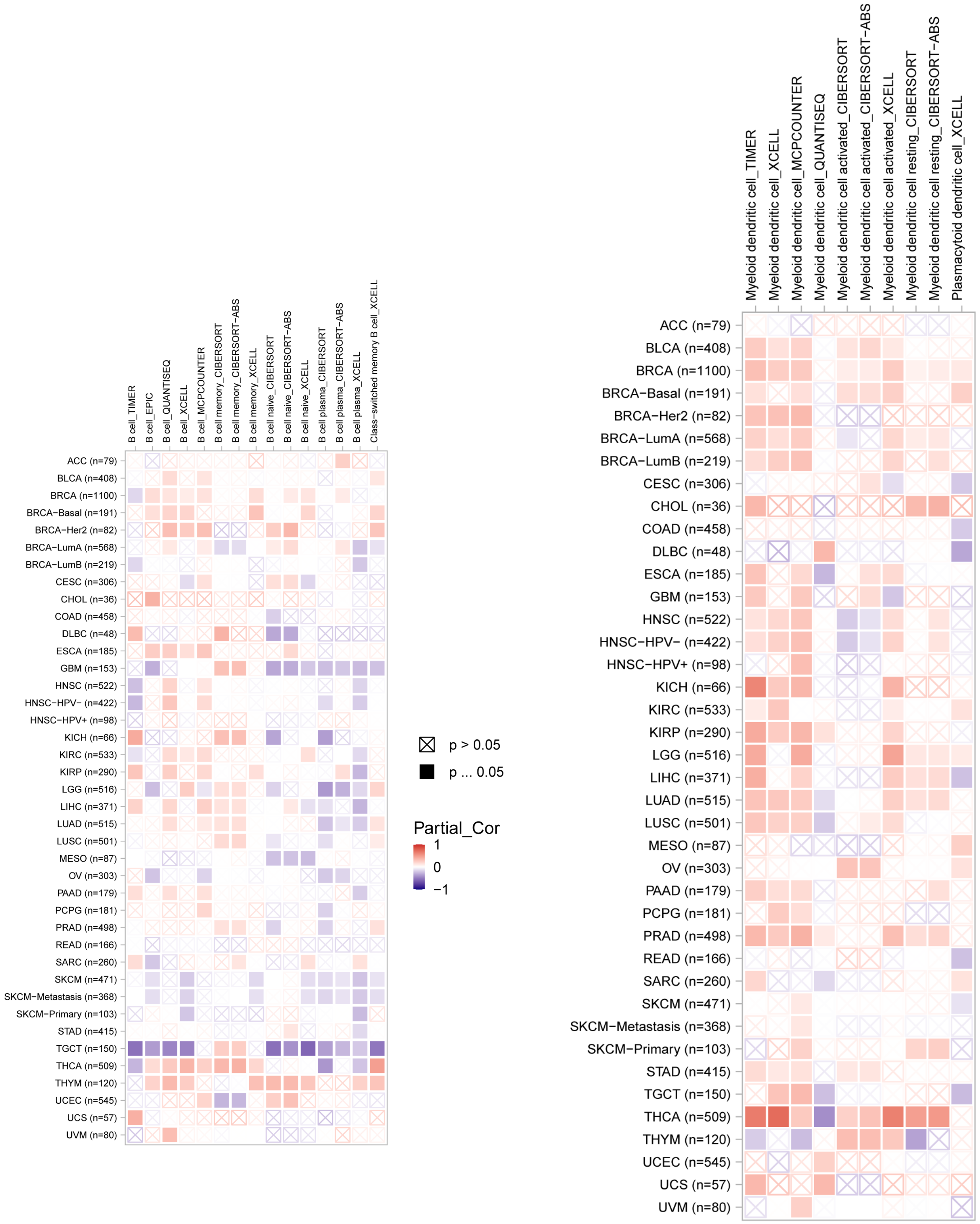

B cell immune infiltration level

DC cell immune infiltration level

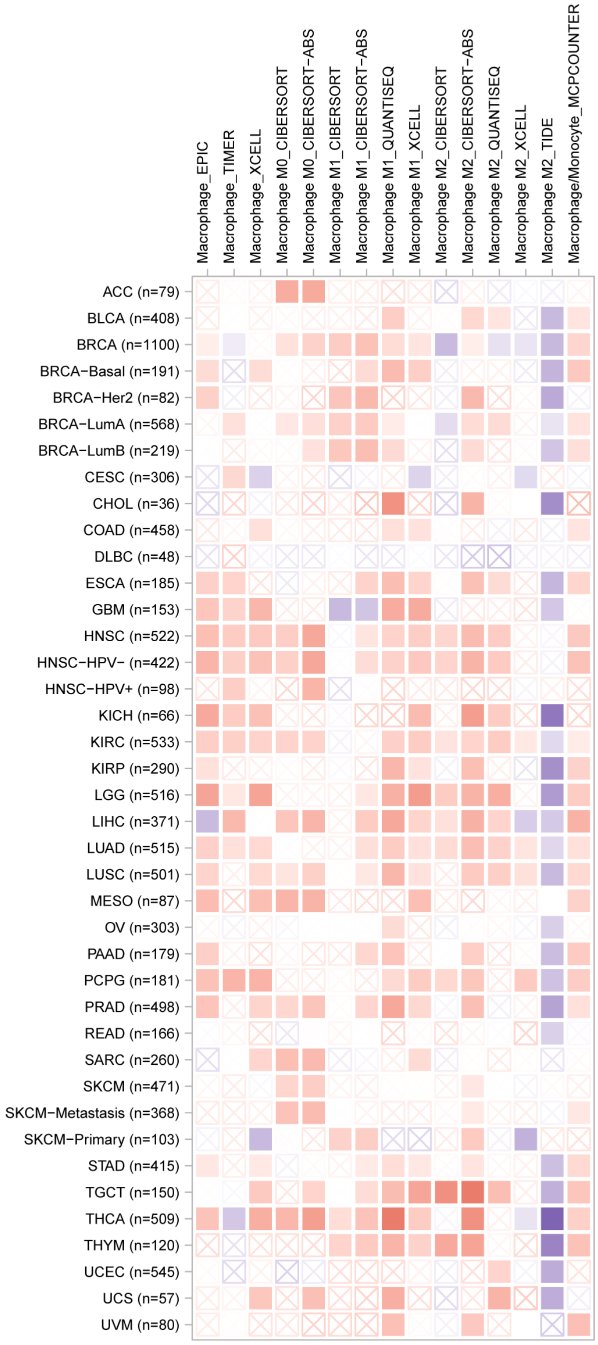

Macrophage immune infiltration level

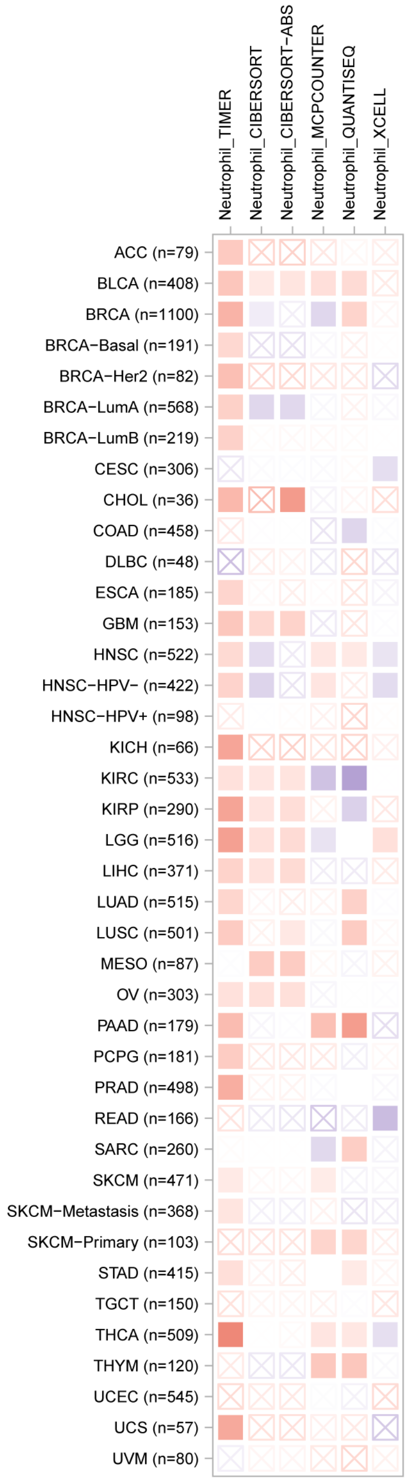

Neutrophil immune infiltration level

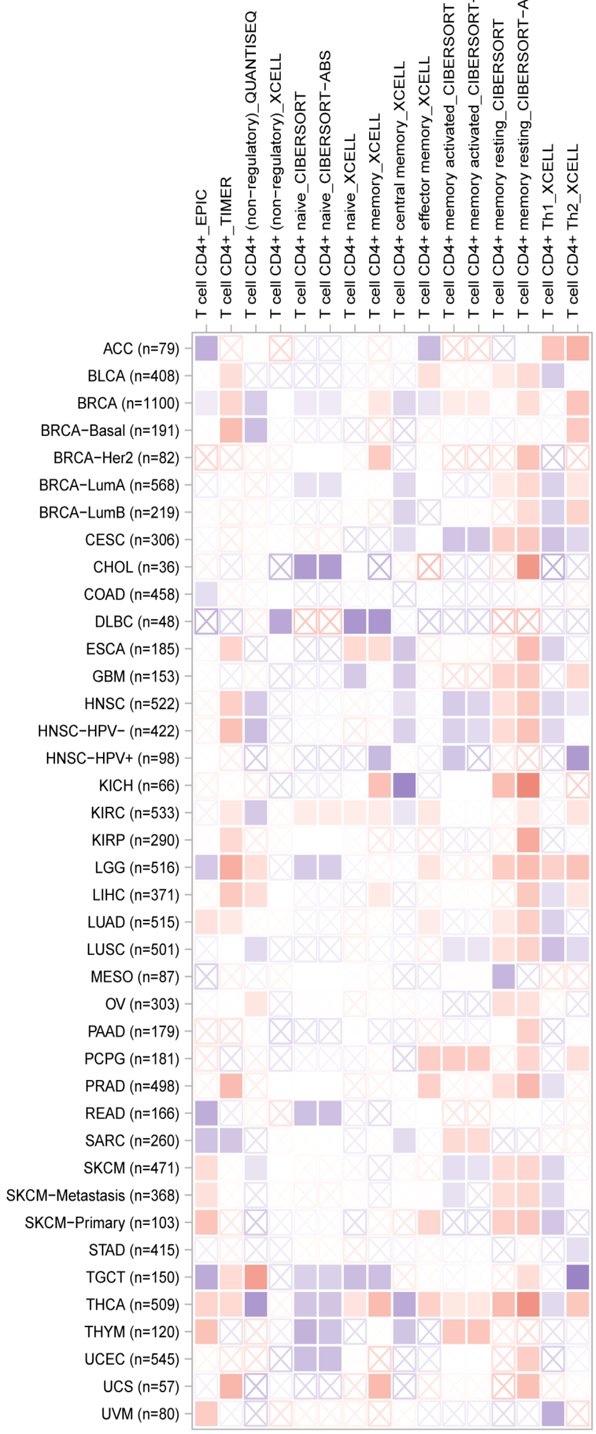

T cell CD4+ immune infiltration level

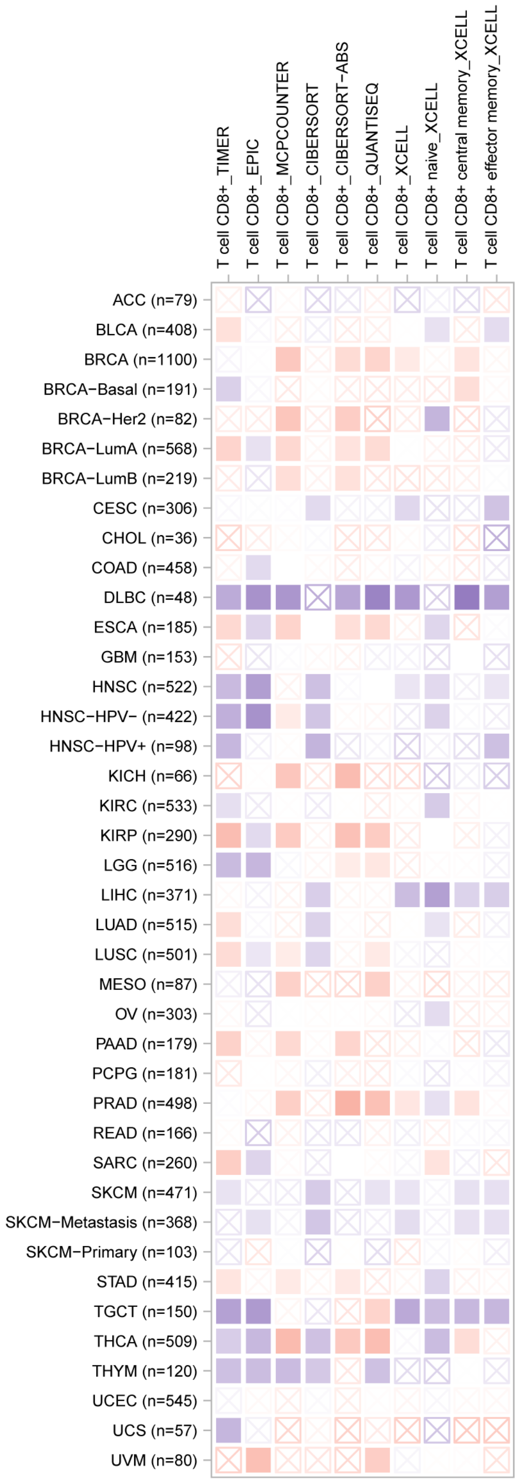

T cell CD8+ immune infiltration level

Relationship of ZG16 expression with immune infiltration level in diverse cancer types (TIMER 2.0).

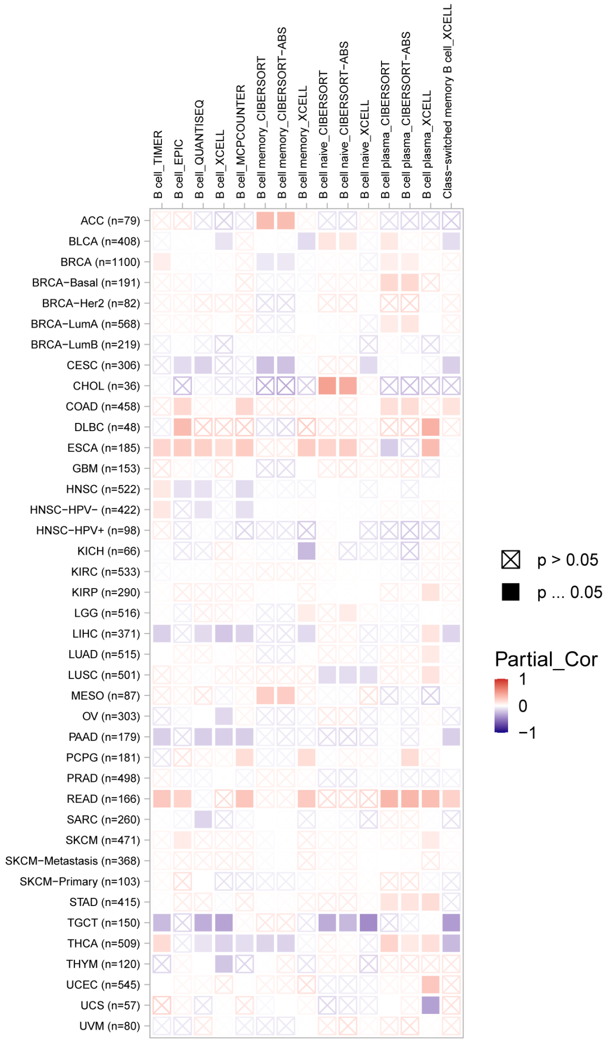

B cell immune infiltration level

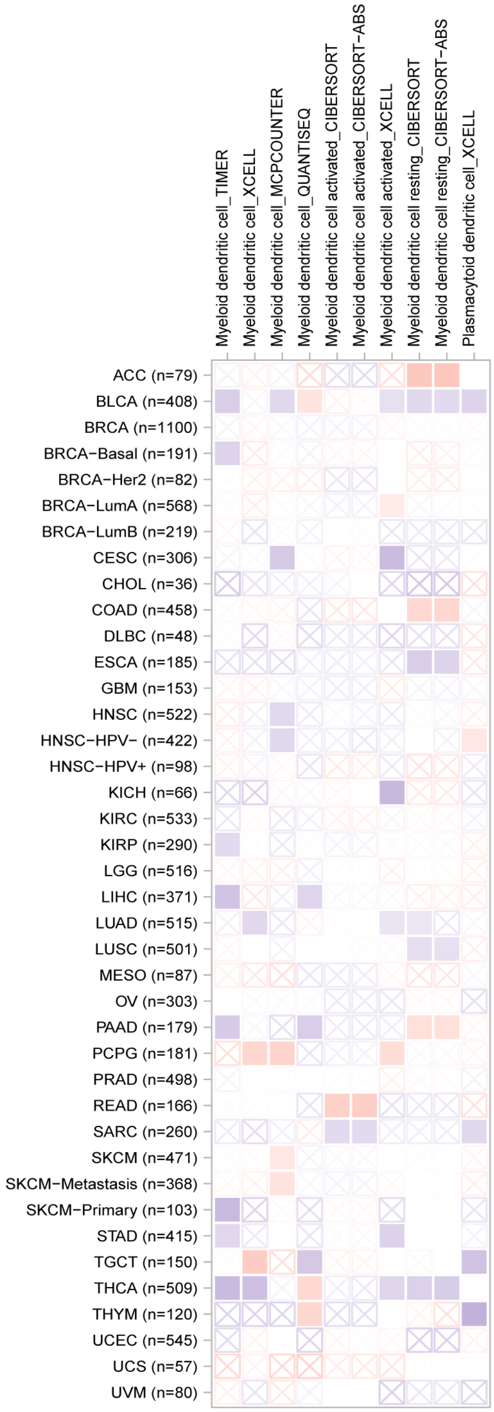

DC cell immune infiltration level

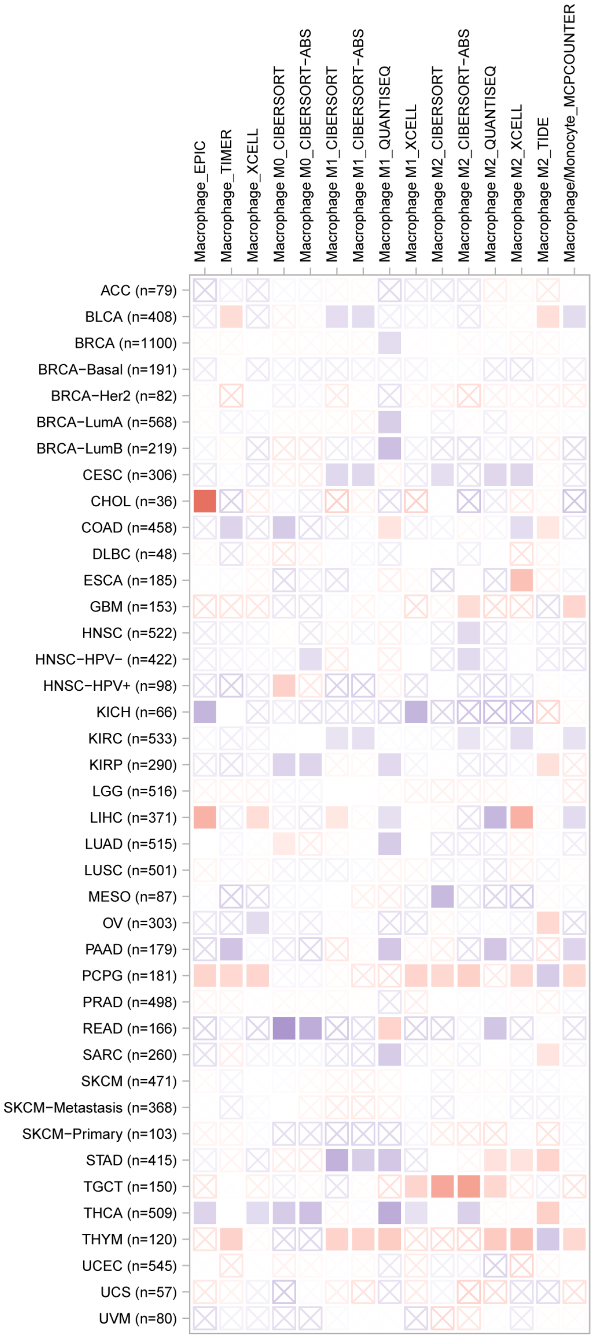

Macrophage immune infiltration level

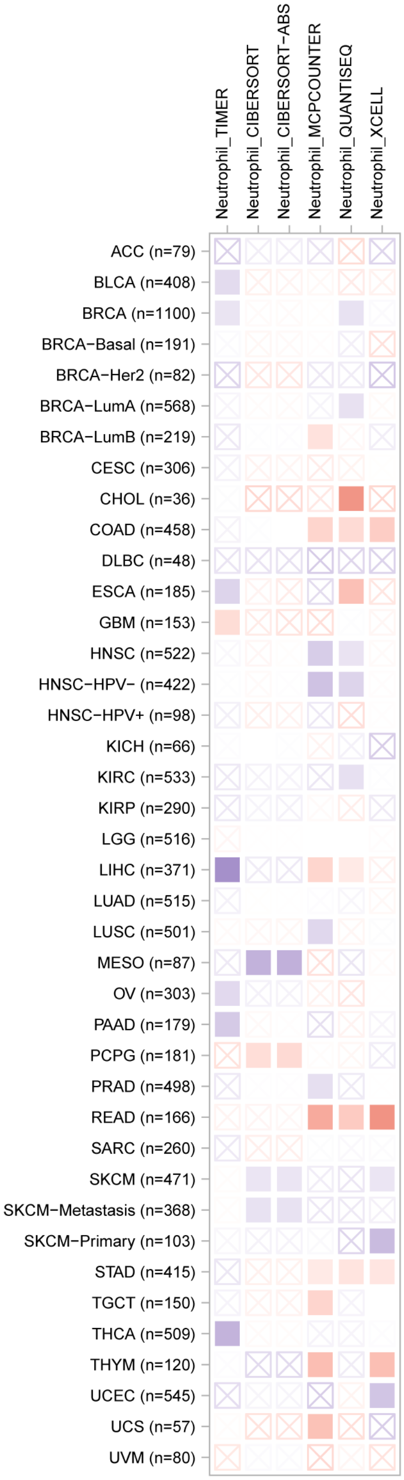

Neutrophil immune infiltration level

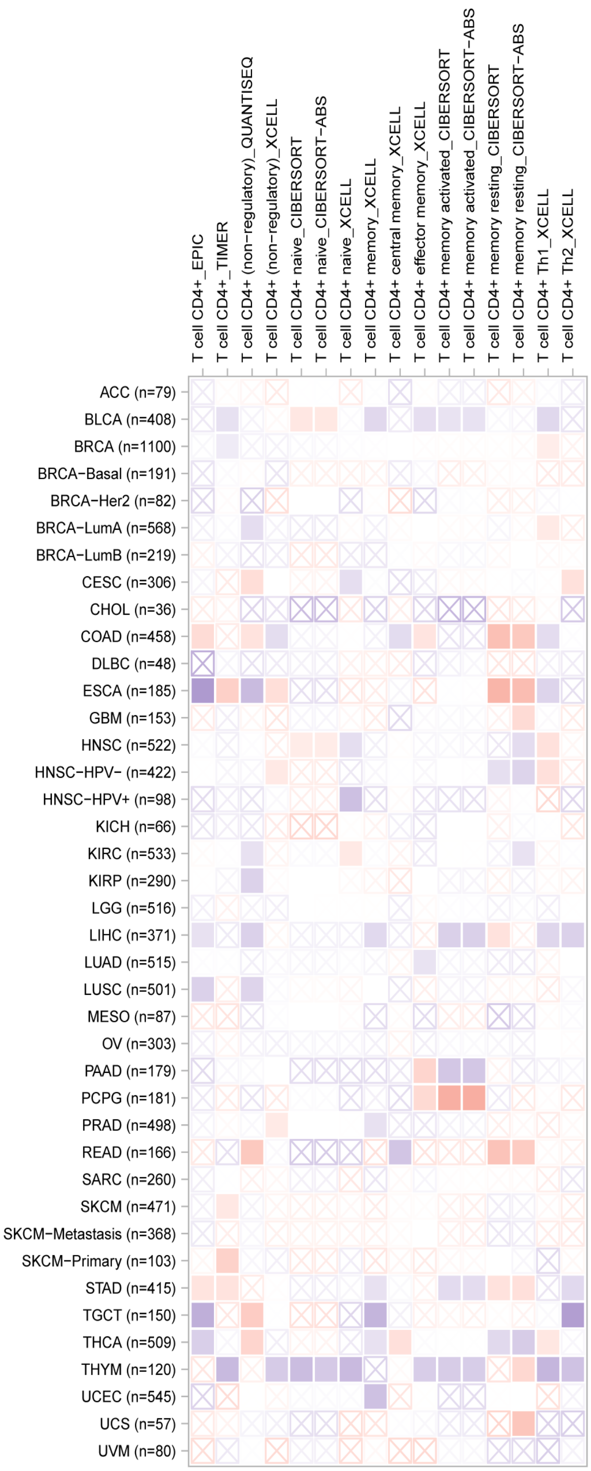

T cell CD4+ immune infiltration level

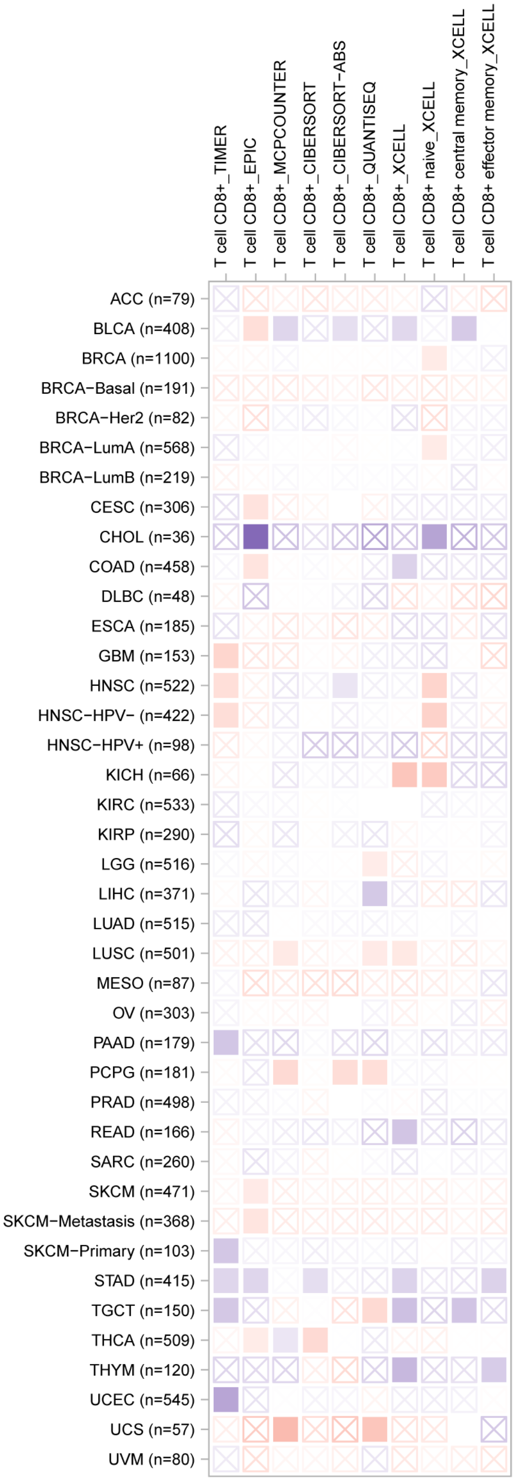

T cell CD8+ immune infiltration level
